# Magnetic Properties Tuning via Broad Range Site Deficiency in Square Net Material UCu_*x*_Bi_2_

**DOI:** 10.1021/jacs.4c18438

**Published:** 2025-04-22

**Authors:** Hope A. Long, Daniel Duong, Joanna Blawat, Gregory Morrison, Yan Wu, Huibo Cao, Nabaraj Pokhrel, David S. Parker, John Singleton, Rongying Jin, Vladislav V. Klepov

**Affiliations:** † Department of Chemistry, University of Georgia, Athens, Georgia 30602, United States; ‡ SmartState Center for Experimental Nanoscale Physics, Department of Physics and Astronomy, University of South Carolina, Columbia, South Carolina 29208, United States; § NHMFL, Los Alamos National Laboratory, MS E536, Los Alamos, New Mexico 87545, United States; ∥ Center for Hierarchical Waste Form Materials, and Department of Chemistry and Biochemistry, University of South Carolina, Columbia, South Carolina 29208, United States; ⊥ Neutron Scattering Division, Oak Ridge National Laboratory, Oak Ridge, Tennessee 37831, United States; # Materials Science and Technology Division, Oak Ridge National Laboratory, Oak Ridge, Tennessee 37831, United States

## Abstract

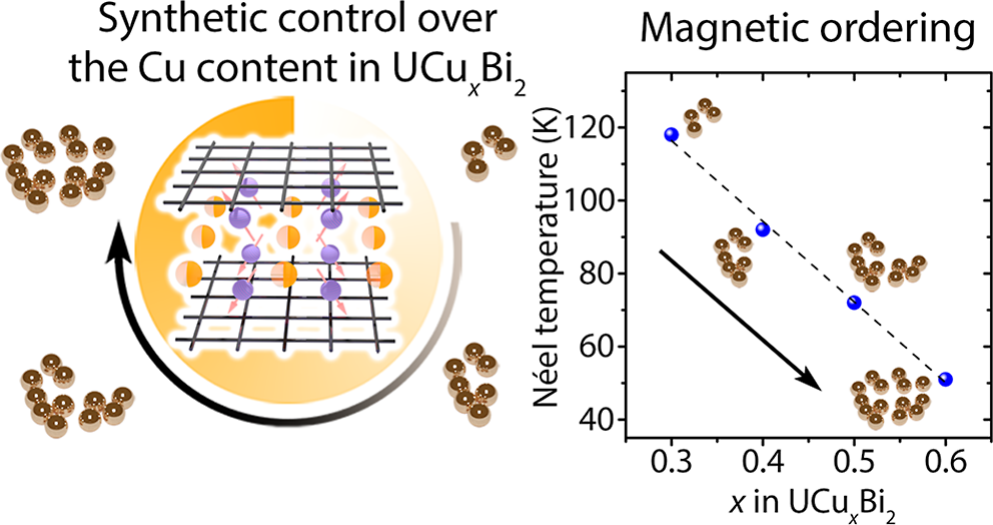

HfCuSi_2_-type pnictogen compounds have recently been shown to be a versatile platform for designing materials with topologically nontrivial band structures. However, these phases require strict control over the electron count to tune the Fermi level, which can only be achieved in compositions with A^2+^M^2+^Pn_2_ and A^3+^M^+^Pn_2_ (A = lanthanides, M = transition metals, Pn = pnictogens P–Bi) charge distribution. While such lanthanide compounds have been thoroughly studied as candidate magnetic topological materials, their heavy element analogs with uranium and bismuth remain largely underexplored. In this report, we present the synthesis of UCu_*x*_Bi_2_ single crystals and study their magnetic properties. Detailed structural analysis revealed that flux-grown crystals always form as a site-deficient UCu_*x*_Bi_2_ composition, where *x* varies between 0.20 and 0.64. Magnetic property measurements revealed a dependence of the magnetic coupling on the Cu site deficiency, linearly changing the Néel temperature from 51 K for UCu_0.60_Bi_2_ to 118 K for UCu_0.30_Bi_2_. Moreover, higher Cu concentration promotes a metamagnetic transition in highly magnetically anisotropic UCu_0.60_Bi_2_ single crystals. We show that DFT calculations can successfully model site deficiency in the UCu_*x*_Sb_2_ and UCu_*x*_Bi_2_ systems. This work paves the way toward using the site deficiency to tune the Fermi level in more ubiquitous A^3+^M^2+^_*x*_Pn_2_ phases, which previously have not been considered topological candidate materials due to unfavorable electron count.

## Introduction

The realization of quantum states in condensed matter has sparked extensive research on topological phases, i.e. compounds in which physical properties are protected by the topology of their band structure.^1−5^ Topological materials have a unique ability to mimic high energy particles and offer an extensive playground to study emergent physical phenomena,^6−8^ such as ultrahigh carrier mobility,^9,10^ giant magnetoresistance,^11,12^ and quantum spin Hall effect.^13,14^ These properties are commonly hosted in topological insulators, Dirac, and Weyl semimetals.^3,15^ The latter two classes represent electronic analogs of graphene, whose main electronic feature is the presence of linearly dispersed bands that cross at Dirac points in their band structure.^16−18^ Because of the linear dispersion, the behavior of charge carriers at Dirac points is described by the Dirac equation, giving rise to unique massless Fermions.^16^ The discovery of 3D analogs of graphene, Na_3_Bi and Cd_3_As_2_,^19−22^ opened a pathway to a new class of materials, which are currently attracting great interest for their potential applications in spintronics and optical devices.^23−26^

Since the band structure of a material is mainly defined by the nature of its constituent elements and their mutual arrangement in a crystal structure, the realization of some specific structural features and symmetries in a compound inherently gives rise to topological properties.^27,28^ One such structural feature is a square net of main group atoms, in which overlap between p_*x*_ and p_*y*_ orbitals results in linearly dispersed bands along the Γ-Μ and Γ-Χ symmetry lines.^29−31^ These band intersections remain largely unaffected by other bands as long as the square net atoms are well separated from other atoms in the crystal structure. To quantify this separation, Schoop et al. proposed a tolerance factor, *d*_sq_/*d*, which shows the ratio between the distances within the square nets, *d*_sq_, and between the square net atoms and other atoms in the structure, *d*.^31^ For phases with low tolerance factors *d*_sq_/*d* < 0.95, the square nets are considered sufficiently isolated from other atoms to give rise to rather “clean” Dirac points.^15^ One example is ZrSiS, which exhibits a Si square net separated from other atoms.^32^ In agreement with theoretical predictions, angle-resolved photoemission spectroscopy (ARPES) measurements on ZrSiS single crystals demonstrated a Dirac crossing in this compound. Since square nets are a common feature of several other structure types related to the PbFCl-type, these phases became the forefront of the search for new topological semimetal materials.^33,34^

HfCuSi_2_ is a common structure type that is comprehensively studied for topological semimetal properties. This structure represents a “stuffed” PbFCl-type, consisting of Si square net layers that are separated by corrugated (HfSi) insertions and Cu atoms. HfCuSi_2_-type is a very versatile platform for compositional tuning. It can adopt various elements, including Ca, Sr, lanthanides, and some actinides on the Hf site, transition metals on the Cu site, and pnictogens P–Bi on the Si site. Pnictides are the most represented compounds in the HfCuSi_2_-type and have demonstrated interesting physical properties stemming from an interplay between band topology and high magnetic anisotropy of the lanthanide cations. Recently reinvigorated interest in these phases, due to their potential topological semimetal properties, resulted in detailed studies of several phases with magnetic ordering, including CaMnBi_2_,^35^ SrMnBSb_2_,^36^ EuMnPn_2_ (Pn = Sb and Bi),^37,38^ BaMnSb_2_,^39−41^ EuZnSb_2_,^42^ SrZnSb_2_,^43^ YbMnPn_2_ (Pn = Sb and Bi),^44−47^ LaCuSb_2_,^48^*Ln*AgSb_2_ (Ln = Y, La–Nd, Sm),^49^ and LaAgBi_2_.^50^ Since the manifestation of topological properties requires the Dirac points to be near the Fermi level, HfCuSi_2_-type phases are sensitive to electron filling. The optimal electron count that places the Fermi level at Dirac points is six, corresponding to fully occupied s and p_z_ orbitals and half-filled p_*x*_ and p_*y*_ orbitals of the square net atoms.^31^ Assuming a −3 oxidation state on nonsquare net pnictide atoms,^51^ this electron count can be achieved by employing lanthanide and transition metal cations that give a +4 charge in total, thus favoring Ln^2+^M^2+^Pn^3–^(Pn^–1^)_sq_ or Ln^3+^M^+^Pn^3–^(Pn^–1^)_sq_ compositions. Though the variability of the M site occupancy has been well established for these systems, its effect on the resulting physical properties has been less well investigated. While the Hf site has been primarily considered a host for lanthanide cations, it can also be occupied by uranium. As UAuBi_2_ has demonstrated physical behavior consistent with the +3 oxidation state,^52^ U^3+^M^+^Pn_2_ phases can also display a favorable electron count. However, many such compositions with typically +1 transition metals, e.g., UCuSb_2_ and UCuBi_2_, have only been briefly characterized as powders,^53−55^ leaving many of their physical properties unknown. Of those that have been thoroughly characterized, a majority report the occurrence of only one magnetic phase transition.^53,56^ However, cases like that of UAu_0.8_Sb_2_ reveal unique magnetic behavior in which multiple field-induced magnetic transitions are observed.^57^ Despite intriguing magnetic properties, their dependence on the M site deficiency remains largely underexplored, especially for U-containing phases.

**Figure 1:**
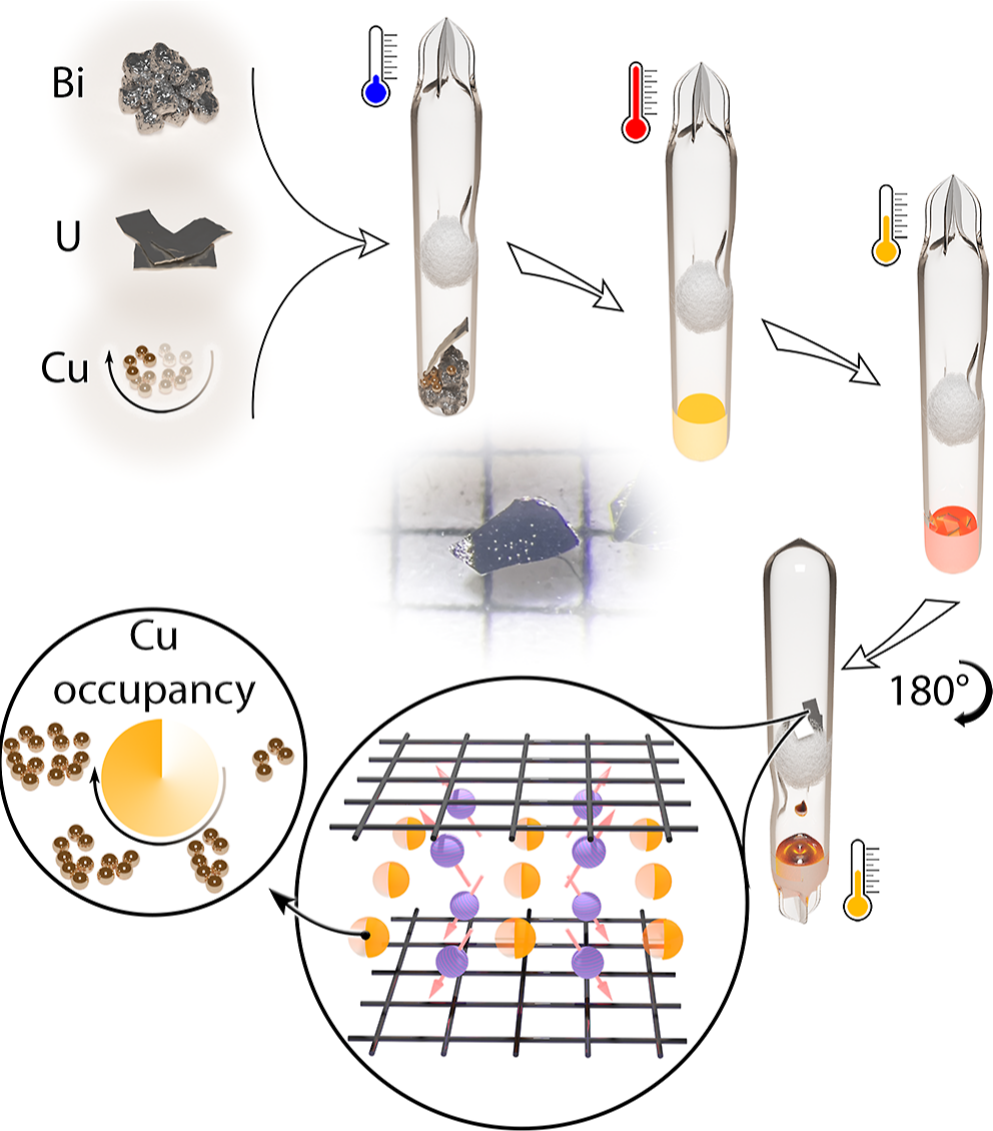
Schematic representation of the synthesis of UCu_*x*_Bi_2_ crystals. Cu content in the final product can be adjusted by varying Cu ratio in the starting mixture, which affects the properties of the resulting phase. The central image shows a grown crystal of UCu_*x*_Bi_2_ on a millimeter paper.

In this report, we present the synthesis of HfCuSi_2_-type single crystals in the U–Cu–Bi system and the characterization of their properties. Although UCuBi_2_ has been reported as a stoichiometric 1:1:2 compound before, our multiple synthetic attempts yielded only Cu-deficient crystals of UCu_*x*_Bi_2_ with a Cu content *x* varying between 0.20 and 0.64. Despite the Cu site deficiency diverting the electron count from an ideal one, we used this system as a platform to study the synthetic control over the transition metal content that can enable tunability of Fermi level and physical properties in ubiquitous A^3+^M^2+^_*x*_Pn_2_ phases (Figure 1). We also developed a computational method to model the site-deficient phases and performed density functional theory (DFT) calculations to predict their stability. Overall, this report paves the path toward HfCuSi_2_-type phases with outstanding compositional tunability that can be employed for adjusting the Fermi level in magnetic topological materials by site deficiency control.

## Results and Discussion

### Crystal Growth and Crystal Structure

We have performed a series of reactions to find conditions that result in sufficiently large single crystals for property measurements. Since metal fluxes show great success for growing intermetallic uranium crystals,^58−60^ excess Bi was employed as flux. As some HfCuSi_2_-type phases have exhibited M site deficiency,^61−63^ we used an excess of Cu in the starting reaction mixture to achieve the maximum Cu loading. A reaction with a 1:3:13 U/Cu/Bi molar ratio yielded single crystals of the target UCu_*x*_Bi_2_ phase with *x* ≈ 0.6, which were surface-contaminated with octahedral Cu crystals. Increased amounts of Bi flux by ∼50 and 100% (U/Cu/Bi ratios of 1:3:19 and 1:3:27) yielded significantly larger crystals with less surface contamination (Figure S1). To reduce site deficiency, we attempted to increase Cu content in the reaction to a 1:4:19 ratio, which led to more Cu crystals on the surface with no significant change in the final composition of the target phase. Interestingly, the crystals grown in niobium tubes had noticeably cleaner surfaces than those grown using alumina crucibles, but they were significantly smaller in size. Optimized crystal growth in alumina crucibles resulted in large plate single crystals, up to 3 × 3 × 0.3 mm^3^, that were suitable for various properties measurements.

Additional crystal growth experiments with lower amounts of Cu in the starting material showed that the Cu content in the target phases can vary widely. A reaction with a 1:1:19 ratio of U/Cu/Bi yielded UCu_*x*_Bi_2_ crystals with *x* ranging between 0.37 and 0.54, as determined by single crystal X-ray diffraction. Further decrease of the Cu content by using a ratio of 1:0.5:19 resulted in crystals with *x* from 0.20 to 0.36. However, we observed the formation of a competing UBi_2_ binary phase with such low Cu concentrations. While there is an overall trend of reducing *x* in UCu_*x*_Bi_2_ as the amount of Cu in the starting reaction decreases, the Cu content changes among crystals within the same batch, likely due to gradual Cu concentration and temperature changes during crystal growth. Since crystal growth occurs over a wide temperature range, one can surmise that Cu content can also be inhomogeneous in large single crystals of UCu_*x*_Bi_2_.

**Figure 2:**
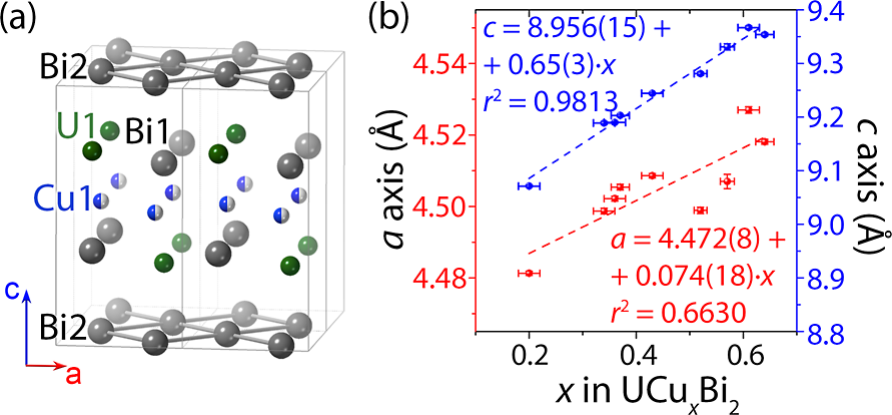
(a) A view of the structure of UCu_*x*_Bi_2_. (b) Dependence of unit cell parameters *a* = *b* and *c* on the Cu content *x* in the UCu_*x*_Bi_2_ single crystals.

Single crystal X-ray diffraction (XRD) showed that all UCu_*x*_Bi_2_ crystals adopt the HfCuSi_2_ structure type with the *P*4/*nmm* space group (Figure 2, Tables S1–S3).^64^ Free refinement of the Cu site occupancy in different UCu_*x*_Bi_2_ crystals showed that the Cu content *x* varies within a range of 0.20–0.64 (Figures S2 and S3). The refinement results are consistent with the energy dispersive spectroscopy (EDS) analysis (Tables S4–S6, Figures S4–S16). The unit cell volume increases simultaneously with the Cu content and varies from 182.17(6) to 190.95(8) Å^3^ for *x* = 0.20 and 0.64, respectively. There are two crystallographically distinct sites of Bi, Bi1, which is surrounded by U and Cu atoms, and Bi2, which form the Bi square nets in the *ab* plane (Figure 2a). The Bi2–Bi2 bond distance in the square nets varies in a relatively narrow range from 3.1688(4) (*x* = 0S.20) to 3.1948(5) Å (*x* = 0.64). The interlayer Bi1 atoms form longer contacts with Bi2 atoms, with interatomic distances ranging from 3.9325(13) to 3.9485(15) Å. The uranium atoms are surrounded by the Bi1 and Bi2 atoms with 3.2523(6)–3.2847(7) and 3.3541(9)–3.3701(10) Å bond distances, respectively. Since the Bi2–Bi2 bond distances within the square nets are shorter than the Bi2–Bi1 and Bi2–U1 bond distances with the interlayer atoms, UCu_*x*_Bi_2_ exhibits a tolerance factor *t* < 0.95 (*t* = *d*_sq_/*d*_nn_, where *d*_sq_ is the distance between the Bi atoms in the square nets, and *d*_nn_ is the distance between a Bi atom in the square net and the closest atom from outside the net), satisfying the criterion for quasi-isolated square nets with Dirac crossings proposed by Schoop et al.^15,31,65^

A closer look at the unit cell parameters’ dependence on the composition shows a much stronger correlation between the *c*-axis length and the Cu content than that of the *a*-axis. Figure 2b shows the dependence of the *c*-axis length on the Cu content *x* with a regression coefficient *r*^2^ of 0.9813. At the same time, the regression coefficient for the *a*-axis dependence is significantly lower at a determined value of 0.6630. Furthermore, the UCu_0.20_Bi_2_ composition can be excluded from the consideration, as it significantly expands the *x*-axis range without as many data points as in the *x* = 0.30–0.65 range, increasing the regression coefficients. Without the UCu_0.20_Bi_2_ data point, the fitting of the *c*-axis vs *x* retains a strong dependence, with *r*^2^ = 0.9765, whereas the *a*-axis dependence shows a drastic drop to *r*^2^ = 0.4348 (Figure S17). This indicates that the almost linear dependence of the volume on the Cu content is primarily driven by *c*-axis elongation. A similar trend has been established for several UCu_*x*_Sb_2_ compositions (*x* = 0.44, 0.64, 0.83, and 1),^53,55,66,67^ and LaMn_*x*_Sb_2_ (*x* = 0.74–0.97).^68^ Since the *a* = *b* lattice parameters, which are responsible for the *Pn*–*Pn* distances in the square nets, exhibit only a marginal change, <0.04 Å/<1%, one can conclude that the band structure arising from them remains the same, while the addition of extra Cu changes the electron count in the material.

To probe the stability range *x* of the UCu_*x*_Bi_2_ phases, we also synthesized a series of powder samples by arc melting the elements and annealing the products at 800 °C for 12 h (Figure S18–S23). The maximum Cu content in the powder samples was around *x* ≈ 0.6, which is in good agreement with the single crystal data. Decreasing the Cu content stepwise from *x* = 0.6 to 0.1 results in phase pure samples (except a slight impurity of unreacted, excess Bi). The stabilization of the UCu_0.1_Bi_2_ composition via direct element combination shows that the stability range can be slightly expanded in the solid state reactions vs flux reactions, which can be employed to stabilize the phases with a desired transition metal content and properties.

### Theoretical Stability of UCu_*x*_Bi_2_ and UCu_*x*_Sb_2_ Phases

According to the six-electron rule,^31^ the A and M sites in the AM_*x*_Bi_2_ (A = Ca, lanthanides, or actinides; M = Li or transition metals) compounds should have a total charge of +4 to charge balance the Bi anions. Only in such a case will the Bi square nets acquire the −1 charge that favors the Fermi level at around the Dirac crossing due to 6p_*x*_ and 6p_*y*_ orbital overlap.^65^ While changing the nature of A and M ions has been widely employed to fulfill the charge balance requirements, controlling the M site deficiency for tuning the Fermi level remains largely underexplored. Our experimental data provides clear evidence of a stability range between 0.20 and 0.64 for the UCu_*x*_Bi_2_ composition, which enables tuning the Fermi level in this composition by varying the Cu content. Since there is increasing evidence that the M site deficiency in the AM_*x*_Pn_2_ compounds can be, to a certain extent, controlled by synthetic means,^68−71^ as well as an increasing role of computational predictions of new compounds,^72^ we performed theoretical ways of predicting the homogeneity range using ab initio calculations.

**Figure 3:**
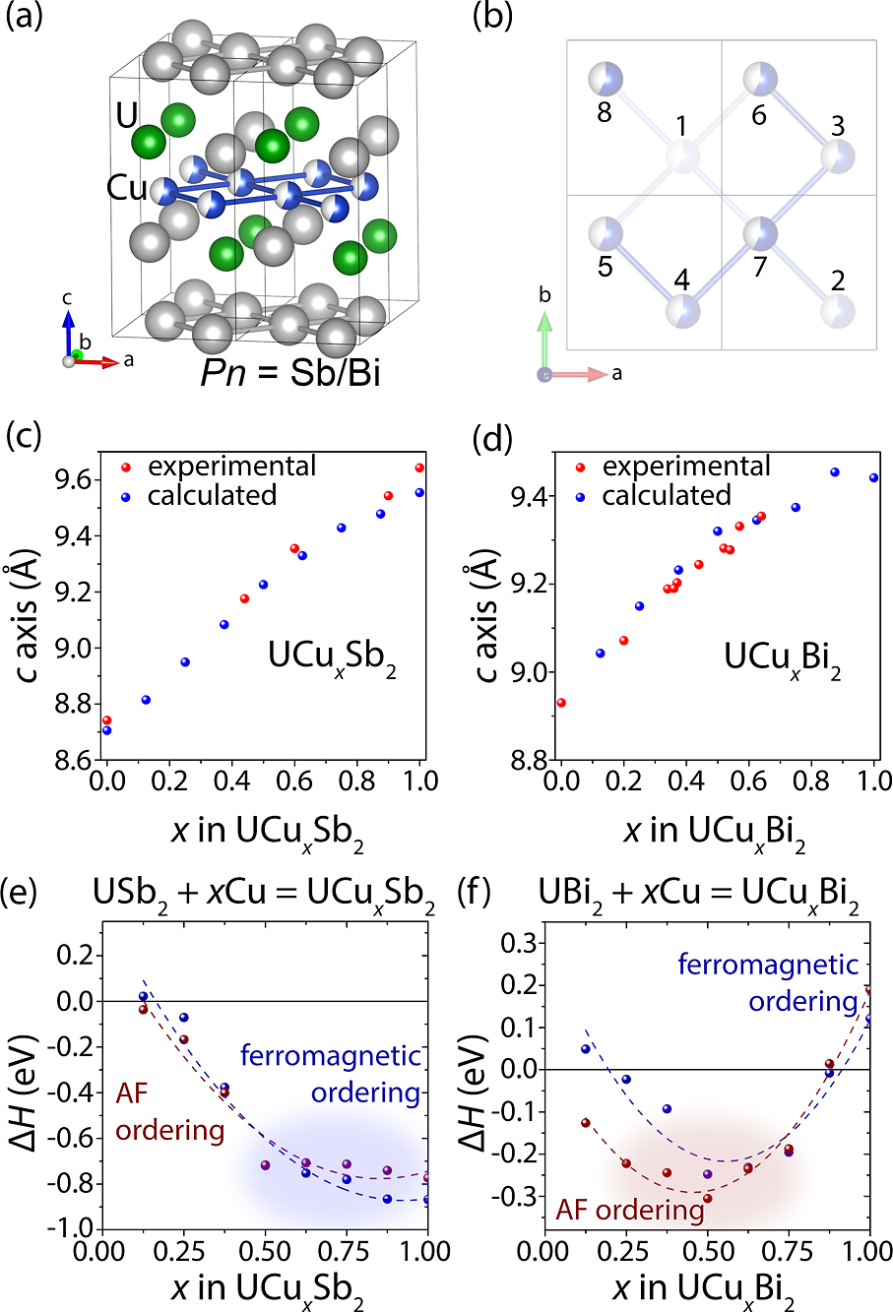
DFT optimization of UCu_*x*_Sb_2_ and UCu_*x*_Bi_2_. (a) 2 × 2 × 1 supercell that was used as a starting model. (b) The order in which Cu atoms were removed from the supercell. Atom labeled with “1” was removed first, yielding a model with a nominal composition of UCu_7/8_Sb_2_ ≡ UCu_0.875_Sb_2_. The last Cu atom corresponding to a UCu_0.125_Sb_2_ composition is atom “8”. (c,d) Plots of calculated and experimental *c*-axis lengths. In both cases, the calculated values show an excellent agreement with the experimental data in the ranges when it is present. (e and f) Calculated enthalpies of reaction corresponding to the formation of (e) UCu_*x*_Sb_2_ and (f) UCu_*x*_Bi_2_ from USb_2_/UBi_2_ and Cu metal for *x* = 0.125 to 1. Blue and red dots correspond to the energies of optimized structures with ferro- and antiferromagnetic ordering. The dashed lines are the guide for the eye that follows the overall trend in the reaction enthalpy change. The most stable calculated compositions correspond to *x* = 0.375–1.000 and 0.250–0.750 for Pn = Sb and Bi, respectively. This data agrees well with experimentally observed homogeneity ranges of 0.40–1.00 (highlighted blue) and 0.20–0.64 (highlighted red) for UCu_*x*_Sb_2_ and UCu_*x*_Bi_2_, respectively.

We chose the UCu_*x*_Bi_2_ and analogous UCu_*x*_Sb_2_ systems for calculating the stability of the compositions with the Cu site deficiency. Previously, a few attempts to mimic such deficiency have been made using DFT calculations. One approach employed generating a supercell along the *c*-axis and removing selected layers of the M atoms completely.^73^ While this approach seems intuitive and agrees with the experimentally observed trends in the change of the average *c*-axis unit cell length, it seems unlikely that the presence of two types of interlayer distances in a crystal would not be observable in single crystal XRD. Since the diffraction data shows only an averaged structure with no indication of domains containing “unstuffed” UBi_2_, we attempted to mimic the site deficiency in UCu_*x*_Bi_2_ and UCu_*x*_Sb_2_ by gradually removing Cu atoms from the same Cu layer in a 2 × 2 × 1 supercell (Figure 3a). Such a supercell contains eight Cu sites, offering eight possible compositions from UCu_1/8_Pn_2_ with only one occupied Cu site to fully occupied UCu_8/8_Pn_2_ (Pn = Sb or Bi), with either ferro- or antiferromagnetic ordering of U moments.

The optimized unit cell parameters agree well with the experimental data (Figure 3c,d, Tables S7–S22). We found that the calculated *c*-axis lattice parameter in UCu_*x*_Sb_2_ varies almost linearly with the composition, from 8.78 Å for *x* = 0.125 to 9.48 Å for *x* = 1.0. This wide range of the calculated *c* parameters agrees with the experimental values of *c* = 9.18 Å for *x* = 0.44 to 9.64 Å for *x* = 1.0 (Figure 3c). Similarly, the UCu_*x*_Bi_2_ optimized geometries agree well with the experimental data within the stability range. The crystal with the maximum experimental Cu content, UCu_0.64_Bi_2_, has a *c* lattice parameter of 9.35 Å that matches *c* = 9.35 Å for the optimized UCu_0.625_Bi_2_ structure. Above *x* = 0.625, the *c* lattice parameter shows rapid growth and eventually saturates when *x* approaches 1.0. While this anomaly violates the expected Vegard’s law trend, this is likely an artifact of optimizing a composition that is unstable both from experimental and computational (vide infra) points of view. Overall, the optimized structures demonstrate that the 2 × 2 × 1 supercell approach agrees well with the experiments. This allowed us to study the applicability of the computational approach further to predict the stabilities of these phases.

We used the DFT formation energies to calculate the enthalpies of U*Pn*_2_ + *x*/8Cu = UCu_*x*/8_*Pn*_2_ reactions, Δ*H*_r_, and probe this computational approach for predicting the stability of HfCuSi_2_-type compounds (Figure 3e and f). In both the UCu_*x*_Sb_2_ and UCu_*x*_Bi_2_ series, the calculations suggest that stability regions depend on the Cu content *x*. In the UCu_*x*_Sb_2_ series (Figure 3e), Δ*H*_r_ values show that the compounds with *x* < 0.375 (Δ*H*_r_(*x* < 0.375) is −0.4 eV or higher) are significantly less stable than the compounds with *x* > 0.375 (Δ*H*_r_(*x* > 0.375) is less than −0.6 eV). This trend is in good agreement with the reported experimental data, as there are several UCu_*x*_Sb_2_ compositions reported with *x* varying from 0.44 to 1.0.^54,55,66,67^ Moreover, the calculations confirm the ferromagnetic ordering in the *x* > 0.4 range, where it is slightly more energetically favorable over the antiferromagnetic ordering. Interestingly, the calculations indicate a preferred antiferromagnetic ordering for the *x* < 0.4 compositions, although they are less likely to be synthesized due to the higher enthalpies of reactions. The Bi analog series, UCu_*x*_Bi_2_, similarly agrees with the experiment. The calculated stability range varies from *x* = 0.2 to 0.75 (vs experimental 0.20 to 0.64), in which, as was predicted computationally and demonstrated experimentally, an antiferromagnetic ground state is favored. Overall, these two examples demonstrate that calculations based on a 2 × 2 × 1 supercell hold great promise for the prediction of the homogeneity ranges in HfCuSi_2_-type AM_*x*_Pn_2_ compounds, enabling a fast screening of the phases that can satisfy the six-electron rule.

### Magnetism of UCu_*x*_Bi_2_ Powder Samples

**Figure 4:**
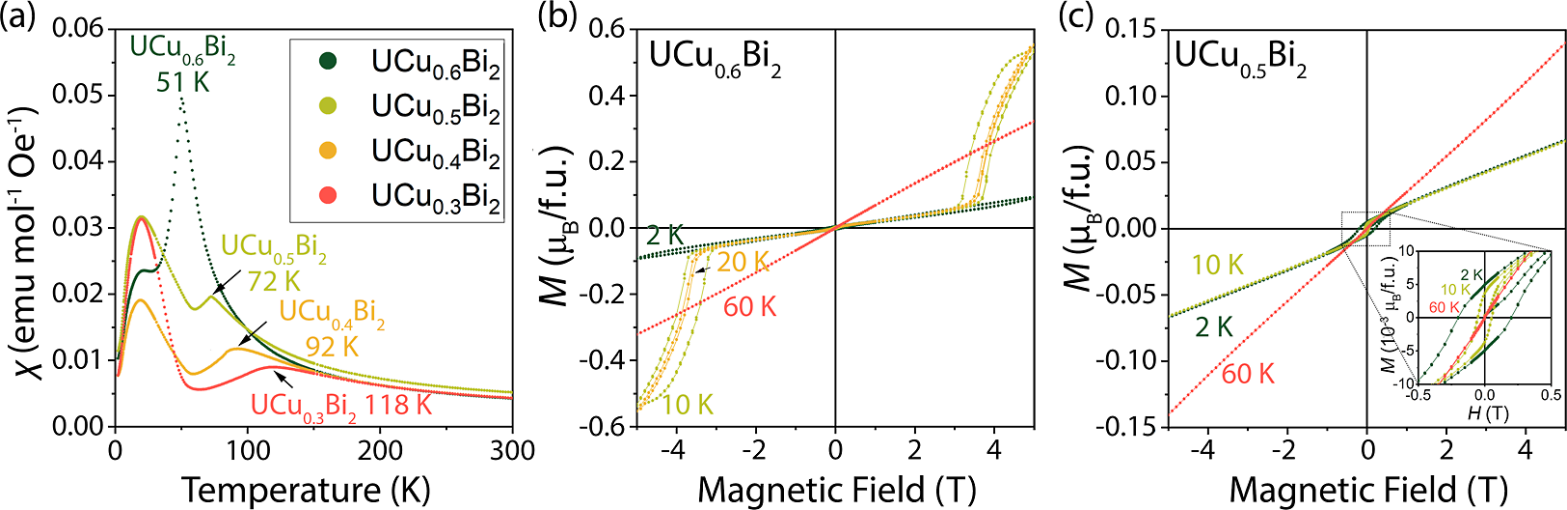
Magnetic properties of powder UCu_*x*_Bi_2_ (*x* = 0.3, 0.4, 0.5, and 0.6) samples. (a) Magnetic susceptibility vs temperature (*H* = 0.1 T) plots show gradually changing Néel temperatures from 118 to 51 K as Cu content increases. The peak around 20 K is likely from an unknown impurity phase. (b) The magnetization vs field plot of the UCu_0.6_Bi_2_ sample shows a metamagnetic transition and hysteresis loop in quadrants I and III. (c) Magnetization vs field plot for a UCu_0.5_Bi_2_ sample shows a different behavior with a weak hysteresis. Since single crystal data does not exhibit such hysteresis, this effect is likely due to an impurity with a ferromagnetic component.

Since the amount of incorporated Cu can affect the magnetic properties of the resulting UCu_*x*_Bi_2_ phases, we collected magnetic susceptibility data for the samples with *x* = 0.3, 0.4, 0.5, and 0.6 (Figure 4). Since the U–U interatomic distances exceed the Hill limit and vary between 4.4813(7) Å for *x* = 0.2 and 4.5181(8) Å for *x* = 0.64, we expected magnetic ordering.^74^ The temperature dependence of the magnetic susceptibility indicates apparent antiferromagnetic transitions, with the Néel temperatures changing linearly from 118 K for UCu_0.3_Bi_2_ to 51 K for UCu_0.6_Bi_2_ (Figures S24–S27). Linear fitting of the Néel temperature vs Cu content plot results in a y-intercept of 183 K (Figure S28), which agrees well with the reported experimental values of the Néel temperature for UBi_2_, 180.8–183 K.^75−77^ Inverse susceptibility plots for the *x* = 0.3 and 0.4 samples are linear in the region >150 K, which can be fitted by the Curie–Weiss law. Both fittings show consistent magnetic moments of 3.30 and 3.31 μ_B_ per formula unit, respectively. Assuming a +1 oxidation state for Cu, one would expect a magnetic moment of 3.62 μ_B_/U^3+^ or 3.58 μ_B_/U^4+^, slightly higher than the experimental values. Similar reduced values have been observed for other U-containing HfCuSi_2_-type phases and are likely due to crystalline electric field (CEF) effects.^52,57^ Since the magnetic moments of U^3+^ and U^4+^ are very close to each other, magnetic data does not allow us to assign the U-oxidation state unambiguously. The Curie–Weiss temperatures of −21.7 and −17.7 K correspond to the *x* = 0.3 and 0.4 samples, respectively. These values decrease simultaneously with the Néel temperatures, 118 and 92 K, and indicate weaker antiferromagnetic interactions as the Cu content increases, likely due to further separation of uranium atoms from each other.

The samples with higher Cu content, UCu_0.5_Bi_2_ and UCu_0.6_Bi_2_, exhibit a slight curvature in the inverse susceptibility plots (Figures S24 and S25). Since the single crystalline samples show linear inverse susceptibility vs temperature behavior (see below), the temperature-independent paramagnetic (TIP) component is likely due to metallic impurities rather than inherent to the compounds. Curie–Weiss law fittings result in magnetic moments of 3.25 and 3.10 μ_B_/f.u. for UCu_0.5_Bi_2_ and UCu_0.6_Bi_2_, respectively. An impurity can explain a second transition at ∼ 20 K in all four samples. Since only high-temperature antiferromagnetic transitions exhibit a temperature dependence on the composition, one can surmise that all four samples have a common minor ferromagnetic impurity, which PXRD could not detect (Figures S18–S23). Interestingly, a similar behavior with two magnetic ordering transitions has been reported for a sample with a nominal composition UCuBi_2_. As the unit cell metrics and Néel temperature, 51 K, for the UCu_0.6_Bi_2_ sample are similar to this previously reported phase,^53^ the data suggests that these compounds may be the same compositions. Overall, the change in the Néel temperature as a function of composition demonstrates the magnetic properties’ tunability in this and related systems.

### Magnetic Properties of UCu_*x*_Bi_2_ Single Crystals

**Figure 5:**
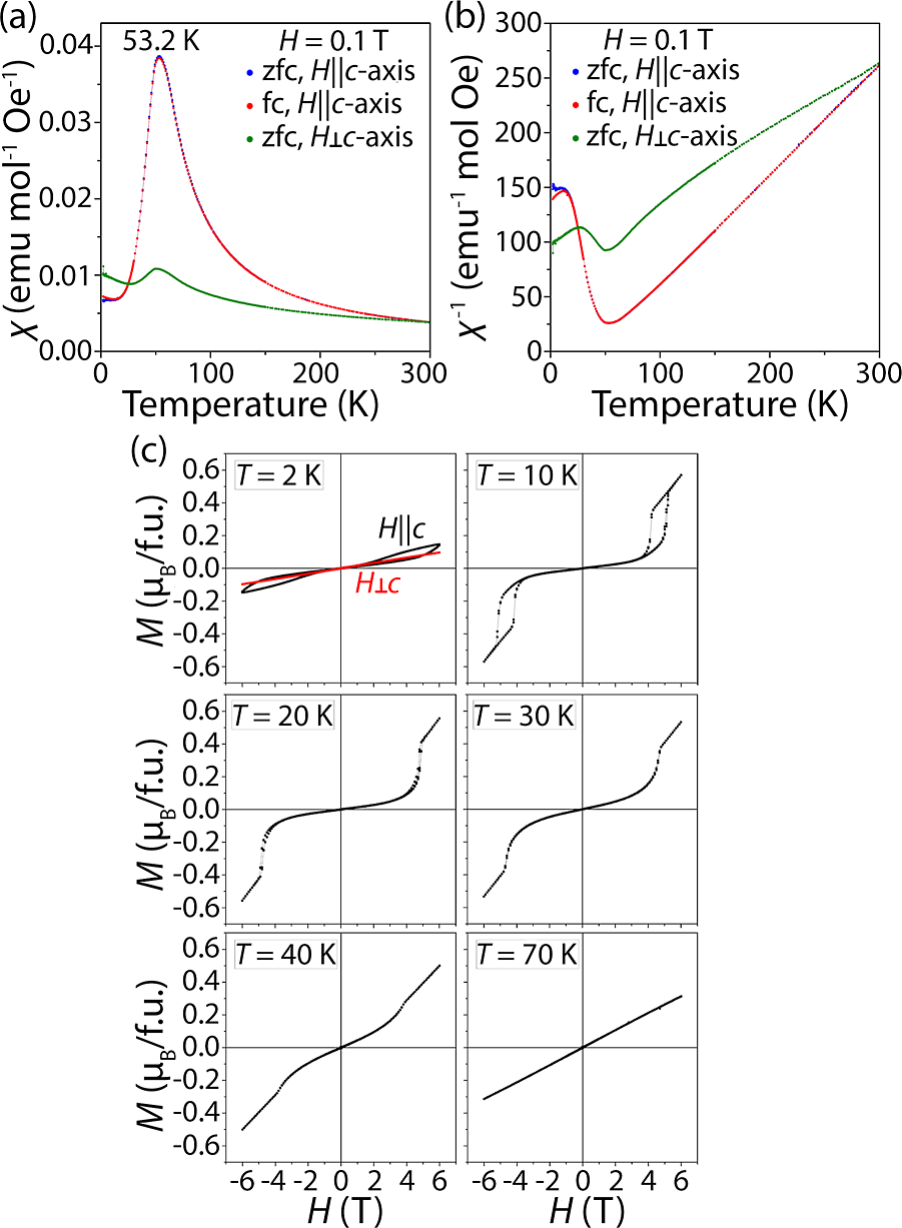
Magnetic properties of single crystals of UCu_0.6_Bi_2_. (a) Magnetic susceptibility and (b) inverse magnetic susceptibility vs temperature plots with the magnetic field applied parallel and perpendicular to the *c*-axis show a strong magnetic anisotropy of the sample. (c) Magnetization vs magnetic field plots show hysteresis effects when the magnetic field is applied along the easy *c*-axis but not perpendicular to it. A first-order metamagnetic transition that is visible on the *T* = 10 K slowly diminishes at higher temperatures.

We characterized the magnetic anisotropy of UCu_0.6_Bi_2_ by collecting the temperature dependent magnetic susceptibility from single crystals. Zero-field (zfc) and field cooled samples with an applied field of 0.1 T show nearly identical behavior (Figure 5), and only the zfc data is discussed below. Magnetic susceptibility χ_||_(*T*) with the magnetic field applied along the *c*-axis of the crystal (perpendicular to the crystal plane) reveals a paramagnetic behavior between 54 and 300 K. The susceptibility peaks at a temperature *T*_N_ of 53.2 K and drops rapidly at lower temperatures, indicating an antiferromagnetic transition. The χ_⊥_(*T*) data shows an overall lower magnetic susceptibility with a maximum of 50.2 K (Figure 5). The crystal shows a significant magnetocrystalline anisotropy with χ_||_(*T*_N_)/χ_⊥_(*T*_N_) = 3.6. We observed a slight sample-to-sample variability in the Néel temperature due to a variation in the Cu content. Interestingly, a different sample with a χ_||_(*T*) susceptibility maximum at 50.2 K, which indicated a slightly higher Cu content, showed a higher magnetic anisotropy value of χ_||_(*T*_N_)/χ_⊥_(*T*_N_) = 11 (Figure S29), which corresponds to the maximum Cu content. Curie–Weiss law fits of the inverse magnetic susceptibility along and perpendicular to the easy *c*-axis, χ^–1^_||_(*T*) and χ^–1^_⊥_(*T*), result in anisotropic magnetic moments of 2.81 and 3.66 μ_B_/f.u., respectively. An average that is calculated as μ_av_ = (μ_||_+2 μ_⊥_)/3 is equal to 3.38 μ_B_ and is in good agreement with the powder data. The Curie–Weiss temperatures from the fits are θ_C–W,||_ = 40.7(2) K and θ_C–W,⊥_ = −141.8(4) K, suggesting the presence of competing ferromagnetic and antiferromagnetic exchange interactions between the U atoms.^55^

Magnetization vs magnetic field plots show a field-induced temperature-dependent metamagnetic-like transition observed when the field is applied parallel to the easy *c*-axis (Figure 5). Unlike the linear dependence of *M*_⊥_(*H*), the *M*_||_(*H*) plot shows a nonwell-resolved hysteresis loop at 2 K and an applied field of 6 T (Figure 5). Magnetization vs temperature data at 10 K allows for better resolution of a metamagnetic transition above 5 T that is accompanied by rapid growth of the magnetization. Upon decreasing the magnetic field, magnetization exhibits a hysteresis with a center at around 4.6 T, which indicates the freezing of coaligned magnetic domains in the crystal. A further temperature increase to 20 K reduces the “coercive field” for the hysteresis loop due to domain thermal motion. Compared to the powder measurement results, the single crystal data shows a more distinct metamagnetic transition that is unaffected by the magnetization perpendicular to the *c*-axis. Since a 6 T magnetic field is insufficient to magnetize the sample fully and only reaches 0.6 μ_B_/U, high-field experiments were employed for a more detailed characterization of the samples.

**Figure 6:**
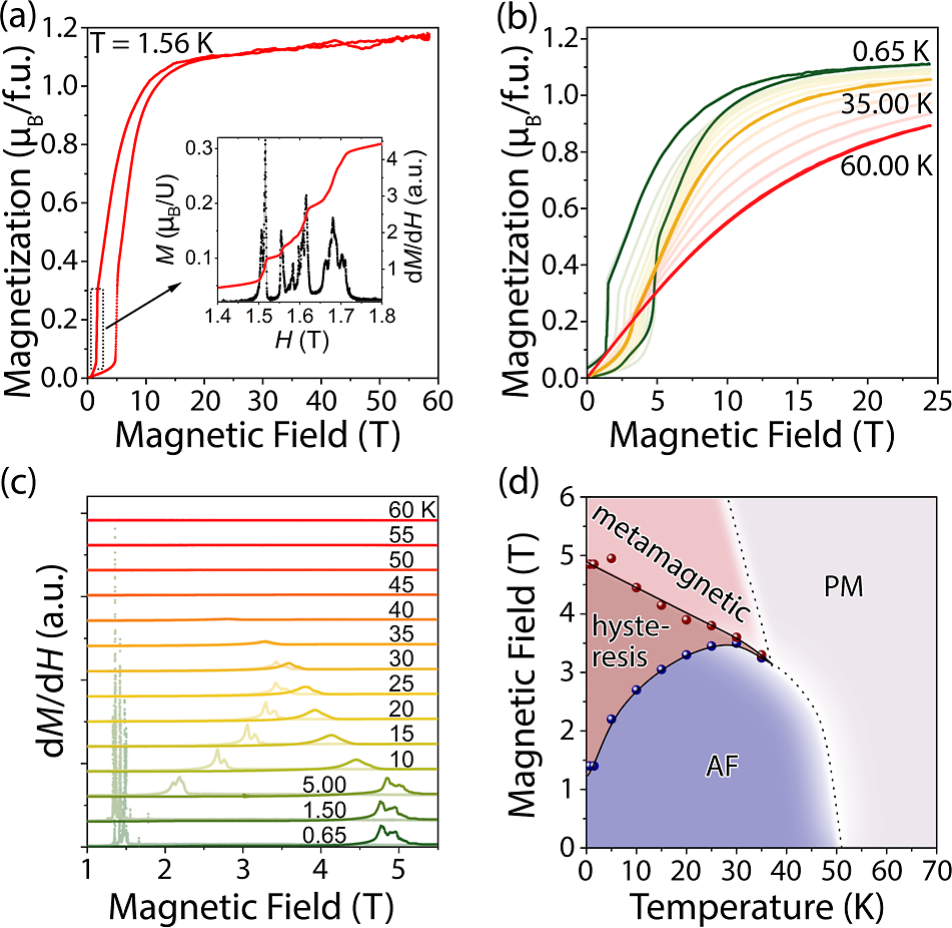
High magnetic field measurements from single crystals of UCu_*x*_Bi_2_. (a) 60 T magnetization vs field plot at 1.56 K shows saturation at around 1.1 μ_B_/U and hysteresis in a metamagnetic transition at around 4 T. The high field magnetization data was scaled using low field measurements. The inset shows a d*M*/d*H* plot at low fields, which reveals domain pinning. (b) A series of *M*v*H* plots show a gradual change of the transition at ∼3 T as a function of temperature. (c) d*M*/d*H* plots for the 25 T data that show the disappearance of the metamagnetic transition and corresponding hysteresis at a temperature above 35 K. (d) Field-temperature phase diagram showing antiferromagnetic (AF), paramagnetic (PM), and metamagnetic regions. The dotted line schematically shows a second-order transition.

We studied the magnetic behavior of UCu_*x*_Bi_2_ (*x* ≈ 0.6) single crystals at a magnetic field up to 60 T (Figures 6 and S30). The highest field *H* = 60 T measurement at 1.56 K indicates that the magnetization nearly saturates to 1.1 μ_B_/U at around a 20 T field, showing only a marginal further increase. At a rising magnetic field, magnetization exhibits a metamagnetic transition at a field of 4.8–5.2 T, which is accompanied by a sharp increase of the magnetization from 0.06 to 0.345 μ_B_/U. Upon decreasing the field, the reverse transition occurs in a range of 1.50–1.72 T with a decrease of the magnetization from 0.293 to 0.058 μ_B_/U. The inset of Figure 6a shows a stepwise demagnetization of the sample in the 1.50–1.72 T range, highlighting domain pinning due to high crystallographic defect concentration in the sample.

A series of variable temperature magnetization vs field measurements with *H*_max_ = 25 T shows a gradual change in magnetization behavior as temperature increases (Figure 6b). At low temperatures, 0.65–30.00 K, the magnetization curves show a sharp metamagnetic transition and a hysteresis between forward and reverse transitions. As the sample temperature reaches 35 K, the hysteresis almost completely disappears, and only a small kink indicating a metamagnetic transition remains. A further temperature increase above 40 K leads to a featureless magnetization with slow saturation at the higher fields. The sharp change of the magnetization at low temperatures and the presence of hysteresis suggest a first-order metamagnetic transition from an antiferromagnetic ground state. A relatively small change in the magnetic moment as a fraction of the saturated moment, *M*_transition_/*M*_sat_ = 0.345/1.1 ≈ 1/3, along with a wide range of slow saturation at the higher field, indicates a spin-flop transition followed by a gradual spin rearrangement. As the temperature increases, the transition becomes less prominent and becomes a second-order transition at around 40 K. Figure 6c illustrates this transition by showing d*M*/d*H* plots as a function of temperature. At low temperatures <1.50 K, magnetization spikes are prominent for forward and reverse transitions. Such behavior indicates a transition of a ferrimagnetically ordered state at low temperatures, gradually shifting toward more paramagnetic-like behavior at the tricritical temperature *T*_tc_. For a two-sublattice antiferromagnet with ferromagnetic intralayer coupling (as determined by neutron diffraction), the tricritical temperature can be calculated using the mean-field formula^78,79^
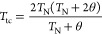
where *T*_N_ and θ are the Néel temperature and Curie–Weiss constant, respectively. Using *T*_N_ of 51 K and θ of 11.4 K from the powder data Curie–Weiss law fit, one can calculate a tricritical point of 40 K, which agrees very well with the experimental data.

Some other uranium compounds have also demonstrated low-temperature metamagnetic transitions from an antiferromagnetically ordered state, but most occur at significantly higher fields. For example, cubic UN exhibits a metamagnetic transition with a critical point at *T*_tc_ = 24 K and *H*_tc_ ≈ 52 T.^80^ Another example is UNi_0.34_Ge_2_, which orders antiferromagnetically at 45.5 K and demonstrates a spin-flip transition when magnetic fields of >4 T are applied along the easy *b*- and *c*-axes.^81^ USb_2_, which can be considered as an unstuffed structural analog of UCu_*x*_Bi_2_, shows a distinct metamagnetic transition at fields as high as 52 T and a tricritical point at ∼145 K.^79^ The higher field that is required for the metamagnetic transition correlates well with the higher Néel temperature of USb_2_, 202.3 K, which indicates stronger antiferromagnetic exchange interactions. Since UCu_*x*_Bi_2_ shows an increasing Néel temperature as Cu content decreases, only the highest Cu content phase, UCu_0.6_Bi_2_, showed a metamagnetic transition at the magnetic fields below 5 T. Therefore, one can expect higher field metamagnetic transitions in phases with lower Cu content due to stronger antiferromagnetic coupling between the uranium layers. However, drawing an analogy between USb_2_ and UCu_0.6_Bi_2_ was incomplete without determining the latter’s magnetic structure.

### Single-Crystal Neutron Diffraction

**Figure 7:**
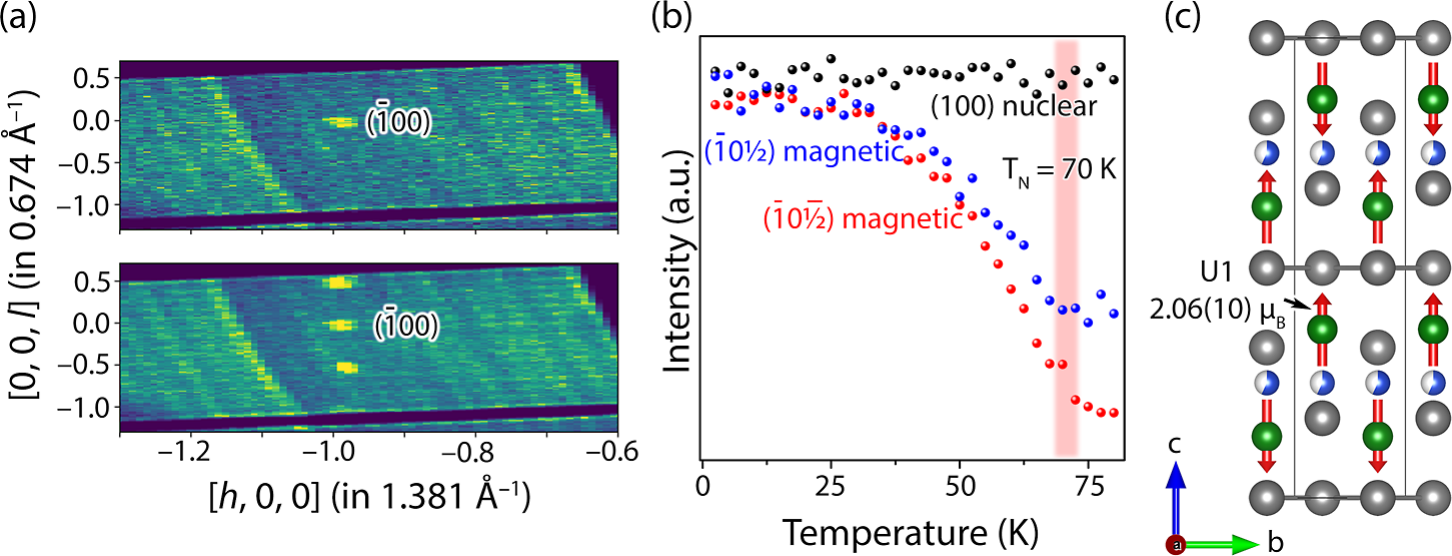
Neutron diffraction data from a UCu_*x*_Bi_2_ crystal (*x* ≈ 0.5). (a) Neutron diffraction frames showing nuclear  and magnetic  and  reflections at 80 and 2 K. (b) shows the dependence of the nuclear and magnetic reflections on the temperature at *T* = 2 K. (c) shows the magnetic structure of UCu_0.5_Bi_2_ at *T* = 10 K with a propagation vector *k* = (0, 0, 0.5).

We solved the magnetic structure of UCu_0.6_Bi_2_ by using single-crystal neutron diffraction data collected at 10 K on the HB-3A beamline at ORNL.^82−84^ The crystal showed distinct magnetic diffraction peaks with a propagation vector *k* = (0, 0, 0.5), indicating doubling of the unit cell along *c* upon magnetic ordering (Figure 7a). The intensity of magnetic peaks collected as a function of temperature (Figure 7b) shows that the Néel temperature of the sample is around 70 K. Such a high T_N_ indicates that the selected crystal’s composition is actually closer to UCu_0.5_Bi_2_. The best refinement of nuclear and magnetic peaks at 2 K was achieved when using a *P*_*c*_4/*ncc* (#130.432) magnetic space group,^85^ which is the same as in the USb_2_ compound.^86^Figure 7c shows a refined magnetic structure with an antiferromagnetic ordering of the magnetic moments along the *c*-axis, which agrees with the anisotropic magnetic susceptibility data. The magnetic structure of UCu_0.6_Bi_2_ can be understood if considered as U–Cu–U slabs separated by Bi square nets. In the U–Cu–U slabs, the U atoms with refined magnetic moments of 2.06(10) μ_B_ are separated by the partially occupied Cu site with a shorter U–U distance, 4.3 Å, than the distance between the slabs, 5.9 Å. The ordering within the slabs is antiferromagnetic while neighboring U atoms from the successive slabs are ordered ferromagnetically to each other (Figure 7c). Such magnetic ordering differs from the UBi_2_ magnetic structure, where the U magnetic moments are aligned antiferromagnetically within and between the slabs.

### Theoretical Calculations of Magnetic Exchange Interactions

**Figure 8:**
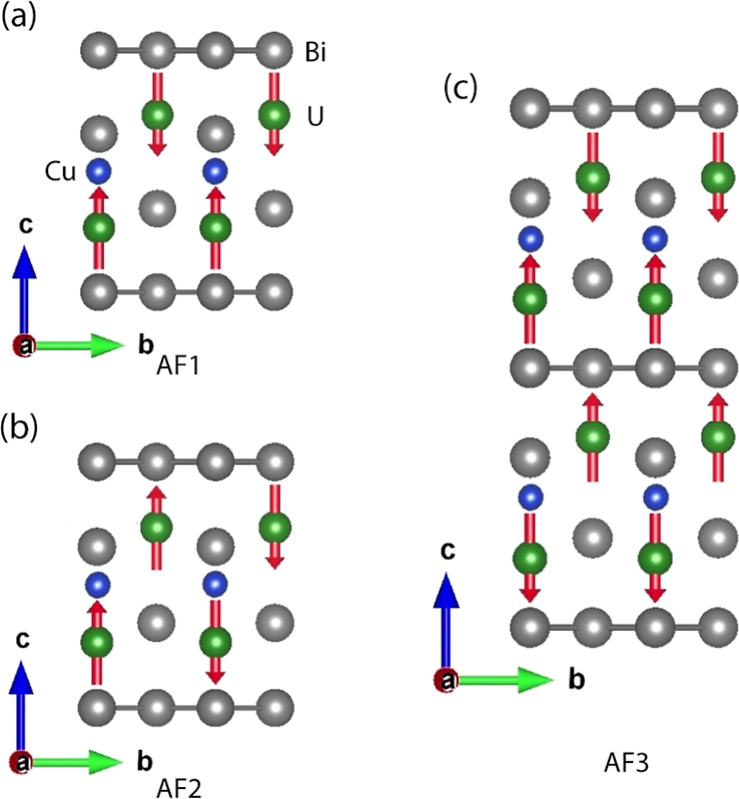
Calculated antiferromagnetically ordered structures of UCu_0.5_Bi_2_.

To better understand the magnetic behavior depicted in the previous sections, we have conducted first-principles calculations of the magnetism in this set of materials within the GGA + SO approximation as implemented in the all-electron linearized augmented planewave code WIEN2K.^87^ For simplicity, we have taken a fully ordered unit cell of stoichiometry UCu_0.5_Bi_2_ as detailed in Figure 8, with planar and *c*-axis lattice parameters, respectively, of 4.4989 and 18.563 Å, in good correspondence with the experimental values for this composition. Within this structural model, we have relaxed internal coordinates using a ferromagnetic configuration of U moments to allow for magnetoelastic effects.^88−95^ As has been shown previously, such an approach is generally more accurate when describing magnetic behavior, particularly for heavy element systems. We did not enable spin–orbit coupling for the geometry optimization, and an R*K*_max_ of 9.0 was employed throughout the calculations, which is the product of the smallest muffin-tin sphere and the largest plane-wave expansion wavevector.

In addition to the ferromagnetic state, we performed calculations on several antiferromagnetic states (Figure 8), including one (AF1) with neighboring U planes antialigned, one with planar U–U nearest neighbors antialigned (AF2), and the experimentally observed groundstate (AF3). In all studied magnetic states, U exhibits a significant magnetism with U spin moments of approximately 2 μ_B_. All models show substantial magnetic anisotropies, ranging from 8 to 34 meV/U, which is indicative of the extremely strong spin–orbit coupling on the U atoms. The calculations demonstrate that UCu_0.5_Bi_2_ belongs to a class of magnetic material in which the magnetic anisotropy rivals or even outstrips the exchange interaction in magnitude. The AF2 state falls 25 meV/U higher than the AF1 state, consistent with the experimental finding of ferromagnetic exchange coupling within the planes, and the ferromagnetic state FM is 12 meV/U higher than AF1. Given that this state energetics describes the nearest and next-nearest neighbor interactions, one can make an estimate of the ordering point as 1/3 of these energetic differences,^96−100^ or between 46 and 97 K, which brackets the experimental value. The theoretical ground state AF1 exhibits a U spin magnetic moment of 2.04 μ_B_, which is very close to the experimentally observed 2.06 μ_B_. Notable, in this AF1 state, the U moments prefer a *c*-axis orientation, with magnetic anisotropies relative to the plane of 8 to 34 meV per U, depending on which of the planar directions in the effectively orthorhombic unit cell is chosen.

Unexpectedly, within these calculations, the experimental AF3 groundstate falls 8 meV above the AF1 state, although, within the AF3 calculation manifold, we do find the same *c*-axis U orientation as in the experimental groundstate. This difference may arise due to a number of factors, including the simplified structural model chosen, not including correlative (i.e., GGA + *U* was not employed) effects, as well as the general difficulty of first-principles approaches in describing actinide materials (e.g., ref (101) provide another example). It is also noteworthy, that the calculated groundstate agrees well with the experimental one for UBi_2_. Given the Cu site deficiency and a linear change of the Néel temperature, one can surmise that the groundstate magnetic ordering switches from AF3 to AF1 at some Cu content. Therefore, the theoretical findings of planar exchange interactions and *c*-axis moment orientation and generally antiferromagnetic behavior with a reasonable Néel point are in good agreement with the experiment. However, more advanced theoretical approaches, such as dynamic mean-field theory (DMFT), may ultimately be required to attain a full description of the magnetism in this and like systems.

**Figure 9:**
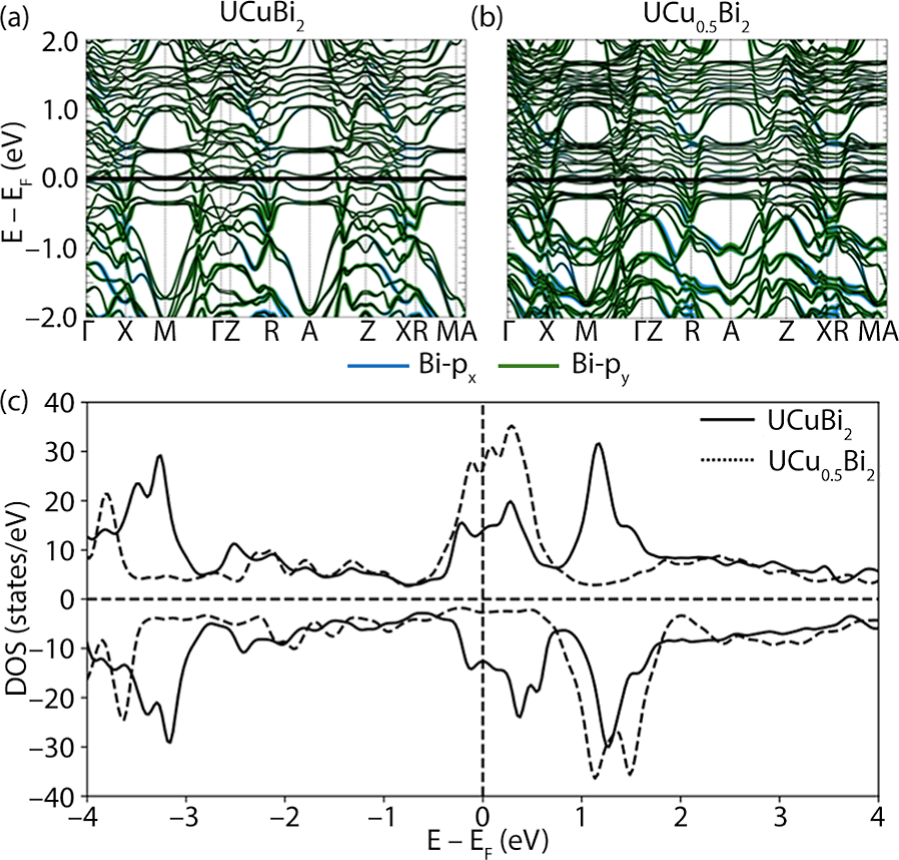
Calculated antiferromagnetic (AF3) band structures of (a) UCuBi_2_ and (b) UCu_0.5_Bi_2_, along with (c) the total calculated densities-of-states for both compositions.

Figure 9 shows calculated band structures for UCu_0.5_Bi_2_ and hypothetical UCuBi_2_, in which the partially occupied Cu positions are set to full occupancy with the unit cell parameters extrapolated based on equations in Figure 2b. Additionally, we include calculated densities-of-states for these compositions. All calculations are performed within the experimental AF3 state for simplicity. The full band structure of stoichiometric UCuBi_2_ is significantly simpler than that of UCu_0.5_Bi_2_, as the use of an effectively lower-symmetry cell in the latter calculation introduces additional bands (this is most evident near the A and M points). Bi orbital projections show that the main contribution to the disperse bands below the Fermi level is made by Bi 6p_*x*_ and 6p_*y*_ orbitals. The nearly flat bands at the Fermi level mostly have U 5f character (not shown for simplicity), which agrees with our simplified treatment of this material (i.e., no GGA + *U*). One can also note numerous crossings of the bands with Bi p orbital character within one eV below the Fermi level, as well as avoided crossings. Additionally, in the fully occupied UCuBi_2_ band structure, certain bands appear to fall farther below the Fermi level than in the partially occupied UCu_0.5_Bi_2_ structure, consistent with the additional Cu atoms adding electrons to the system (this can again be seen most easily near the A and M points).

For additional characterization of the Fermi level shifting as a function of composition, we also plotted the calculated electronic densities-of-states (Figure 9c). There is an evident shift of the predominant spin-up peak from a position bracketing the Fermi level to a narrower peak slightly over 1 eV above the Fermi level, while the effects in the spin-down channel are yet more complicated, with the ∼1 eV spin splitting in the partially occupied UCu_0.5_Bi_2_ nearly absent in UCuBi_2,_ which overall exhibits a DOS much more nearly symmetric between spin-up and spin-down projections.

In general, these results appear rather far from a “rigid band” shift, which is generally not surprising in view of the complexities typically applicable to actinide materials, complexities here heightened by both the partial Cu occupancy and the additional large spin–orbit energy scale applicable to Bi. Despite all these effects, the magnetic interactions and structures appear to be reasonably well-depicted by the first-principles calculations, and we await further investigations of this system.

## Conclusions

In this report, we have synthesized and studied the properties of a series of new site-deficient HfCuSi_2_-type compounds UCu_*x*_Bi_2_. Unlike in the previously reported stoichiometric 1:1:2 phase, the Cu content *x* in flux-grown crystals does not exceed ∼0.64, even with a substantial excess of Cu in the starting reaction mixture. Varying the Cu content in the reactions offers control over Cu incorporation into the final products, revealing a series of UCu_*x*_Bi_2_ compositions with *x* changing in a wide range between 0.2 and 0.64. Structural studies show a direct correlation between the unit cell *c*-axis and Cu content. Magnetic measurements from single crystals show strong magnetocrystalline anisotropy with an easy *c*-axis and antiferromagnetic ordering at low temperatures. A high Cu content in UCu_0.64_Bi_2_ weakens the magnetic exchange along the *c*-axis sufficiently enough to enable a first-order field-polarized spin-flop transition at temperatures below 35 K. The magnetization data suggests a tricritical point in a temperature range between 35 and 40 K under an applied field of 3 T. single crystal neutron diffraction data confirmed an antiferromagnetic ground state in a UCu_*x*_Bi_2_ (*x* ≈ 0.5) single crystal. Overall, the magnetic measurements show that magnetic coupling between the layers of magnetic U atoms and, therefore, the Néel temperatures in these phases directly depend on the Cu content, offering it as a means to compositionally tune the magnetic properties.

Although the resulting series demonstrates a site deficiency that violates the desirable electron count in an idealized U^3+^Cu^+^Bi^3–^(Bi^–^)_sq_ topological phase, this work enables an alternative pathway to achieve a topological state in the HfCuSi_2_-type compounds. For example, the Fermi level in numerous A^3+^M^2+^_*x*_Pn_2_ (A = lanthanide or actinide, M = transition metal, Pn = pnictides) can be tuned to the Dirac crossing in its electronic band structure by synthetic control over M^2+^ site deficiency. Our DFT calculations on a series of UCu_*x*_Sb_2_ and UCu_*x*_Bi_2_ compositions (*x* = 0.125–1) show that the stability ranges of site deficient phases can be successfully predicted computationally. Although DFT calculations show that the band structures of idealized UCuBi_2_ and UCu_0.5_Bi_2_ compounds show symmetry-related differences, the overall observed shift in the Fermi level confirms the dependence of the electron count on the transition metal site occupancy. These observations open a pathway to search for the potential topological phases in site deficient HfCuSi_2_ systems.

## Experimental Methods

### Caution

Although the uranium precursor used in this synthesis contains depleted uranium, proper procedures for handling radioactive materials must be observed. All handling of radioactive materials was performed in laboratories specially designated for studying radioactive actinide materials.

### Caution

Uranium metal, some target phases, and side products, such as UBi_2_, are highly pyrophoric and prone to spontaneous ignition in air. Small quantities of the samples should be handled at one time in an inert atmosphere.

### Reagents

Bi (Unique Metals, 99.99%), Cu (Bean Town Chemical, 99.5%), HNO_3_ (VWR Chemicals, 68–70%), and acetone (Fisher Chemical, 99.5%) were used as received. U sheet (Manufacturing Sciences Corporation, >99%) was cleaned of the oxide layer using concentrated nitric acid (HNO_3_) followed by an acetone rinse and cut into smaller pieces before use in a reaction.

### Synthesis

Single crystals of UCu_*x*_Bi_2_ were grown using the self-flux method, with excess bismuth as the flux. Uranium, copper, and bismuth were combined in molar ratios from 1:0.5:19 to 1:4:19 (Table S23) and loaded into an alumina crucible (9 and 6 mm outer and inner diameters, respectively). The crucible was loaded into a quartz tube (12 and 10.5 mm outer and inner diameter, 20 cm long) and covered by a piece of silica wool for product filtration. The silica tube was vacuum-sealed and placed into a programmable box furnace. The sealed reaction was heated to 950 °C over 2 h, where it dwelled for 2 h before being cooled to 480 °C over 3 h. Upon cooling to 480 °C, the reaction was immediately centrifuged. Upon opening the reaction vessels in an argon-filled glovebox, the product was manually recovered. Since the Cu site deficiency varies slightly in the crystals of the same batch, the compositions of the single crystals for property measurements were measured using SC XRD and/or SEM EDS. To confirm the homogeneity of the single crystals, the EDS data were collected three times from three different areas of the crystal, showing consistent compositions within the measurement error (Table S5).

Powder samples were synthesized via arc melting and annealing. The reagents were filed into a powdered form in an argon-filled glovebox, where they were mixed in exact stoichiometric ratios for the desired product, with a 2% excess of Bi to account for its volatility under high temperatures. Each mixture was then placed into a die and pressed into a pellet using a hand press inside the glovebox. These pellets were then individually removed and immediately placed into the chamber of the arc melter, where they were vacuumed down and placed under an argon atmosphere. Each pellet was arc melted two times, once on each side, to ensure thorough mixing with the sample flipped and placed in a new, clean well between each melt. The weight loss in a typical arc melting reaction did not exceed 1%. Each resulting sample was immediately transferred to the glovebox upon its removal from the arc melter, where it was encased in tantalum foil and placed in a silica tube. The silica tube was vacuum sealed and placed into a programmable box furnace, where it was annealed at 800 °C for 12 h. The annealed samples were transferred back to the glovebox, where they were opened, and the products were manually retrieved from the tantalum foil. The peak positions in PXRD of the powder samples matched the calculated ones form the cif files, confirming their composition.

### Magnetism

Magnetic property measurements were performed using a Quantum Design MPMS 3 SQUID magnetometer. Zero-field-cooled (ZFC) magnetic susceptibility measurements from powder samples were performed from 2 to 300 K in an applied field of 0.1 T. The raw powder data was corrected for radial offset and sample shape effects according to the method described by Morrison and zur Loye.^102^ The high-field (up to 60 T) data were taken at the pulsed-field facility of the National High Magnetic Field Laboratory (NHMFL, Los Alamos). Single crystal data was collected in the same temperature range from a single crystal that was oriented and glued to a quartz paddle using GE varnish. Crystal orientation was established using a PXRD scan of a plate-like single crystal on a zero-background Si slide.

### Calculations

First-principles calculations were performed using density functional theory (DFT) with the Vienna Ab-initio Package (VASP) planewave code,^103,104^ generalized gradient approximation of Perdew, Burke, and Ernzerhof (PBE),^105^ and projector augmented wave (PAW) method.^106,107^ The ground state geometries at 0 K were optimized by relaxing the cell volume, atomic positions, and cell symmetry until the maximum force on each atom is less than 0.001 eV/Å. Spin-polarized calculations were performed, with 520 eV cutoff energy for the plane wave basis set, 10^–6^ eV energy convergence criteria, and 6 × 6 × 5 *k*-point meshes for the quadrupled unit cells generated by VASPKIT package. The *k*-paths for band structure calculations were generated using VASPKIT package.^108^

### Neutron Diffraction

The magnetic structure was determined using single crystal neutron diffraction experiments on the HB-3A DEMAND at the High Flux Isotope Reactor at Oak Ridge National Laboratory.^83^ The sample of about 3 × 3 × 0.5 mm^3^ size was polished to remove trace surface contaminants before loading it on an Al pin. The sample was measured with the two-axis mode down to 2.0 K using a cryomagnet and a wavelength of 1.542 Å from a bent Si-220 monochromator.^82^ The measurement was performed with an applied magnetic field from 0 to 6 T parallel to the [0 0 1] direction of the crystal. The Bilbao Crystallography Server was used for the magnetic symmetry analysis, and Fullprof software for the magnetic structure refinement.^109,110^ Final refinement *R*_F_^2^ factors are 2.19 (nuclear reflections), 28.33 (magnetic reflections), and 17.46 (combined nuclear and magnetic reflections).

## Supporting Information Available

Additional experimental details, selected crystallographic data and XRD experiment parameters, atomic displacement parameters, PXRD patterns, EDS results and SEM images, atomic coordinates after structure relaxation, and additional magnetic data (Tables S1–S19, Figures S1–S30) (PDF)
