# Supplementary material for: Magnetic Properties Tuning via Broad Range Site Deficiency in Square Net Material UCuxBi2
Source: J Am Chem Soc. 2025 Apr 22;147(18):15157–69. doi: 10.1021/jacs.4c18438 (PMC12063058; doi:10.1021/jacs.4c18438)
Supplement: Supplementary file 1 — ja4c18438_si_001.pdf [file ja4c18438_si_001.pdf]

Supporting Information for

Magnetic Properties Tuning via Broad Range Site Deficiency in Square Net Material UCu_xBi_2

Hope A. Long,[a] Daniel Duong,[b] Joanna Blawat,[c] Gregory Morrison,[d] Yan Wu,[e] Huibo Cao,[e] Nabaraj Pokhrel,[f] David Parker,[f] John Singleton,[c] Rongying Jin,[b] Vladislav V. Klepov*[a]

[a] Department of Chemistry, University of Georgia, Athens, Georgia, 30602, United States

[b] SmartState Center for Experimental Nanoscale Physics, Department of Physics and Astronomy, University of South Carolina, Columbia, South Carolina 29208, United States

[c] NHMFL, Los Alamos National Laboratory, MS E536, Los Alamos, New Mexico 87545, United States

[d] Center for Hierarchical Waste Form Materials, and Department of Chemistry and Biochemistry, University of South Carolina, Columbia, South Carolina 29208, United States

[e] Neutron Scattering Division, [f] Materials Science and Technology Division, Oak Ridge National Laboratory, Oak Ridge, Tennessee, 37831, United States

Table of contents

**Additional experimental details**	S3
**Figure S1.** Optical images of single crystals	S3
**Figures S2 and S3.** Thermal ellipsoids	S4
**Table S1-S3.** Crystallographic parameters	S6
**Tables S4-S6 and Figures S4-S15.** EDS data	S9
**Figure S17.** Unit cell parameters fit	S17
**Figures S16-S22.** PXRD patterns	S18
**Tables S7-S22**. DFT optimized structure parameters of UCu_xBi_2 phases	S21
**Figures S23-S27**. Magnetic properties of powder samples of UCu_xBi_2 (x = 0.3, 0,4, 0.5, 0.6)	S37
**Table S23.** Flux reaction summary table	S38
**Table S24.** Arc melted reaction summary table	S38
**Figures S28-S30**. Magnetic properties of a single crystal of $UCu_{0.6}Bi_2$	S41
**Figure S31.** Precession images of a single crystal of $UCu_{0.6}Bi_2$	S45

Powder X-ray diffraction. Powder X-ray diffraction (PXRD) data for phase identification and phase purity confirmation were collected on polycrystalline samples. Data were collected on a Bruker D2 PHASER diffractometer utilizing Cu Kα radiation. The data were collected over the range from 10° to 65° 2θ with a step size of 0.02°. The PXRD patterns are shown on Figures S2–7.

Crystal Structure. Single-crystal X-ray diffraction data was collected at 300(2) K on a Bruker D8 QUEST diffractometer equipped with an Incoatec IμS 3.0 microfocus radiation source (Mo Kα, λ = 0.71073 Å) and a PHOTON II area detector. The crystals were mounted on a microloop using immersion oil. The raw data reduction and absorption corrections were performed using APEX3 v2019-1.0 and SADABS programs.[1,2] Initial structure solutions were obtained with SHELXS-2017 using direct methods and Olex2 GUI.[3] Full matrix least-squares refinements against F^2 were performed with SHELXL software.[4] The crystallographic data and results of the diffraction experiments are summarized in Tables S1–S3.

Scanning Electron Microscopy (SEM). SEM images were acquired using a Thermo Fisher Teneo FE-SEM operated at 20 kV with a CBS detector.

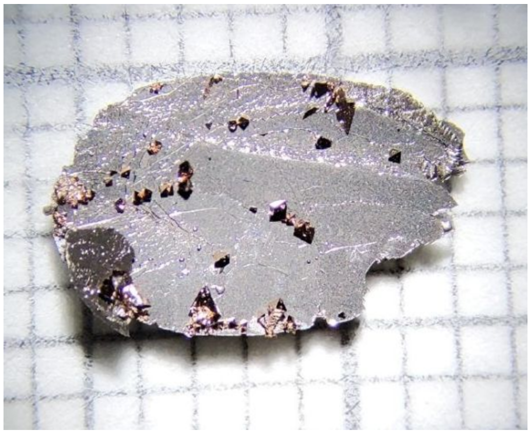

(a)

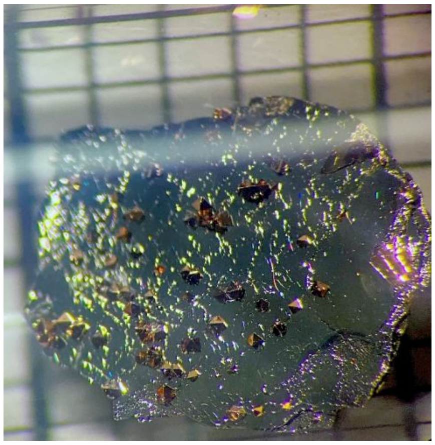

(b)

Figure S1. Optical images of UCu_xBi_2 single crystals obtained from (a) a 1:3:19 ratio of U:Cu:Bi in alumina crucibles and (b) from a 1:4:19 ratio. Octahedral red crystals on the surface of UCu_xBi_2 are Cu.

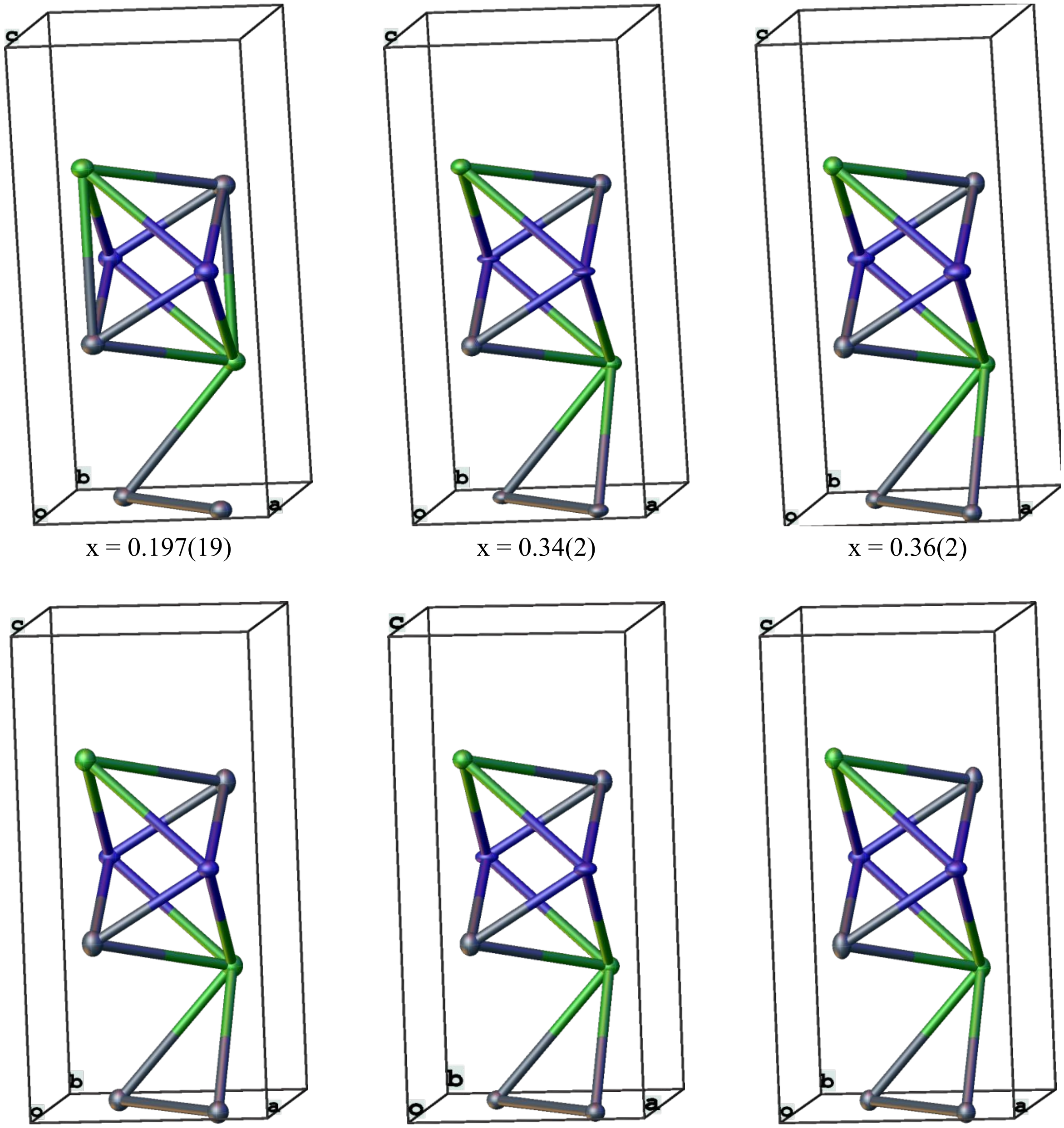

x = 0.197(19) x = 0.34(2) x = 0.36(2)

x = 0.367(17) x = 0.43(2) x = 0.520(12)

Figure S2. A view on the UCu_xBi_2 (x = 0.2 – 0.52) unit cells. The atoms shown as thermal ellipsoids with 50% probability.

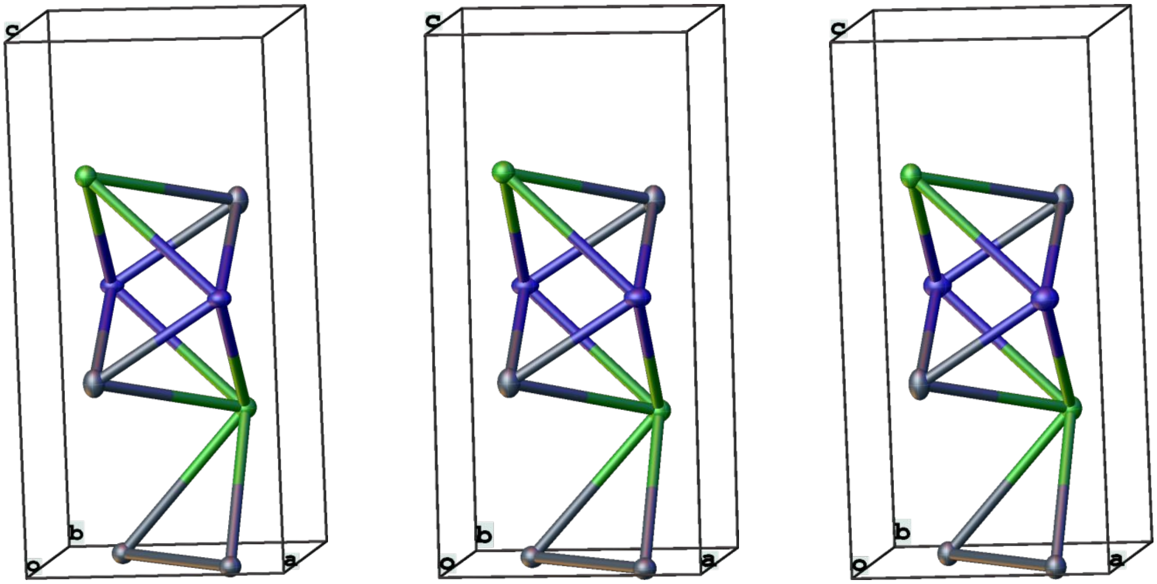

x = 0.570(13) x = 0.611(14) x = 0.636(17)

Figure S3. A view on the UCu_xBi_2 (x = 0.57 – 0.64) unit cells. The atoms are shown as thermal ellipsoids with 50% probability.

Table S1. Crystallographic data for UCu_xBi_2 single crystals (x = 0.2 – 0.64)

x	0.2	0.34	0.36	0.37	0.43	0.52	0.57	0.61	0.64
Formula weight	668.38	677.59	678.86	679.18	683.63	689.03	692.21	694.75	696.34
Temperature/K	300								
Crystal system	tetragonal								
Space group	P4/nmm								
a/Å	4.4813(6)	4.4987(8)	4.5022(7)	4.5054(8)	4.5086(6)	4.4989(9)	4.507(2)	4.5270(9)	4.5181(7)
b/Å	4.4813(6)	4.4987(8)	4.5022(7)	4.5054(8)	4.5086(6)	4.4989(9)	4.507(2)	4.5270(9)	4.5181(7)
c/Å	9.0713(19)	9.189(3)	9.190(2)	9.203(2)	9.2442(18)	9.281(3)	9.331(6)	9.367(3)	9.354(3)
Volume/Å^3	182.17(6)	185.98(8)	186.29(8)	186.80(8)	187.91(6)	187.85(9)	189.6(2)	191.97(10)	190.95(8)
Z	2								
ρcalcg/cm^3	12.185	12.1	12.103	12.075	12.082	12.182	12.127	12.019	12.111
μ/mm^{-1}	141.527	139.437	139.317	138.959	138.526	139.042	138.059	136.54	137.407
F(000)	527	536	537	537	541	546	549	551	553
Crystal size/mm^3	0.04 × 0.04 × 0.01								
Radiation	MoKα (λ = 0.71073)								
2Θ range, °	8.99-54.89	8.87-49.80	4.43-49.97	8.86-49.93	8.82-49.91	8.78-66.13	8.74-49.72	4.35-49.94	4.35-49.87
Index ranges	-5 ≤ h ≤ 4	-4 ≤ h ≤ 5	-5 ≤ h ≤ 5	-5 ≤ h ≤ 5	-5 ≤ h ≤ 5	-6 ≤ h ≤ 5	-5 ≤ h ≤ 5	-4 ≤ h ≤ 5	-4 ≤ h ≤ 5
	-5 ≤ k ≤ 5	-5 ≤ k ≤ 3	-5 ≤ k ≤ 4	-5 ≤ k ≤ 5	-5 ≤ k ≤ 5	-6 ≤ k ≤ 6	5 ≤ k ≤ 5	-5 ≤ k ≤ 5	-4 ≤ k ≤ 5
	-11 ≤ l ≤ 11	-10 ≤ l≤ 10	-10 ≤ l≤ 10	-10 ≤ l≤ 10	-10 ≤ l ≤ 9	-13 ≤ l≤ 14	-11 ≤ l≤ 11	-11 ≤ l ≤ 10	-11 ≤ l≤ 10
Reflections	1384	1367	1424	1415	1344	2280	1499	1518	1394
Independent reflections, R_{int}	152, 0.0976	123, 0.0585	125, 0.0596	124, 0.0651	125, 0.0513	242, 0.0589	127, 0.0484	131, 0.0467	130, 0.0668
Data/restraints/ parameters	152/6/13	123/6/13	125/0/13	124/0/13	125/0/13	242/0/13	127/0/13	131/0/13	130/0/13
GOOF	1.188	1.187	1.183	1.283	1.304	1.143	1.241	1.204	1.179
Final R indexes [I>=2σ (I)]	R_1 = 0.0406	R_1 = 0.0302	R_1 = 0.0324	R_1 = 0.0266	R_1 = 0.0204	R_1 = 0.0293	R_1 = 0.0214	R_1 = 0.0200	R_1 = 0.0481
	wR_2 = 0.1013	wR_2 = 0.0881	wR_2 = 0.0825	wR_2 = 0.0637	wR_2 = 0.0461	wR_2 = 0.0670	wR_2 = 0.0480	wR_2 = 0.0419	wR_2 = 0.1343
Final R indexes [all data]	R1 = 0.0430	R1 = 0.0311	R1 = 0.0328	R1 = 0.0281	R1 = 0.0224	R1 = 0.0329	R1 = 0.0237	R1 = 0.0221	R1 = 0.0487
	wR2 = 0.1026	wR2 = 0.0896	wR2 = 0.0829	wR2 = 0.0642	wR2 = 0.0469	wR2 = 0.0685	wR2 = 0.0489	wR2 = 0.0421	wR2 = 0.1344
Largest diff. peak/hole /e Å-3	5.26/-3.60	2.61/-1.80	3.90/-2.43	2.29/-2.73	2.19/-2.76	3.77/-2.44	1.41/-3.60	1.30/-2.07	6.01/-3.47
CCDC									

Table S2. Fractional Atomic Coordinates (×10^4) and Equivalent Isotropic Displacement Parameters (Å^2×10^3) for UCu_xBi_2 (x = 0.2 - 0.64). U_{eq} is defined as1/3 of the trace of the orthogonalized U_{IJ} tensor.

Atom		0.2	0.34	0.36	0.37	0.43	0.52	0.57	0.61	0.64
U1	x	7500	7500	7500	7500	7500	7500	7500	7500	7500
	y	7500	7500	7500	7500	7500	7500	7500	7500	7500
	z	2755.2(16)	2721.0(18)	2722.5(18)	2709.7(14)	2702.0(13)	2680.8(9)	2670.8(11)	2665.4(11)	2662(2)
	U(eq)	18.4(7)	14.5(8)	14.8(7)	17.9(6)	15.2(5)	14.9(3)	15.5(4)	16.9(4)	16.8(11)
Bi1	x	2500	2500	2500	2500	2500	2500	2500	2500	2500
	y	2500	2500	2500	2500	2500	2500	2500	2500	2500
	z	3562.6(15)	3525.9(18)	3529.9(18)	3514.6(14)	3503.9(14)	3476.1(10)	3461.7(11)	3451.6(12)	3452(2)
	U(eq)	20.5(6)	17.1(8)	17.7(7)	20.3(6)	18.1(5)	18.4(3)	19.2(4)	20.7(4)	20.7(11)
Bi2	x	7500	7500	7500	7500	7500	7500	7500	7500	7500
	y	2500	2500	2500	2500	2500	2500	2500	2500	2500
	z	0	0	0	0	0	0	0	0	0
	U(eq)	17.8(7)	13.8(7)	14.7(7)	17.6(6)	14.6(4)	14.8(3)	15.3(4)	16.7(4)	16.5(10)
Cu1	x	2500	2500	2500	2500	2500	2500	2500	2500	2500
	y	7500	7500	7500	7500	7500	7500	7500	7500	7500
	z	5000	5000	5000	5000	5000	5000	5000	5000	5000
	U(eq)	20(8)	15(5)	24(5)	16(4)	17(4)	16.4(15)	17(2)	20(2)	24(3)

Table S3. Anisotropic Displacement Parameters ($Å^2×10^3$) for UCu_xBi_2 (x = 0.2 - 0.64).

Atom		0.2	0.34	0.36	0.37	0.43	0.52	0.57	0.61	0.64
U1	U_{11}	20.4(8)	16.6(8)	15.3(8)	17.0(6)	15.5(5)	13.3(3)	15.4(5)	17.1(5)	15.1(11)
	U_{22}	20.4(8)	16.6(8)	15.3(8)	17.0(6)	15.5(5)	13.3(3)	15.4(5)	17.1(5)	15.1(11)
	U_{33}	14.3(9)	10.3(11)	13.9(10)	19.7(8)	14.6(7)	18.1(4)	15.7(6)	16.5(6)	20.2(17)
Bi1	U_{11}	22.2(8)	17.7(8)	16.7(8)	17.5(6)	16.2(6)	13.4(3)	15.6(5)	16.9(5)	15.1(11)
	U_{22}	22.2(8)	17.7(8)	16.7(8)	17.5(6)	16.2(6)	13.4(3)	15.6(5)	16.9(5)	15.1(11)
	U_{33}	17.3(9)	15.9(11)	19.8(11)	25.9(9)	21.8(8)	28.3(5)	26.3(7)	28.3(7)	32.0(19)
Bi2	U_{11}	19.8(8)	17.0(8)	15.9(7)	17.1(6)	15.1(5)	13.8(3)	15.7(5)	17.3(4)	15.2(11)
	U_{22}	19.8(8)	17.0(8)	15.9(7)	17.1(6)	15.1(5)	13.8(3)	15.7(5)	17.3(4)	15.2(11)
	U_{33}	13.9(10)	7.4(11)	12.5(10)	18.7(9)	13.6(7)	16.8(4)	14.6(6)	15.5(6)	19.2(15)
Cu1	U_{11}	23(8)	21(5)	29(6)	18(5)	21(4)	19(2)	19(2)	24(3)	26(3)
	U_{22}	23(8)	21(5)	29(6)	18(5)	21(4)	19(2)	19(2)	24(3)	26(3)
	U_{33}	15(10)	4(7)	13(8)	12(6)	8(5)	11(2)	11(3)	13(3)	20(7)

Table S4. Summary table of SEM EDS data of reaction HL34I. The reaction was a flux reaction with the ratio of 1:2:19 U:Cu:Bi.

			Atomic %'s:			Molar Ratios:			Average:		
**Sample ID:**	**Crystal #:**	**Site #:**	**U**	**Cu**	**Bi**	**U**	**Cu**	**Bi**	**U**	**Cu**	**Bi**
HL 34I	2	1	27.2	15.5	57.3	0.95	0.54	2	0.96	0.53	2
		2	27.7	14.0	58.3	0.95	0.48	2			
		3	27.7	16.0	56.3	0.98	0.57	2			

Table S5. Summary table of SEM EDS data of reaction HL29E. The reaction was a flux reaction with the ratio of 1:4:19 U:Cu:Bi.

			Atomic %'s:			Average:			Standard Deviation:			Molar Ratios:		
**Sample ID:**	**Crystal #:**	**Site #:**	**U**	**Cu**	**Bi**	**U**	**Cu**	**Bi**	**U**	**Cu**	**Bi**	**U**	**Cu**	**Bi**
HL 29E	1	1	27.2	16.6	56.2	27.00	16.73	56.27	0.20	0.12	0.12	0.97	0.59	2
		2	27.0	16.8	56.2							0.96	0.60	2
		3	26.8	16.8	56.4							0.95	0.60	2
	2	1	27.8	15.5	56.7	27.63	15.87	56.50	0.21	0.40	0.20	0.98	0.55	2
		2	27.7	15.8	56.5							0.98	0.56	2
		3	27.4	16.3	56.3							0.97	0.58	2
	3	1	27.5	16.5	56.0	27.47	16.27	56.30	0.06	0.25	0.30	0.98	0.59	2
		2	27.5	16.3	56.3							0.98	0.58	2
		3	27.4	16.0	56.6							0.97	0.57	2

Table S6. SEM EDS data for reaction HL34A. The reaction was a flux reaction with the ratio 1:3:13 U:Cu:Bi.

	Atomic %'s:			Molar Ratios:		
**Sample ID:**	**U**	**Cu**	**Bi**	**U**	**Cu**	**Bi**
HL 34A	26.6	17.6	55.8	0.95	0.63	2

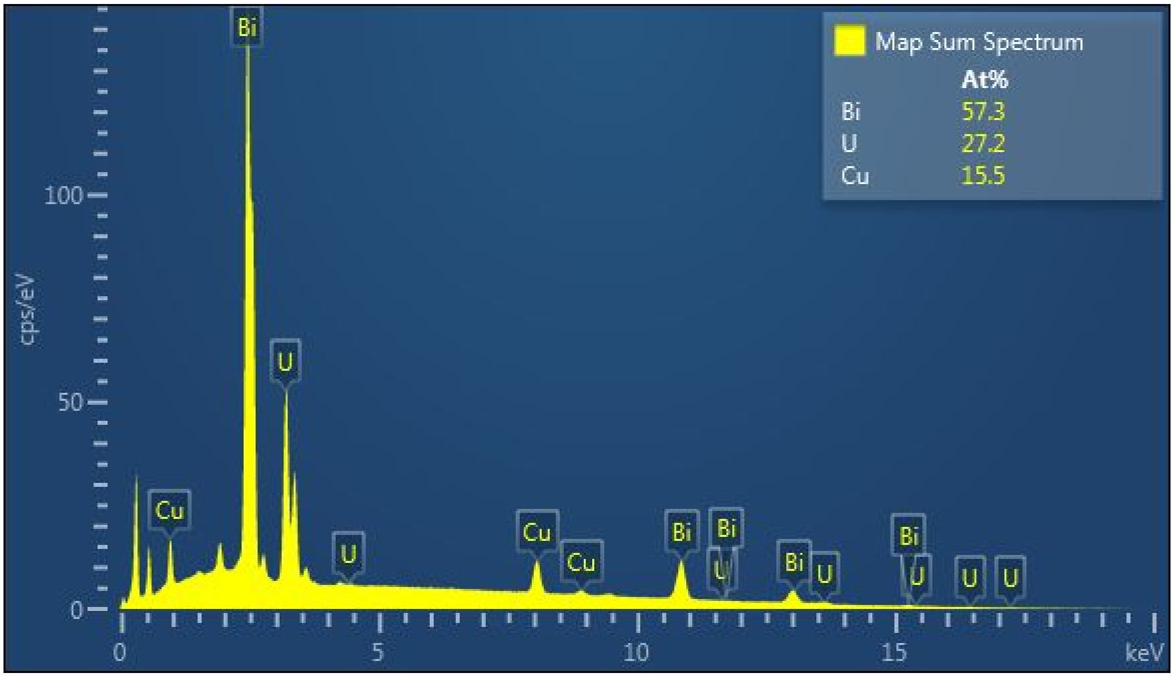

Figure S4. EDS spectrum of sample HL34I crystal 1 site 1

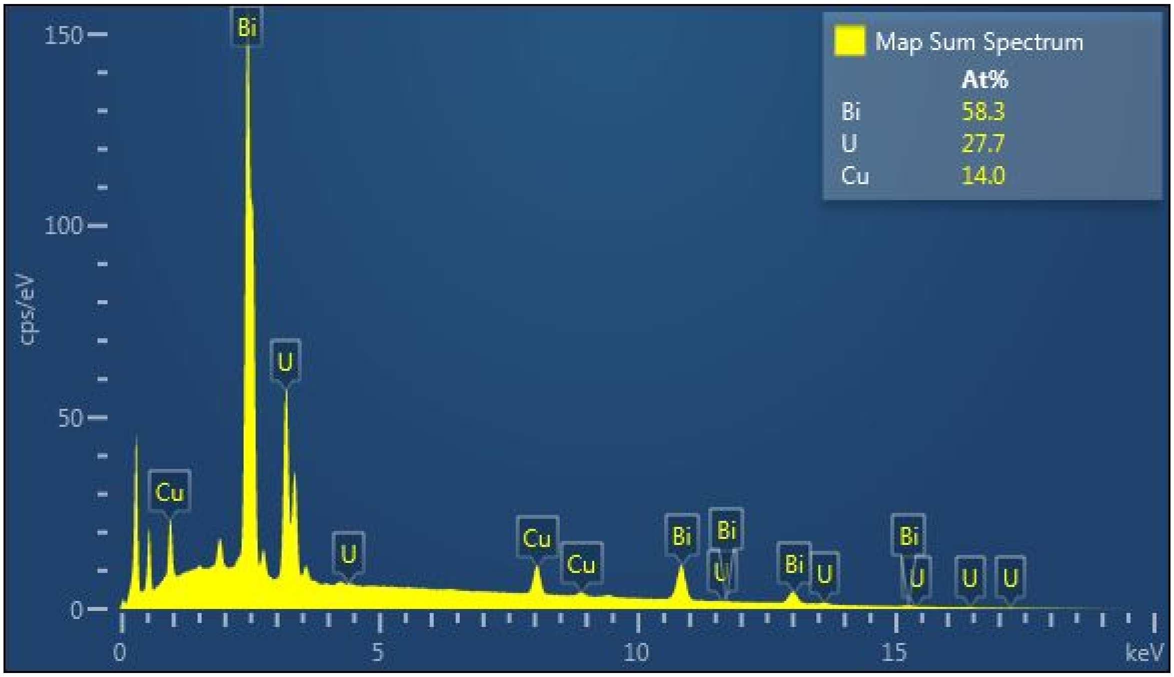

Figure S5. EDS spectrum of sample HL34I crystal 1 site 2

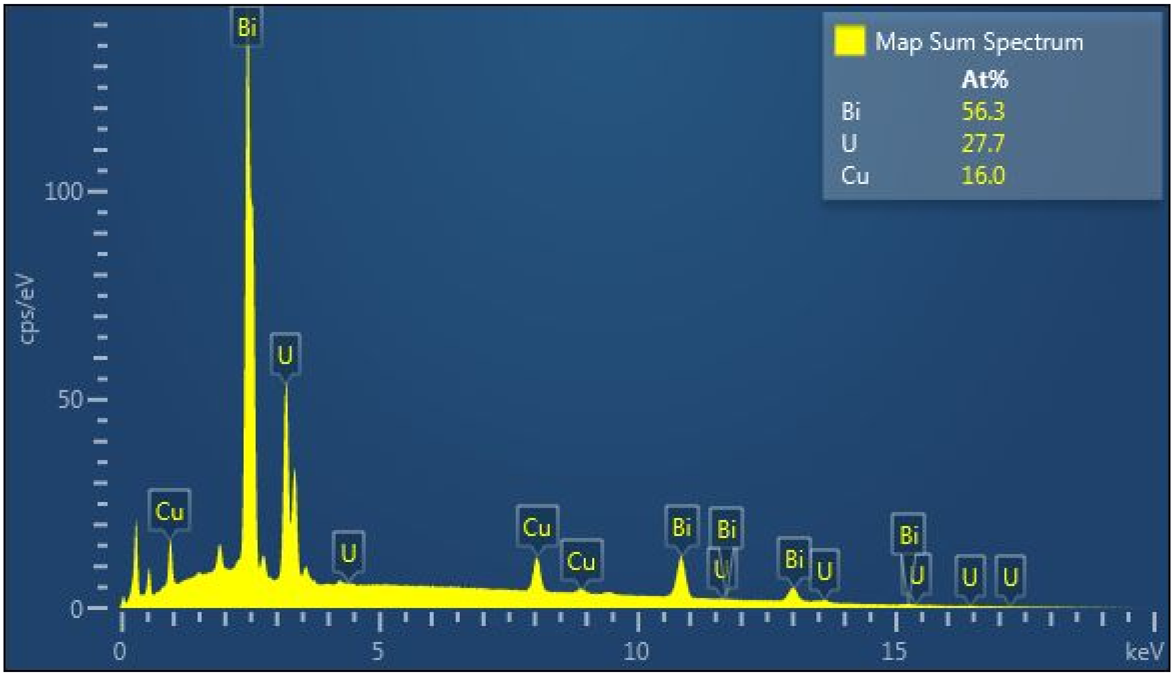

Figure S6. EDS spectrum of sample HL34I crystal 1 site 3

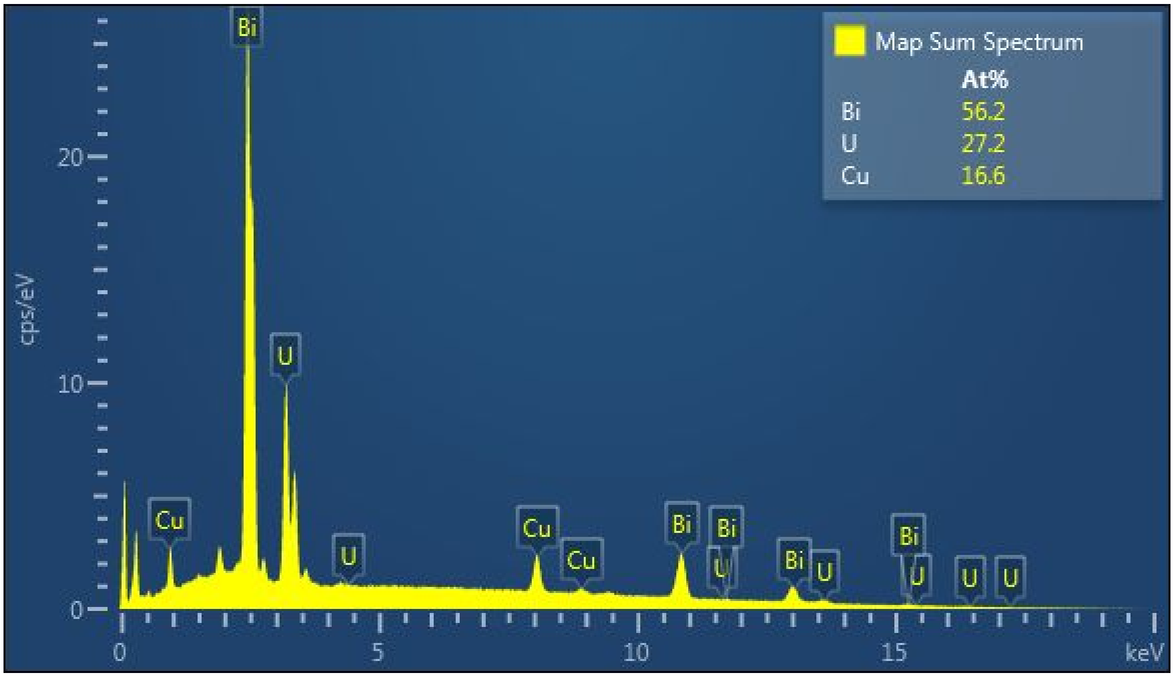

Figure S7. EDS spectrum of sample HL29E crystal 1 site 1

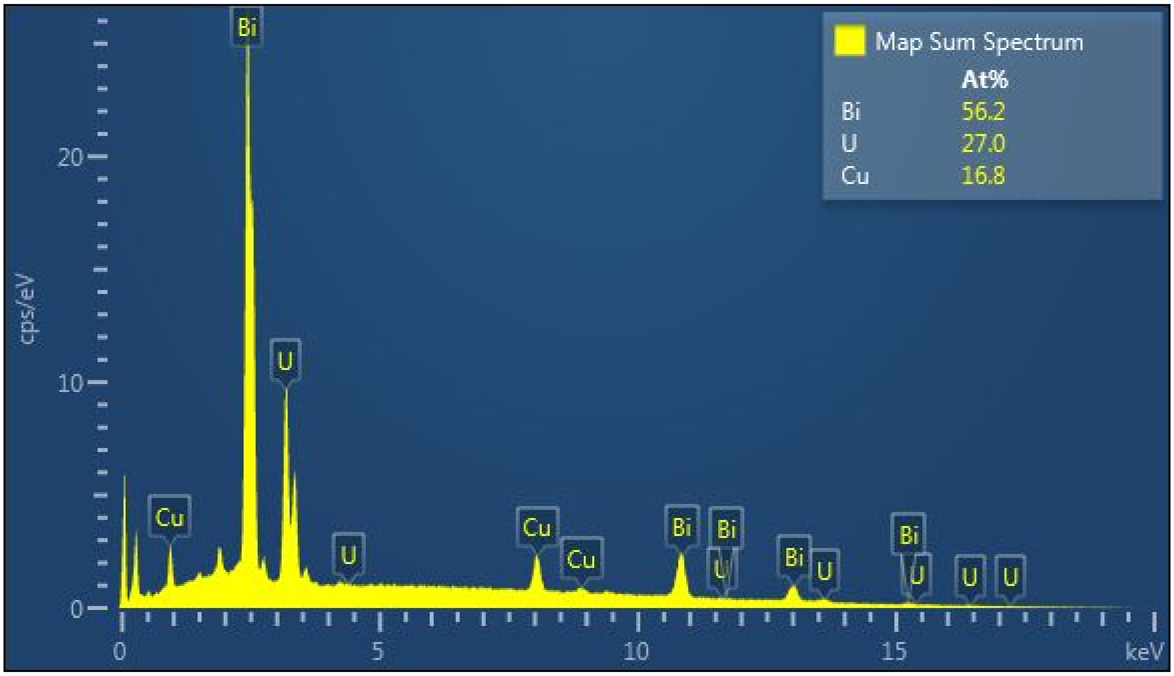

Figure S8. EDS spectrum of sample HL29E crystal 1 site 2

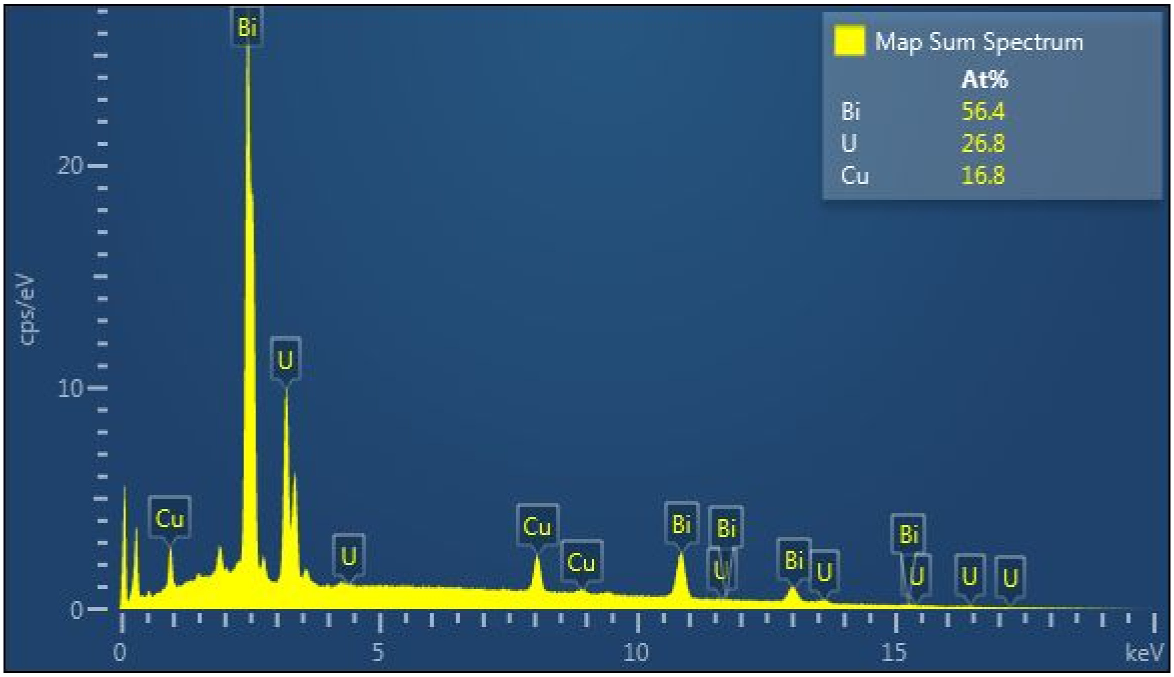

Figure S9. EDS spectrum of sample HL29E crystal 1 site 3

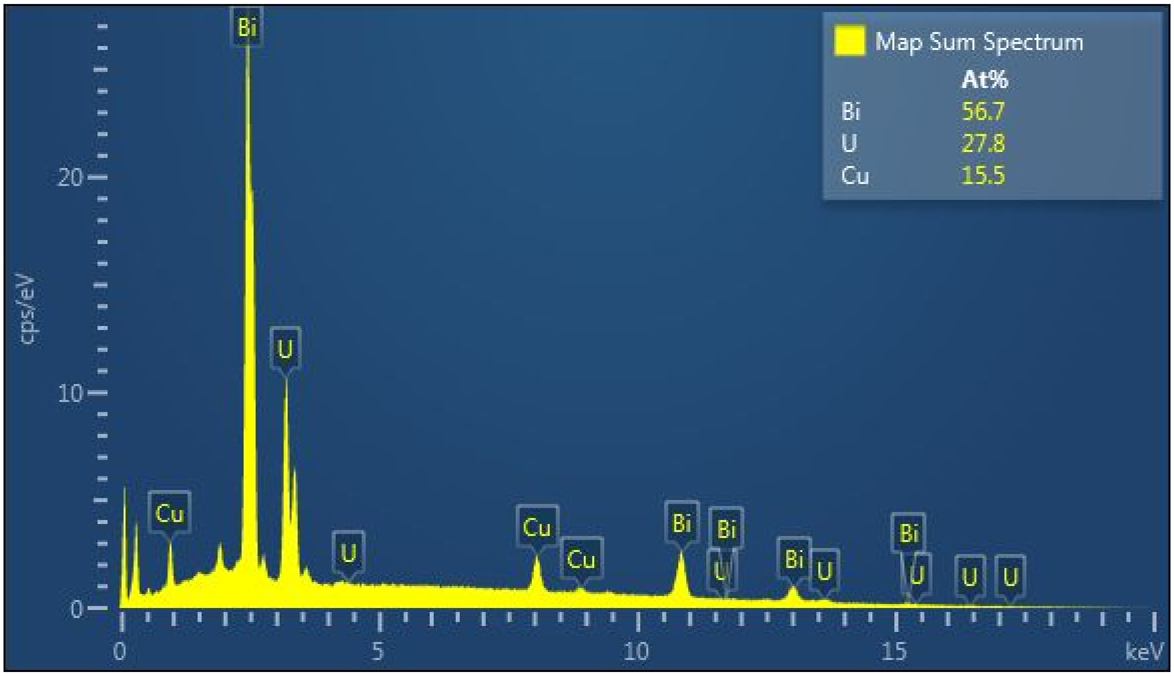

Figure S10. EDS spectrum of sample HL29E crystal 2 site 1

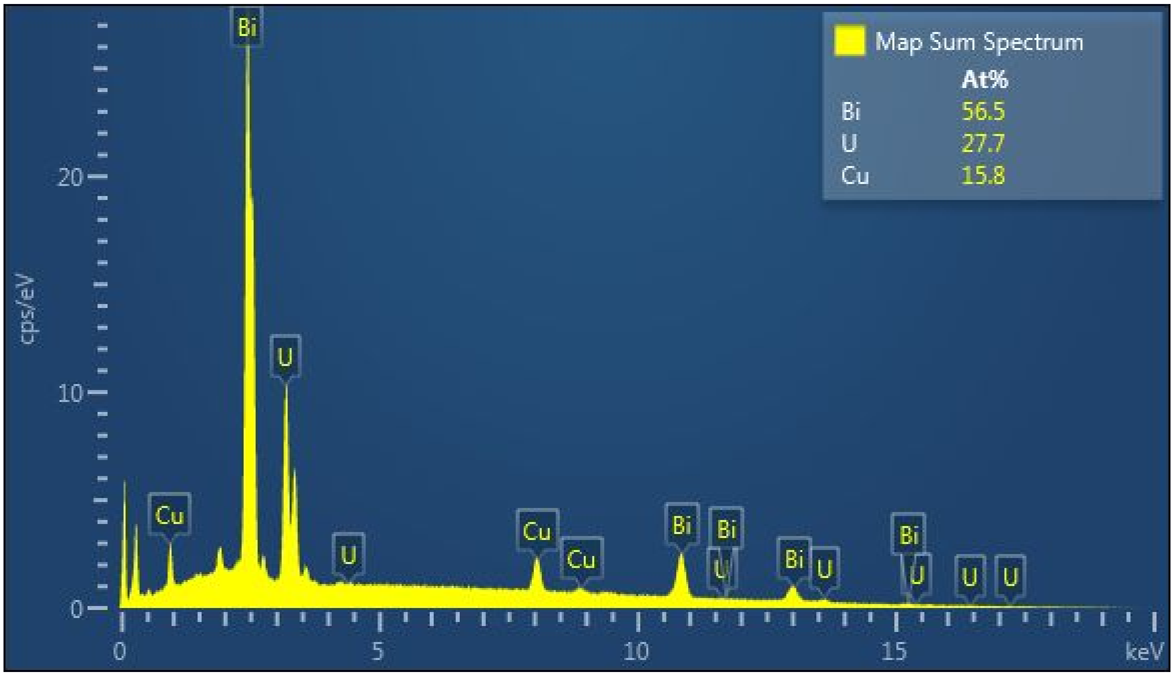

Figure S11. EDS spectrum of sample HL29E crystal 2 site 2

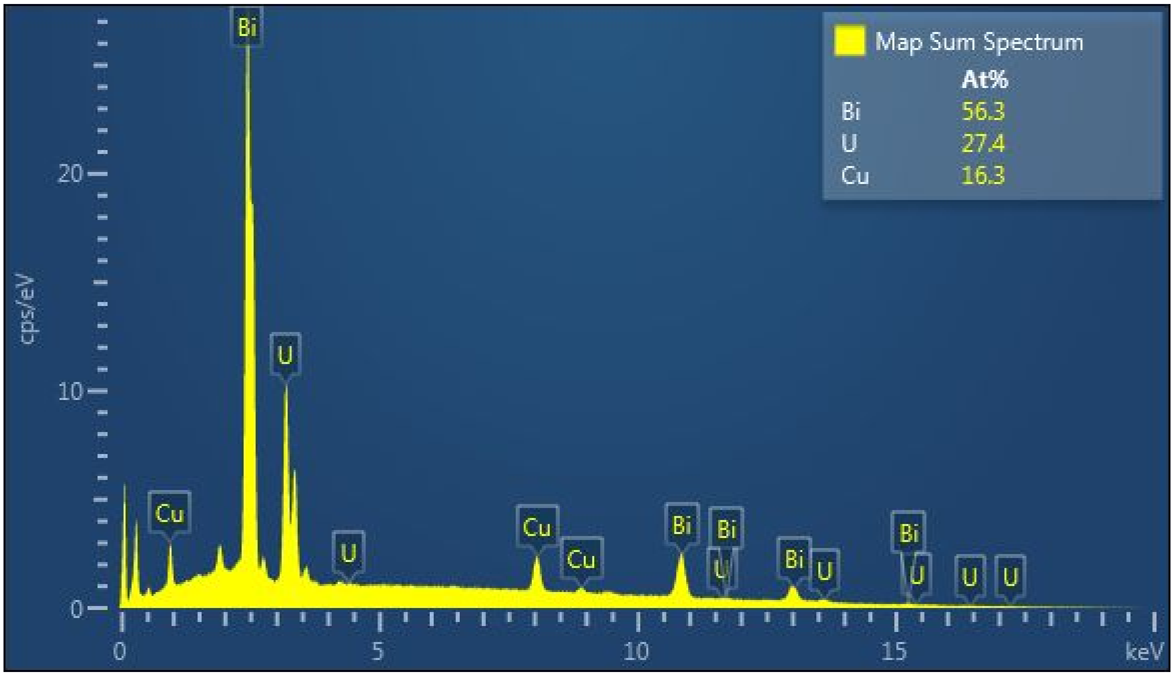

Figure S12. EDS spectrum of sample HL29E crystal 2 site 3

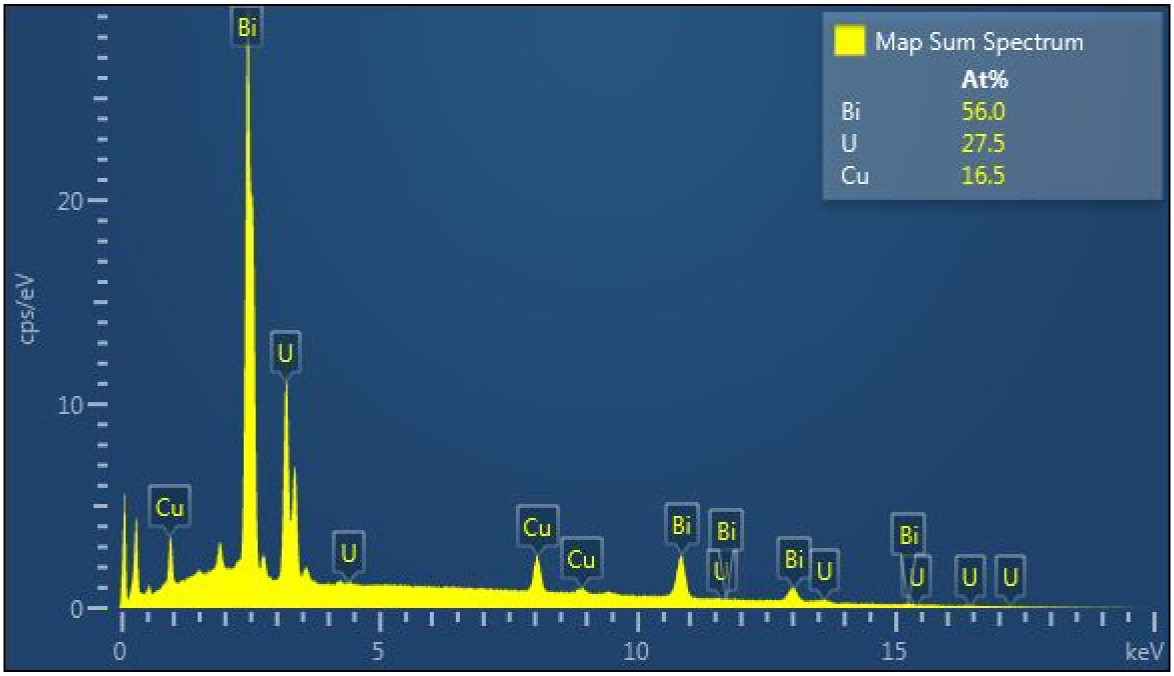

Figure S13. EDS spectrum of sample HL29E crystal 3 site 1

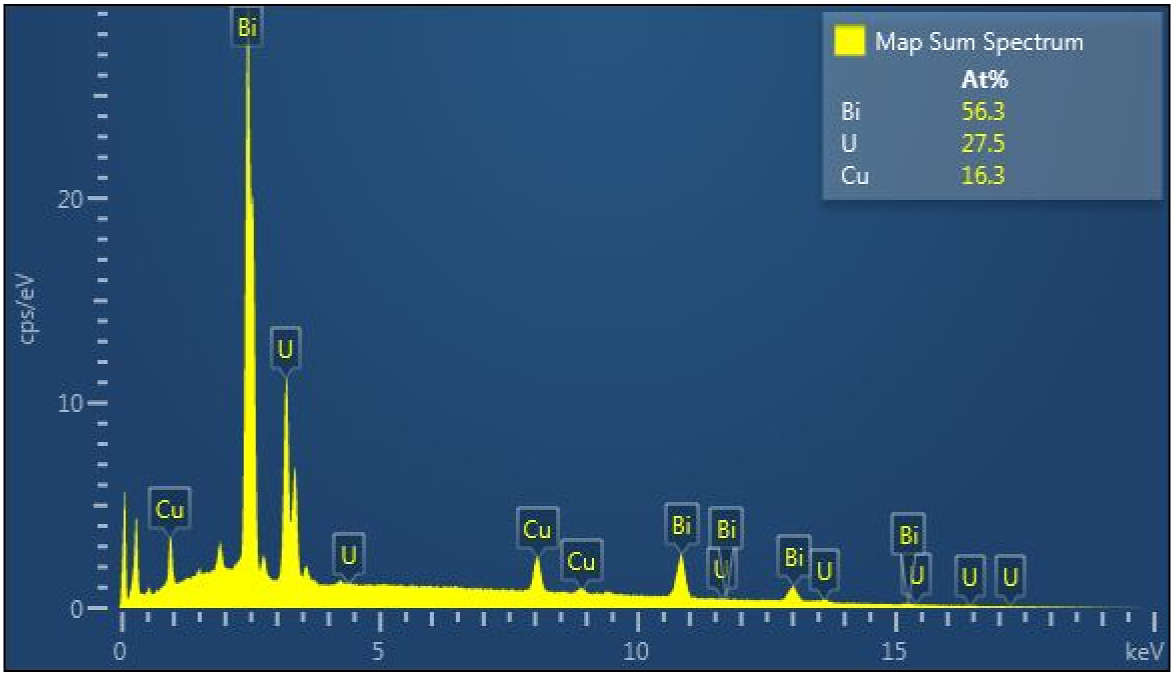

Figure S14. EDS spectrum of sample HL29E crystal 3 site 2

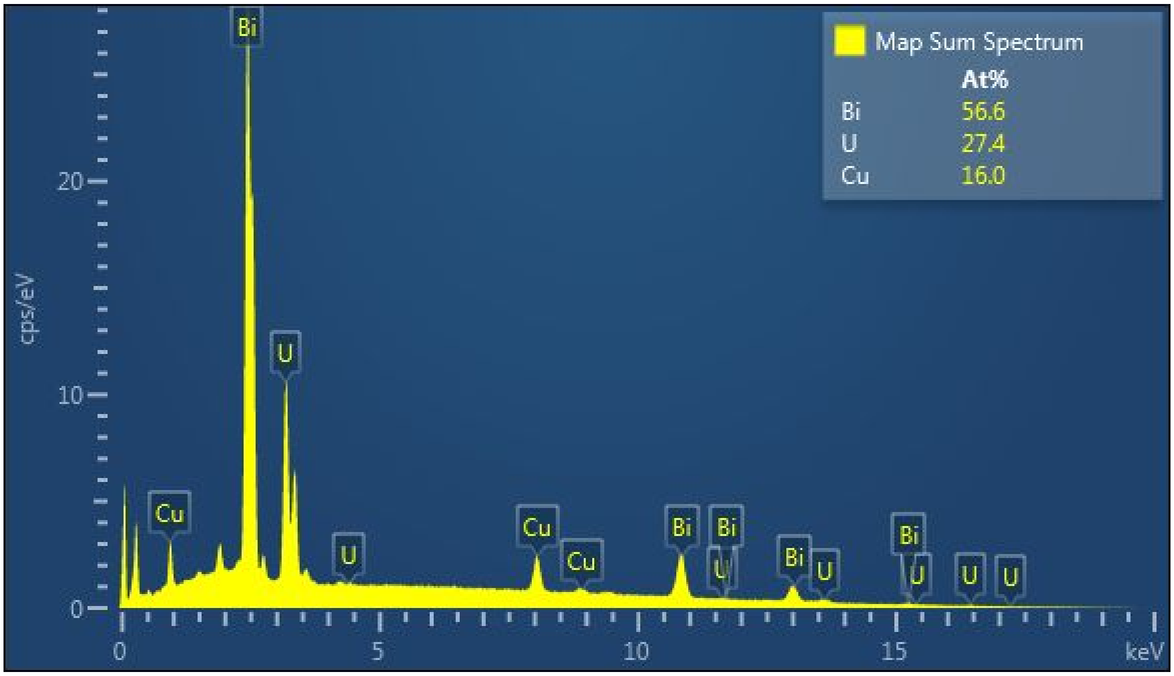

Figure S15. EDS spectrum of sample HL29E crystal 3 site 3

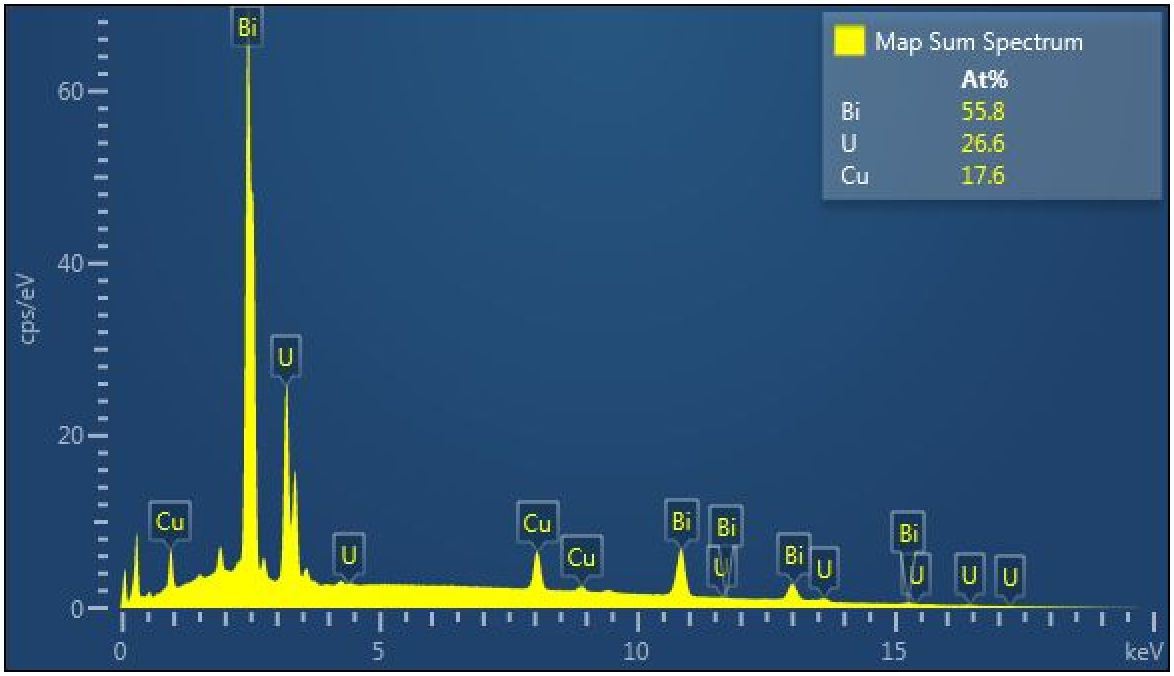

Figure S16. EDS spectrum of a sample HL34A.

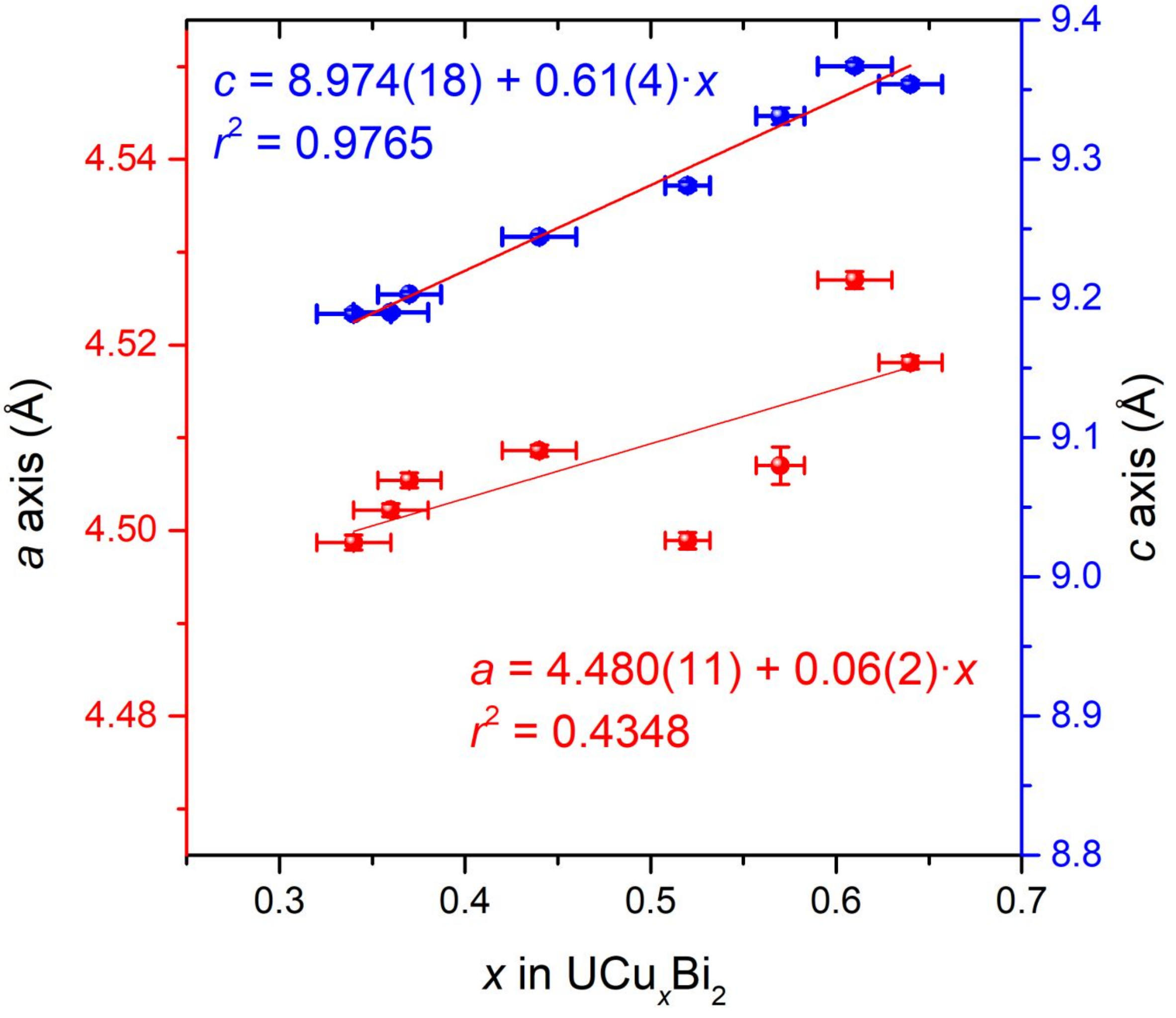

Figure S17. Unit cell parameters *a* and *c* fit within an *x* range of 0.3 – 0.64 in UCu_xBi_2 compositions.

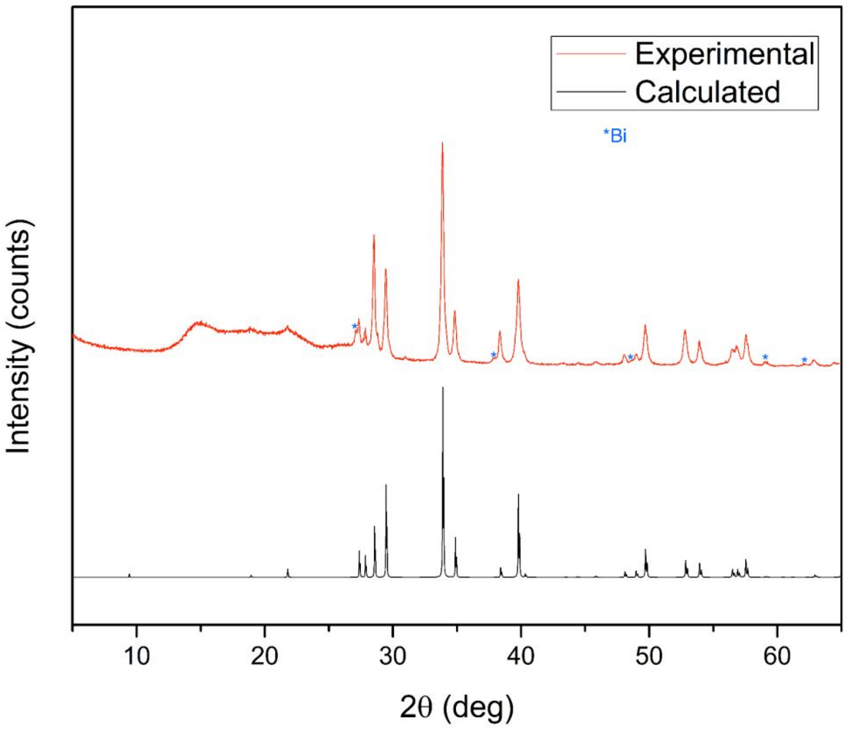

Figure S17. PXRD pattern of $UCu_{0.6}Bi_2$ obtained via arc melting and annealing at 800 °C. The broad reflection in the ~13-23° region is due to Kapton tape covering the sample.

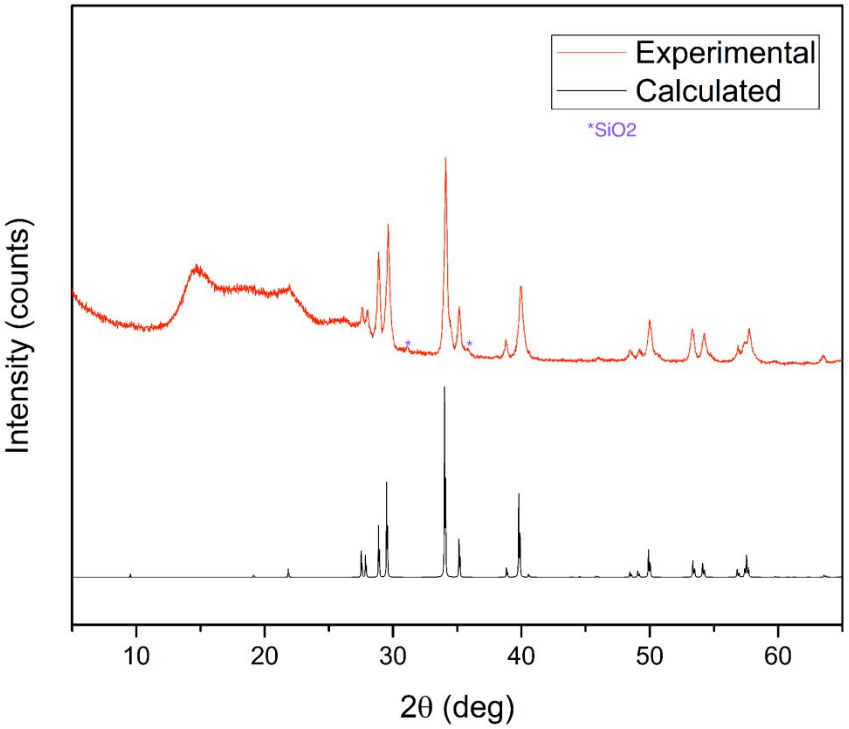

Figure S18. PXRD pattern of $UCu_{0.5}Bi_2$ obtained via arc melting and annealing at 800 °C. The broad reflection in the ~13-23° region is due to Kapton tape covering the sample.

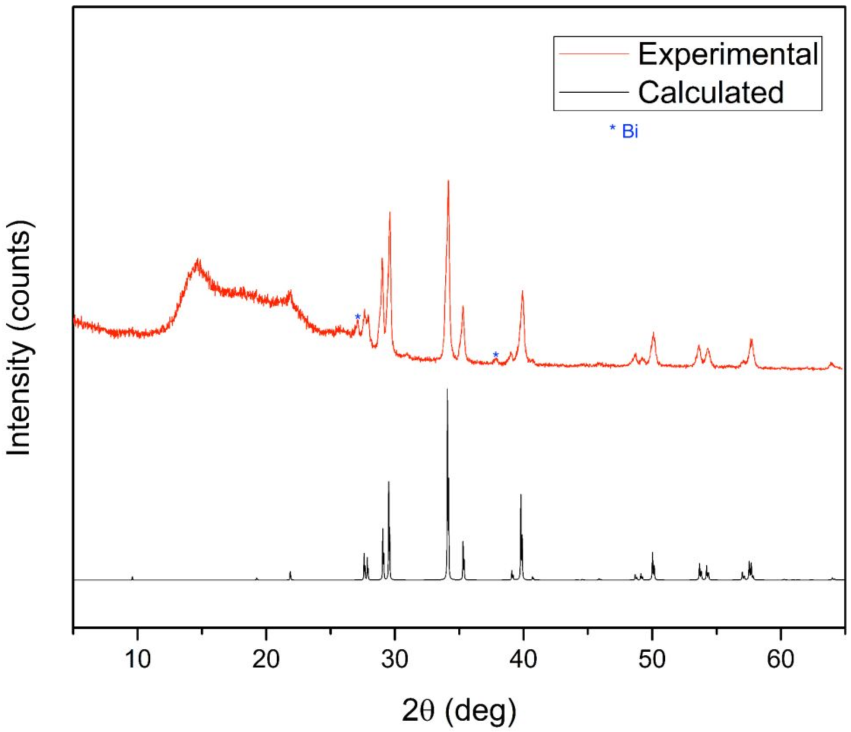

Figure S19. PXRD pattern of $UCu_{0.4}Bi_2$ obtained via arc melting and annealing at 800 °C. The broad reflection in the ~13-23° region is due to Kapton tape covering the sample.

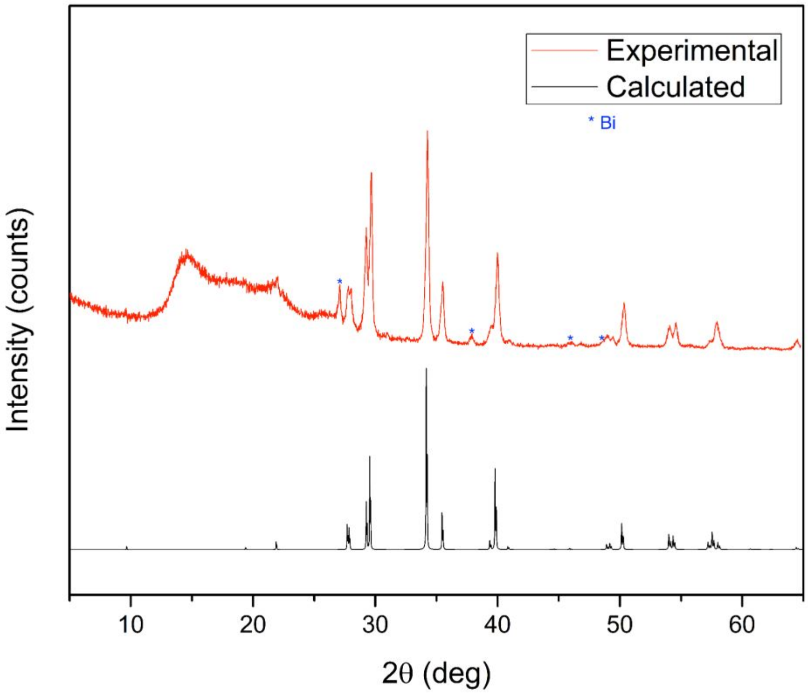

Figure S20. PXRD pattern of $UCu_{0.3}Bi_2$ obtained via arc melting and annealing at 800 °C. The broad reflection in the ~13-23° region is due to Kapton tape covering the sample.

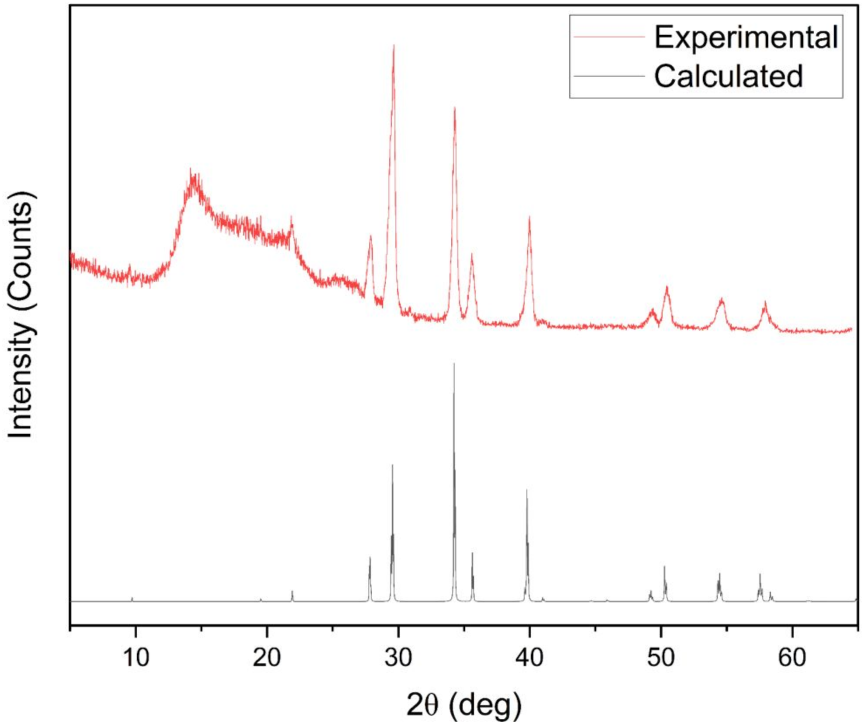

Figure S21. PXRD pattern of $UCu_{0.2}Bi_2$ obtained via arc melting and annealing at 800 °C. The broad reflection in the ~13-23° region is due to Kapton tape covering the sample.

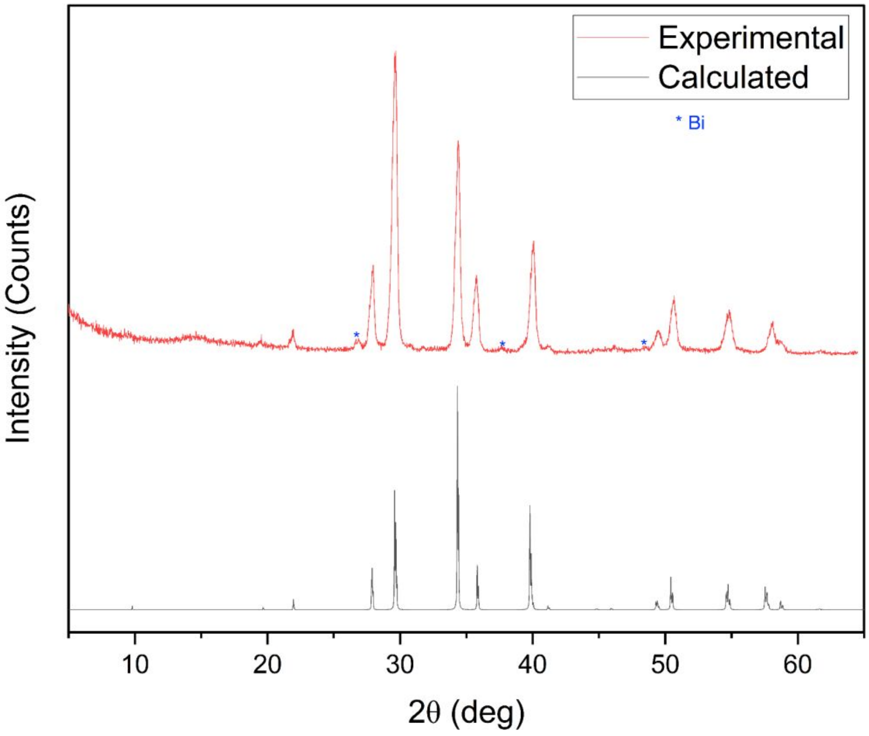

Figure S22. PXRD pattern of $UCu_{0.1}Bi_2$ obtained via arc melting and annealing at 800 °C.

Table S7. Optimized unit cell and atomic coordinates for FM ordered $UCu_{1/8}Bi_2$

```
VASP_CONTCAR_UCu1-8Bi2_FM
  1.00000000000000
    8.9941166458028814  -0.0000000000000000   0.0000000000000000
    0.0000000000000000   8.9941166458028814   0.0000000000000001
    0.0000000000000000  -0.0000000000000001   9.0709890043517429
  U   Bi  Cu
    8   16   1
Direct
  0.1250000000000000  0.6238949096664127  0.2785489413501370
  0.3761050903336149  0.8750000000000000  0.7214510586498826
  0.8738949096664127  0.8750000000000000  0.7214510586498826
  0.6250000000000000  0.6172929048425410  0.2829751820853861
  0.6250000000000000  0.1327070951574519  0.2829751820853861
  0.8672929048425410  0.3750000000000000  0.7170248179146215
  0.3827070951574573  0.3750000000000000  0.7170248179146215
  0.1250000000000000  0.1261050903335945  0.2785489413501370
  0.3753380055995045  0.6246619944004924  0.0000000000000000
  0.1250000000000000  0.6164154960602353  0.6508074545303743
  0.3835845039397655  0.8750000000000000  0.3491925454696276
  0.1250000000000000  0.8750000000000000  0.0000000000000000
  0.6250000000000000  0.8750000000000000  0.0031118898321877
  0.8664154960602353  0.8750000000000000  0.3491925454696276
  0.6250000000000000  0.6285365580760250  0.6421016349550858
  0.8746619944004924  0.6246619944004924  0.0000000000000000
  0.8746619944004924  0.1253380055995055  0.0000000000000000
  0.6250000000000000  0.1214634419239884  0.6421016349550858
  0.8785365580760250  0.3750000000000000  0.3578983650449141
  0.6250000000000000  0.3750000000000000  0.0000000000000000
  0.1250000000000000  0.3750000000000000  0.9968881101678173
  0.3714634419239881  0.3750000000000000  0.3578983650449141
  0.1250000000000000  0.1335845039397600  0.6508074545303743
  0.3753380055995045  0.1253380055995055  0.0000000000000000
  0.1250000000000000  0.8750000000000000  0.5000000000000000
```

Table S8. Optimized unit cell and atomic coordinates for FM ordered $UCu_{2/8}Bi_2$

```
VASP_CONTCAR_UCu2-8Bi2_FM
  1.00000000000000
    9.0199495727263770    0.0000000000000001    0.0000000000000000
    0.0000000000000000    9.0199495727263770    0.0000000000000000
    0.0000000000000000   -0.0000000000000000    9.1754467205723600
  U    Bi   Cu
    8    16     2
Direct
  0.1250000000000000  0.6298641493587981  0.2764911476696587
  0.3701358506412094  0.8750000000000000  0.7235088523303412
  0.8798641493587981  0.8750000000000000  0.7235088523303412
  0.6250000000000000  0.6201358506412019  0.2764911476696587
  0.6250000000000000  0.1298641493587976  0.2764911476696587
  0.8701358506412019  0.3750000000000000  0.7235088523303412
  0.3798641493587906  0.3750000000000000  0.7235088523303412
  0.1250000000000000  0.1201358506412094  0.2764911476696587
  0.3750000000000000  0.6250000000000000 -0.0000000000000000
  0.1250000000000000  0.6154765508516208  0.6483316828596328
  0.3845234491483794  0.8750000000000000  0.3516683171403735
  0.1250000000000000  0.8750000000000000 -0.0000000000000000
  0.6250000000000000  0.8750000000000000 -0.0000000000000000
  0.8654765508516208  0.8750000000000000  0.3516683171403735
  0.6250000000000000  0.6345234491483792  0.6483316828596328
  0.8750000000000000  0.6250000000000000 -0.0000000000000000
  0.8750000000000000  0.1250000000000000 -0.0000000000000000
  0.6250000000000000  0.1154765508516205  0.6483316828596328
  0.8845234491483792  0.3750000000000000  0.3516683171403735
  0.6250000000000000  0.3750000000000000 -0.0000000000000000
  0.1250000000000000  0.3750000000000000 -0.0000000000000000
  0.3654765508516206  0.3750000000000000  0.3516683171403735
  0.1250000000000000  0.1345234491483795  0.6483316828596328
  0.3750000000000000  0.1250000000000000 -0.0000000000000000
  0.1250000000000000  0.8750000000000000  0.5000000000000000
  0.6250000000000000  0.3750000000000000  0.5000000000000000
```

Table S9. Optimized unit cell and atomic coordinates for FM ordered $UCu_{3/8}Bi_2$

```
VASP_CONTCAR_UCu3-8Bi2_FM
  1.00000000000000
    9.0467151978720821    0.0000000000000000   -0.0000000000000000
   -0.0000000000000000    9.0467151978720821   -0.0000000000000000
    0.0000000000000000    0.0000000000000000    9.2497119121402473
  U    Bi   Cu
    8    16     3
Direct
  0.1250000000000000  0.6241836603058657  0.2764969738518753
  0.3704692576017491  0.8750000000000000  0.7268788103576362
  0.8795307423982515  0.8750000000000000  0.7268788103576362
  0.6250000000000000  0.6204692576017485  0.2731211896423782
  0.6250000000000000  0.1295307423982505  0.2731211896423782
  0.8758163396941343  0.3750000000000000  0.7235030261481326
  0.3741836603058661  0.3750000000000000  0.7235030261481326
  0.1250000000000000  0.1258163396941339  0.2764969738518753
  0.3741172113539180  0.6241172113539185  0.0000000000000000
  0.1250000000000000  0.6177393346994865  0.6473172848779265
  0.3760721938215496  0.8750000000000000  0.3389122252141113
  0.1250000000000000  0.8750000000000000  0.0013441653568235
  0.6250000000000000  0.8750000000000000  0.0000000000000000
  0.8739278061784438  0.8750000000000000  0.3389122252141113
  0.6250000000000000  0.6260721938215562  0.6610877747858823
  0.8758827886460815  0.6241172113539185  0.0000000000000000
  0.8758827886460815  0.1258827886460822  0.0000000000000000
  0.6250000000000000  0.1239278061784501  0.6610877747858823
  0.8822606653005135  0.3750000000000000  0.3526827151220885
  0.6250000000000000  0.3750000000000000  0.9986558346431774
  0.1250000000000000  0.3750000000000000  0.0000000000000000
  0.3677393346994859  0.3750000000000000  0.3526827151220885
  0.1250000000000000  0.1322606653005145  0.6473172848779265
  0.3741172113539180  0.1258827886460822  0.0000000000000000
  0.1250000000000000  0.8750000000000000  0.5004950671187306
  0.6250000000000000  0.8750000000000000  0.5000000000000000
  0.6250000000000000  0.3750000000000000  0.4995049328812703
```

Table S10. Optimized unit cell and atomic coordinates for FM ordered $UCu_{4/8}Bi_2$

```
VASP_CONTCAR_UCu4-8Bi2_FM
  1.00000000000000
    9.0647957809413260  -0.0000000000000000   0.0000000000000000
    0.0000000000000000   9.0647957809413260  -0.0000000000000000
    0.0000000000000000  -0.0000000000000000   9.3416544572637523
  U   Bi  Cu
    8   16    4
Direct
  0.1250000000000000  0.6250000000000000  0.2719146254156767
  0.3750000000000000  0.8750000000000000  0.7280853745843239
  0.8750000000000000  0.8750000000000000  0.7280853745843239
  0.6250000000000000  0.6250000000000000  0.2719146254156767
  0.6250000000000000  0.1250000000000000  0.2719146254156767
  0.8750000000000000  0.3750000000000000  0.7280853745843239
  0.3750000000000000  0.3750000000000000  0.7280853745843239
  0.1250000000000000  0.1250000000000000  0.2719146254156767
  0.3750000000000000  0.6250000000000000  0.0000000000000000
  0.1250000000000000  0.6250000000000000  0.6592866619497634
  0.3750000000000000  0.8750000000000000  0.3407133380502320
  0.1250000000000000  0.8750000000000000  0.0000000000000000
  0.6250000000000000  0.8750000000000000  0.0000000000000000
  0.8750000000000000  0.8750000000000000  0.3407133380502320
  0.6250000000000000  0.6250000000000000  0.6592866619497634
  0.8750000000000000  0.6250000000000000  0.0000000000000000
  0.8750000000000000  0.1250000000000000  0.0000000000000000
  0.6250000000000000  0.1250000000000000  0.6592866619497634
  0.8750000000000000  0.3750000000000000  0.3407133380502320
  0.6250000000000000  0.3750000000000000  0.0000000000000000
  0.1250000000000000  0.3750000000000000  0.0000000000000000
  0.3750000000000000  0.3750000000000000  0.3407133380502320
  0.1250000000000000  0.1250000000000000  0.6592866619497634
  0.3750000000000000  0.1250000000000000  0.0000000000000000
  0.1250000000000000  0.8750000000000000  0.5000000000000000
  0.6250000000000000  0.8750000000000000  0.5000000000000000
  0.6250000000000000  0.3750000000000000  0.5000000000000000
  0.1250000000000000  0.3750000000000000  0.5000000000000000
```

Table S11. Optimized unit cell and atomic coordinates for FM ordered $UCu_{5/8}Bi_2$

```
VASP_CONTCAR_UCu5-8Bi2_FM
  1.00000000000000
    9.0882128646842784  -0.0000000000000000   0.0000000000000000
    0.0000000000000000   9.0882128646842784   0.0000000000000000
    0.0000000000000000  -0.0000000000000000   9.3811669847877273
  U   Bi  Cu
    8   16   5
Direct
  0.1159960133256437  0.6250000000000000  0.2727546391095993
  0.3750000000000000  0.8763365562099834  0.7309601711094280
  0.8750000000000000  0.8659960133256480  0.7272453608903795
  0.6340039866743520  0.6250000000000000  0.2727546391095993
  0.6236634437900166  0.1250000000000000  0.2690398288905578
  0.8750000000000000  0.3840039866743622  0.7272453608903795
  0.3750000000000000  0.3736634437900385  0.7309601711094280
  0.1263365562099765  0.1250000000000000  0.2690398288905578
  0.3750000000000000  0.6250000000000000  0.0032882815150088
  0.1283167073677697  0.6250000000000000  0.6574134883138286
  0.3750000000000000  0.8695138321578986  0.3335935046785818
  0.1244636569369625  0.8744636569369758 -0.0000000000000000
  0.6255363430630242  0.8744636569369758 -0.0000000000000000
  0.8750000000000000  0.8783167073678131  0.3425865116861261
  0.6216832926321869  0.6250000000000000  0.6574134883138286
  0.8750000000000000  0.6250000000000000 -0.0000000000000000
  0.8750000000000000  0.1250000000000000  0.9967117184849899
  0.6304861678421014  0.1250000000000000  0.6664064953213865
  0.8750000000000000  0.3716832926322519  0.3425865116861261
  0.6255363430630242  0.3755363430630380 -0.0000000000000000
  0.1244636569369625  0.3755363430630380 -0.0000000000000000
  0.3750000000000000  0.3804861678420889  0.3335935046785818
  0.1195138321579179  0.1250000000000000  0.6664064953213865
  0.3750000000000000  0.1250000000000000 -0.0000000000000000
  0.1268546506054355  0.8768546506054220  0.5000000000000000
  0.6231453493945780  0.8768546506054220  0.5000000000000000
  0.6231453493945780  0.3731453493945715  0.5000000000000000
  0.1268546506054355  0.3731453493945715  0.5000000000000000
  0.3750000000000000  0.1250000000000000  0.5000000000000000
```

Table S12. Optimized unit cell and atomic coordinates for FM ordered $UCu_{6/8}Bi_2$

```
VASP_CONTCAR_UCu6-8Bi2_FM
  1.00000000000000
    9.1082209160031233    0.0000000000000000   -0.0000000000000000
   -0.0000000000000000    9.1082209160031233    0.0000000000000000
    0.0000000000000000   -0.0000000000000000    9.4210431655799525
  U    Bi   Cu
    8    16     6
Direct
  0.1167616699314377  0.6250000000000000  0.2697388330634098
  0.3750000000000000  0.8832383300685478  0.7302611669365902
  0.8750000000000000  0.8667616699314522  0.7302611669365902
  0.6332383300685478  0.6250000000000000  0.2697388330634098
  0.6167616699314522  0.1250000000000000  0.2697388330634098
  0.8750000000000000  0.3832383300685623  0.7302611669365902
  0.3750000000000000  0.3667616699314377  0.7302611669365902
  0.1332383300685623  0.1250000000000000  0.2697388330634098
  0.3750000000000000  0.6250000000000000 -0.0000000000000000
  0.1328669870012816  0.6250000000000000  0.6645718105254462
  0.3750000000000000  0.8671330129986971  0.3354281894745538
  0.1250000000000000  0.8750000000000000 -0.0000000000000000
  0.6250000000000000  0.8750000000000000 -0.0000000000000000
  0.8750000000000000  0.8828669870013029  0.3354281894745538
  0.6171330129986971  0.6250000000000000  0.6645718105254462
  0.8750000000000000  0.6250000000000000 -0.0000000000000000
  0.8750000000000000  0.1250000000000000 -0.0000000000000000
  0.6328669870013029  0.1250000000000000  0.6645718105254462
  0.8750000000000000  0.3671330129987043  0.3354281894745538
  0.6250000000000000  0.3750000000000000 -0.0000000000000000
  0.1250000000000000  0.3750000000000000 -0.0000000000000000
  0.3750000000000000  0.3828669870012957  0.3354281894745538
  0.1171330129987184  0.1250000000000000  0.6645718105254462
  0.3750000000000000  0.1250000000000000 -0.0000000000000000
  0.1250000000000000  0.8750000000000000  0.5000000000000000
  0.6250000000000000  0.8750000000000000  0.5000000000000000
  0.8750000000000000  0.6250000000000000  0.5000000000000000
  0.6250000000000000  0.3750000000000000  0.5000000000000000
  0.1250000000000000  0.3750000000000000  0.5000000000000000
  0.3750000000000000  0.1250000000000000  0.5000000000000000
```

Table S13. Optimized unit cell and atomic coordinates for FM ordered $UCu_{7/8}Bi_2$

```
VASP_CONTCAR_UCu7-8Bi2_FM
  1.00000000000000
    9.1171241449556391  -0.0000000000000001   0.0000000000000000
   -0.0000000000000001   9.1171241449556391   0.0000000000000000
    0.0000000000000000   0.0000000000000000   9.4538579391914475
  U   Bi   Cu
    8   16    7
Direct
  0.1238926058806409  0.6250000000000000  0.2705286652057323
  0.3750000000000000  0.8761073941193496  0.7294713347942746
  0.8750000000000000  0.8679976221794236  0.7325128556404732
  0.6261073941193496  0.6250000000000000  0.2705286652057323
  0.6179976221794236  0.1250000000000000  0.2674871443595090
  0.8750000000000000  0.3820023778205538  0.7325128556404732
  0.3750000000000000  0.3738926058806301  0.7294713347942746
  0.1320023778205551  0.1250000000000000  0.2674871443595090
  0.3750000000000000  0.6250000000000000  0.0000000000000000
  0.1300650719992167  0.6250000000000000  0.6630815747040391
  0.3750000000000000  0.8699349280007860  0.3369184252959629
  0.1256484784007344  0.8743515215992779  0.0000000000000000
  0.6243515215992779  0.8743515215992779  0.0000000000000000
  0.8750000000000000  0.8775961564159894  0.3251020585507928
  0.6199349280007860  0.6250000000000000  0.6630815747040391
  0.8750000000000000  0.6250000000000000  0.0019563770070832
  0.8750000000000000  0.1250000000000000  0.0000000000000000
  0.6275961564159894  0.1250000000000000  0.6748979414491644
  0.8750000000000000  0.3724038435840104  0.3251020585507928
  0.6243515215992779  0.3756484784007250  0.0000000000000000
  0.1256484784007344  0.3756484784007250  0.0000000000000000
  0.3750000000000000  0.3800650719992235  0.3369184252959629
  0.1224038435840059  0.1250000000000000  0.6748979414491644
  0.3750000000000000  0.1250000000000000  0.9980436229929299
  0.1222458830943066  0.8777541169056938  0.5000000000000000
  0.6277541169056938  0.8777541169056938  0.5000000000000000
  0.8750000000000000  0.6250000000000000  0.4977308104770882
  0.8750000000000000  0.1250000000000000  0.5000000000000000
  0.6277541169056938  0.3722458830943292  0.5000000000000000
  0.1222458830943066  0.3722458830943292  0.5000000000000000
  0.3750000000000000  0.1250000000000000  0.5022691895229043
```

Table S14. Optimized unit cell and atomic coordinates for FM ordered $UCu_{8/8}Bi_2$

```
VASP_CONTCAR_UCu8-8Bi2_FM
  1.00000000000000
    9.1294469503003821  -0.0000000000000007   0.0000000000000001
    0.0000000000000001   9.1294469503003821  -0.0000000000000001
    0.0000000000000001   0.0000000000000001   9.4698632528808435
  U   Bi   Cu
    8   16    8
Direct
  0.1250000000000000  0.6250000000000000  0.2687631583115363
  0.3750000000000000  0.8750000000000000  0.7312368416885812
  0.8750000000000000  0.8750000000000000  0.7312368416885812
  0.6250000000000000  0.6250000000000000  0.2687631583115363
  0.6250000000000000  0.1250000000000000  0.2687631583115363
  0.8750000000000000  0.3750000000000000  0.7312368416885812
  0.3750000000000000  0.3750000000000000  0.7312368416885812
  0.1250000000000000  0.1250000000000000  0.2687631583115363
  0.3750000000000000  0.6250000000000000  0.0000000000000000
  0.1250000000000000  0.6250000000000000  0.6732987279347696
  0.3750000000000000  0.8750000000000000  0.3267012720652344
  0.1250000000000000  0.8750000000000000  0.0000000000000000
  0.6250000000000000  0.8750000000000000  0.0000000000000000
  0.8750000000000000  0.8750000000000000  0.3267012720652344
  0.6250000000000000  0.6250000000000000  0.6732987279347696
  0.8750000000000000  0.6250000000000000  0.0000000000000000
  0.8750000000000000  0.1250000000000000  0.0000000000000000
  0.6250000000000000  0.1250000000000000  0.6732987279347696
  0.8750000000000000  0.3750000000000000  0.3267012720652344
  0.6250000000000000  0.3750000000000000  0.0000000000000000
  0.1250000000000000  0.3750000000000000  0.0000000000000000
  0.3750000000000000  0.3750000000000000  0.3267012720652344
  0.1250000000000000  0.1250000000000000  0.6732987279347696
  0.3750000000000000  0.1250000000000000  0.0000000000000000
  0.3750000000000000  0.6250000000000000  0.5000000000000000
  0.1250000000000000  0.8750000000000000  0.5000000000000000
  0.6250000000000000  0.8750000000000000  0.5000000000000000
  0.8750000000000000  0.6250000000000000  0.5000000000000000
  0.8750000000000000  0.1250000000000000  0.5000000000000000
  0.6250000000000000  0.3750000000000000  0.5000000000000000
  0.1250000000000000  0.3750000000000000  0.5000000000000000
  0.3750000000000000  0.1250000000000000  0.5000000000000000
```

Table S15. Optimized unit cell and atomic coordinates for AFM ordered $UCu_{1/8}Bi_2$

```
VASP_CONTCAR_UCu1-8Bi2_AFM
  1.00000000000000
    9.0231208116737776  -0.0025942276541399   0.0000000000000000
   -0.0025942276541399   9.0231208116737758   0.0000000000000001
   -0.0000000000000000  -0.0000000000000001   9.0425372597669540
  U   Bi   Cu
    8   16    1
Direct
  0.1243945536876516  0.6235748825613512  0.2780928215711944
  0.3764251174386773  0.8756054463123524  0.7219071784288243
  0.8735748825613512  0.8743945536876476  0.7219071784288243
  0.6253366291275567  0.6181287952347264  0.2826968434527208
  0.6246633708724433  0.1318712047652663  0.2826968434527208
  0.8681287952347264  0.3753366291275568  0.7173031565472912
  0.3818712047652732  0.3746633708724432  0.7173031565472912
  0.1256054463123479  0.1264251174386560  0.2780928215711944
  0.3753118203821886  0.6246881796178088  0.0000000000000000
  0.1243086958280748  0.6164324974668752  0.6494783813302619
  0.3835675025331263  0.8756913041719240  0.3505216186697389
  0.1250000000000000  0.8750000000000000  0.0000000000000000
  0.6250000000000000  0.8750000000000000  0.0038516962474088
  0.8664324974668752  0.8743086958280760  0.3505216186697389
  0.6253006447746796  0.6277051381345053  0.6430848028005497
  0.8747333216972779  0.6247333216972779  0.0000000000000000
  0.8746881796178088  0.1253118203821860  0.0000000000000000
  0.6246993552253204  0.1222948618655138  0.6430848028005497
  0.8777051381345053  0.3753006447746772  0.3569151971994561
  0.6250000000000000  0.3750000000000000  0.0000000000000000
  0.1250000000000000  0.3750000000000000  0.9961483037526008
  0.3722948618655148  0.3746993552253228  0.3569151971994561
  0.1256913041719254  0.1335675025331191  0.6494783813302619
  0.3752666783027170  0.1252666783027196  0.0000000000000000
  0.1250000000000000  0.8750000000000000  0.5000000000000000
```

Table S16. Optimized unit cell and atomic coordinates for AFM ordered $UCu_{2/8}Bi_2$

```
VASP_CONTCAR_UCu2-8Bi2_AFM
  1.00000000000000
    9.0433939601528373    0.0001474639821787   -0.0000000000000000
    0.0001474639821786    9.0433939601528390   -0.0000000000000000
    0.0000000000000000    0.0000000000000000    9.1493612680658902
  U    Bi   Cu
    8    16     2
Direct
  0.1250421554658238  0.6305230280445788  0.2761428483152504
  0.3694769719554226  0.8749578445341704  0.7238571516847497
  0.8805230280445788  0.8750421554658296  0.7238571516847497
  0.6249578445341704  0.6194769719554212  0.2761428483152504
  0.6250421554658296  0.1305230280445852  0.2761428483152504
  0.8694769719554212  0.3749578445341765  0.7238571516847497
  0.3805230280445774  0.3750421554658235  0.7238571516847497
  0.1249578445341765  0.1194769719554217  0.2761428483152504
  0.3750000000000000  0.6250000000000000 -0.0000000000000000
  0.1249911650469561  0.6149290845133603  0.6474055843196745
  0.3850709154866463  0.8750088349530436  0.3525944156803244
  0.1250000000000000  0.8750000000000000 -0.0000000000000000
  0.6250000000000000  0.8750000000000000 -0.0000000000000000
  0.8649290845133603  0.8749911650469564  0.3525944156803244
  0.6250088349530436  0.6350709154866397  0.6474055843196745
  0.8750000000000000  0.6250000000000000 -0.0000000000000000
  0.8750000000000000  0.1250000000000000 -0.0000000000000000
  0.6249911650469564  0.1149290845133610  0.6474055843196745
  0.8850709154866397  0.3750088349530437  0.3525944156803244
  0.6250000000000000  0.3750000000000000 -0.0000000000000000
  0.1250000000000000  0.3750000000000000 -0.0000000000000000
  0.3649290845133537  0.3749911650469563  0.3525944156803244
  0.1250088349530437  0.1350709154866461  0.6474055843196745
  0.3750000000000000  0.1250000000000000 -0.0000000000000000
  0.1250000000000000  0.8750000000000000  0.5000000000000000
  0.6250000000000000  0.3750000000000000  0.5000000000000000
```

Table S17. Optimized unit cell and atomic coordinates for AFM ordered $UCu_{3/8}Bi_2$

```
VASP_CONTCAR_UCu3-8Bi2_AFM
   1.00000000000000
     9.0693044754729701    0.0031198385413072    0.0000000000000000
     0.0031198385413072    9.0693044754729808    0.0000000000000000
     0.0000000000000000    0.0000000000000000    9.2318622067971408
   U    Bi   Cu
     8    16     3
Direct
  0.1251777104313595  0.6245747699383776  0.2757611910398125
  0.3688619315876467  0.8748470231274319  0.7277140510399071
  0.8811380684123521  0.8751529768725681  0.7277140510399071
  0.6248470231274319  0.6188619315876479  0.2722859489601159
  0.6251529768725681  0.1311380684123495  0.2722859489601159
  0.8754252300616224  0.3748222895686331  0.7242388089601741
  0.3745747699383797  0.3751777104313669  0.7242388089601741
  0.1248222895686370  0.1254252300616241  0.2757611910398125
  0.3742151521675734  0.6242151521675596 -0.0000000000000000
  0.1255325897478071  0.6171395472626705  0.6466841523518435
  0.3763649032047961  0.8755222305934746  0.3400261749730596
  0.1250000000000000  0.8750000000000000  0.0020307369550128
  0.6250000000000000  0.8750000000000000 -0.0000000000000000
  0.8736350967951992  0.8744777694065254  0.3400261749730596
  0.6255222305934746  0.6263649032048008  0.6599738250269495
  0.8757426789491601  0.6242573210508399 -0.0000000000000000
  0.8757848478324404  0.1257848478324218 -0.0000000000000000
  0.6244777694065254  0.1236350967952067  0.6599738250269495
  0.8828604527373295  0.3744674102521839  0.3533158476481611
  0.6250000000000000  0.3750000000000000  0.9979692630449903
  0.1250000000000000  0.3750000000000000 -0.0000000000000000
  0.3671395472626746  0.3755325897478161  0.3533158476481611
  0.1244674102521903  0.1328604527373307  0.6466841523518435
  0.3742573210508285  0.1257426789491693 -0.0000000000000000
  0.1250000000000000  0.8750000000000000  0.5005637830141945
  0.6250000000000000  0.8750000000000000  0.5000000000000000
  0.6250000000000000  0.3750000000000000  0.4994362169857880
```

Table S18. Optimized unit cell and atomic coordinates for AFM ordered $UCu_{4/8}Bi_2$

```
VASP_CONTCAR_UCu4-8Bi2_AFM
  1.00000000000000
    9.0848625030469634    0.0083519541872023   -0.0000000000000000
    0.0083519541872023    9.0848625030469314    0.0000000000000000
    0.0000000000000000    0.0000000000000000    9.3197885674495691
  U    Bi   Cu
    8    16     4
Direct
  0.1250000000000000  0.6250000000000000  0.2713255842275566
  0.3750000000000000  0.8750000000000000  0.7286744157724416
  0.8750000000000000  0.8750000000000000  0.7286744157724416
  0.6250000000000000  0.6250000000000000  0.2713255842275566
  0.6250000000000000  0.1250000000000000  0.2713255842275566
  0.8750000000000000  0.3750000000000000  0.7286744157724416
  0.3750000000000000  0.3750000000000000  0.7286744157724416
  0.1250000000000000  0.1250000000000000  0.2713255842275566
  0.3750000000000000  0.6250000000000000  0.0000000000000000
  0.1250000000000000  0.6250000000000000  0.6587186228293198
  0.3750000000000000  0.8750000000000000  0.3412813771706795
  0.1250000000000000  0.8750000000000000  0.0000000000000000
  0.6250000000000000  0.8750000000000000  0.0000000000000000
  0.8750000000000000  0.8750000000000000  0.3412813771706795
  0.6250000000000000  0.6250000000000000  0.6587186228293198
  0.8750000000000000  0.6250000000000000  0.0000000000000000
  0.8750000000000000  0.1250000000000000  0.0000000000000000
  0.6250000000000000  0.1250000000000000  0.6587186228293198
  0.8750000000000000  0.3750000000000000  0.3412813771706795
  0.6250000000000000  0.3750000000000000  0.0000000000000000
  0.1250000000000000  0.3750000000000000  0.0000000000000000
  0.3750000000000000  0.3750000000000000  0.3412813771706795
  0.1250000000000000  0.1250000000000000  0.6587186228293198
  0.3750000000000000  0.1250000000000000  0.0000000000000000
  0.1250000000000000  0.8750000000000000  0.5000000000000000
  0.6250000000000000  0.8750000000000000  0.5000000000000000
  0.6250000000000000  0.3750000000000000  0.5000000000000000
  0.1250000000000000  0.3750000000000000  0.5000000000000000
```

Table S19. Optimized unit cell and atomic coordinates for AFM ordered $UCu_{5/8}Bi_2$

```
VASP_CONTCAR_UCu5-8Bi2_AFM
  1.00000000000000
    9.1110288028352855    0.0098628337471828    0.0000000000000000
    0.0098628337471827    9.1110288028352855    0.0000000000000000
    0.0000000000000000    0.0000000000000000    9.3449646203518686
  U    Bi   Cu
    8    16     5
Direct
  0.1169052554593861  0.6255351503479627  0.2727802036394319
  0.3736699337832633  0.8769233470855100  0.7303248013648853
  0.8755351503479627  0.8669052554593971  0.7272197963605362
  0.6330947445406029  0.6244648496520373  0.2727802036394319
  0.6230766529144900  0.1263300662167398  0.2696751986350950
  0.8744648496520373  0.3830947445406179  0.7272197963605362
  0.3763300662167367  0.3730766529145312  0.7303248013648853
  0.1269233470854942  0.1236699337832600  0.2696751986350950
  0.3750000000000000  0.6250000000000000  0.0021990381672742
  0.1279149989791285  0.6248117811398729  0.6573247586727844
  0.3754066599984860  0.8691245342133396  0.3344938403747383
  0.1244433059821667  0.8744433059821840  0.0000000000000000
  0.6255273068587992  0.8744726931412008  0.0000000000000000
  0.8748117811398729  0.8779149989791688  0.3426752413271805
  0.6220850010208312  0.6251882188601271  0.6573247586727844
  0.8750000000000000  0.6250000000000000  0.0000000000000000
  0.8750000000000000  0.1250000000000000  0.9978009618327280
  0.6308754657866604  0.1245933400015148  0.6655061596252425
  0.8751882188601271  0.3720850010208848  0.3426752413271805
  0.6255566940178160  0.3755566940178325  0.0000000000000000
  0.1244726931411737  0.3755273068588253  0.0000000000000000
  0.3745933400015140  0.3808754657866328  0.3344938403747383
  0.1191245342133655  0.1254066599984828  0.6655061596252425
  0.3750000000000000  0.1250000000000000  0.0000000000000000
  0.1264281794779348  0.8764281794779166  0.5000000000000000
  0.6235079980257753  0.8764920019742247  0.5000000000000000
  0.6235718205220834  0.3735718205220779  0.5000000000000000
  0.1264920019742403  0.3735079980257679  0.5000000000000000
  0.3750000000000000  0.1250000000000000  0.5000000000000000
```

Table S20. Optimized unit cell and atomic coordinates for AFM ordered $UCu_{6/8}Bi_2$

```
VASP_CONTCAR_UCu6-8Bi2_AFM
  1.00000000000000
    9.1337443432364260   0.0124453649567889  -0.0000000000000000
    0.0124453649567888   9.1337443432364296  -0.0000000000000000
   -0.0000000000000000  -0.0000000000000000   9.3738268518143126
  U   Bi   Cu
    8   16    6
Direct
  0.1168149097499785  0.6262210549673012  0.2707457656761156
  0.3737789450326903  0.8831850902500054  0.7292542343238990
  0.8762210549673012  0.8668149097499946  0.7292542343238990
  0.6331850902500054  0.6237789450326988  0.2707457656761156
  0.6168149097499946  0.1262210549673021  0.2707457656761156
  0.8737789450326988  0.3831850902500310  0.7292542343238990
  0.3762210549673097  0.3668149097499690  0.7292542343238990
  0.1331850902500201  0.1237789450326976  0.2707457656761156
  0.3750000000000000  0.6250000000000000 -0.0000000000000000
  0.1332120929719823  0.6245364213639196  0.6638607340765660
  0.3754635786360745  0.8667879070279941  0.3361392659234276
  0.1250000000000000  0.8750000000000000 -0.0000000000000000
  0.6250000000000000  0.8750000000000000 -0.0000000000000000
  0.8745364213639196  0.8832120929720059  0.3361392659234276
  0.6167879070279941  0.6254635786360804  0.6638607340765660
  0.8750000000000000  0.6250000000000000 -0.0000000000000000
  0.8750000000000000  0.1250000000000000 -0.0000000000000000
  0.6332120929720059  0.1245364213639172  0.6638607340765660
  0.8754635786360804  0.3667879070280074  0.3361392659234276
  0.6250000000000000  0.3750000000000000 -0.0000000000000000
  0.1250000000000000  0.3750000000000000 -0.0000000000000000
  0.3745364213639255  0.3832120929719926  0.3361392659234276
  0.1167879070280211  0.1254635786360845  0.6638607340765660
  0.3750000000000000  0.1250000000000000 -0.0000000000000000
  0.1250000000000000  0.8750000000000000  0.5000000000000000
  0.6250000000000000  0.8750000000000000  0.5000000000000000
  0.8750000000000000  0.6250000000000000  0.5000000000000000
  0.6250000000000000  0.3750000000000000  0.5000000000000000
  0.1250000000000000  0.3750000000000000  0.5000000000000000
  0.3750000000000000  0.1250000000000000  0.5000000000000000
```

Table S21. Optimized unit cell and atomic coordinates for AFM ordered $UCu_{7/8}Bi_2$

```
VASP_CONTCAR_UCu7-8Bi2_AFM
  1.00000000000000
    9.1171241449556391   -0.0000000000000001    0.0000000000000000
   -0.0000000000000001    9.1171241449556391    0.0000000000000000
    0.0000000000000000    0.0000000000000000    9.4538579391914475
  U    Bi   Cu
    8    16     7
Direct
  0.1234062828658060  0.6259079196857280  0.2703042267275215
  0.3740920803142730  0.8765937171341770  0.7296957732724896
  0.8753324134978020  0.8681716744343273  0.7327176113008175
  0.6265937171341770  0.6240920803142720  0.2703042267275215
  0.6181716744343273  0.1253324134978005  0.2672823886991554
  0.8746675865021980  0.3818283255656656  0.7327176113008175
  0.3759079196857270  0.3734062828657909  0.7296957732724896
  0.1318283255656647  0.1246675865021989  0.2672823886991554
  0.3750000000000000  0.6250000000000000 -0.0000000000000000
  0.1307175743657991  0.6243666682516249  0.6622087279373492
  0.3756333317483729  0.8692824256342004  0.3377912720626496
  0.1256243168554661  0.8743756831445491 -0.0000000000000000
  0.6242657542964414  0.8742657542964414 -0.0000000000000000
  0.8751096184418553  0.8773274083817150  0.3263026608150749
  0.6192824256342004  0.6256333317483751  0.6622087279373492
  0.8750000000000000  0.6250000000000000  0.0013526077751513
  0.8750000000000000  0.1250000000000000 -0.0000000000000000
  0.6273274083817150  0.1251096184418570  0.6736973391848813
  0.8748903815581447  0.3726725916182850  0.3263026608150749
  0.6243756831445491  0.3756243168554584 -0.0000000000000000
  0.1257342457035730  0.3757342457035655 -0.0000000000000000
  0.3743666682516271  0.3807175743658076  0.3377912720626496
  0.1226725916182775  0.1248903815581421  0.6736973391848813
  0.3750000000000000  0.1250000000000000  0.9986473922248477
  0.1225407525599867  0.8774592474400131  0.5000000000000000
  0.6277382331323961  0.8777382331323961  0.5000000000000000
  0.8750000000000000  0.6250000000000000  0.4986184055883143
  0.8750000000000000  0.1250000000000000  0.5000000000000000
  0.6274592474400131  0.3725407525600090  0.5000000000000000
  0.1222617668676053  0.3722617668676264  0.5000000000000000
  0.3750000000000000  0.1250000000000000  0.5013815944116810
```

Table S22. Optimized unit cell and atomic coordinates for AFM ordered $UCu_{8/8}Bi_2$

```
VASP_CONTCAR_UCu8-8Bi2_AFM
  1.00000000000000
    9.1484355953302696   0.0239247488379933   0.0000000000000001
    0.0239247488379942   9.1484355953302696  -0.0000000000000001
    0.0000000000000001   0.0000000000000001   9.4410898120169158
  U    Bi   Cu
    8    16    8
Direct
  0.1250000000000000  0.6250000000000000  0.2684906033592659
  0.3750000000000000  0.8750000000000000  0.7315093966408116
  0.8750000000000000  0.8750000000000000  0.7315093966408116
  0.6250000000000000  0.6250000000000000  0.2684906033592659
  0.6250000000000000  0.1250000000000000  0.2684906033592659
  0.8750000000000000  0.3750000000000000  0.7315093966408116
  0.3750000000000000  0.3750000000000000  0.7315093966408116
  0.1250000000000000  0.1250000000000000  0.2684906033592659
  0.3750000000000000  0.6250000000000000 -0.0000000000000000
  0.1250000000000000  0.6250000000000000  0.6720623309949767
  0.3750000000000000  0.8750000000000000  0.3279376690049781
  0.1250000000000000  0.8750000000000000 -0.0000000000000000
  0.6250000000000000  0.8750000000000000 -0.0000000000000000
  0.8750000000000000  0.8750000000000000  0.3279376690049781
  0.6250000000000000  0.6250000000000000  0.6720623309949767
  0.8750000000000000  0.6250000000000000 -0.0000000000000000
  0.8750000000000000  0.1250000000000000 -0.0000000000000000
  0.6250000000000000  0.1250000000000000  0.6720623309949767
  0.8750000000000000  0.3750000000000000  0.3279376690049781
  0.6250000000000000  0.3750000000000000 -0.0000000000000000
  0.1250000000000000  0.3750000000000000 -0.0000000000000000
  0.3750000000000000  0.3750000000000000  0.3279376690049781
  0.1250000000000000  0.1250000000000000  0.6720623309949767
  0.3750000000000000  0.1250000000000000 -0.0000000000000000
  0.3750000000000000  0.6250000000000000  0.5000000000000000
  0.1250000000000000  0.8750000000000000  0.5000000000000000
  0.6250000000000000  0.8750000000000000  0.5000000000000000
  0.8750000000000000  0.6250000000000000  0.5000000000000000
  0.8750000000000000  0.1250000000000000  0.5000000000000000
  0.6250000000000000  0.3750000000000000  0.5000000000000000
  0.1250000000000000  0.3750000000000000  0.5000000000000000
  0.3750000000000000  0.1250000000000000  0.5000000000000000
```

Table S23. Results of flux reactions, in terms of Cu content in resulting single crystals, by reaction ratio of Cu in initial reaction.

Cu molar ratio in reaction (X in 1:X:19 U:Cu:Bi)	Cu content in crystal product (x in UCu_xBi_2)
0.5	0.2 – 0.35
1	0.35 - 0.5
2	0.5 - 0.65
3	0.5 - 0.65
4	0.5 - 0.65

Table S24. Results of arc melted reactions, in terms of Cu content in resulting powder, by reaction ratio of Cu in initial reaction and masses of reagents.

Cu molar ratio in reaction (X in 1:X:19 U:Cu:Bi)	Reagent Masses (g)			Cu content in powder product (x in UCu_xBi_2)
	U	Cu	Bi	
0.1	0.1500	0.0040	0.2717	0.1
0.2	0.1500	0.0080	0.2718	0.2
0.3	0.1500	0.0120	0.2719	0.3
0.4	0.1500	0.0160	0.2720	0.4
0.5	0.1500	0.0200	0.2721	0.5
0.6	0.1500	0.0240	0.2721	0.6
0.7	0.1500	0.0280	0.2722	0.6

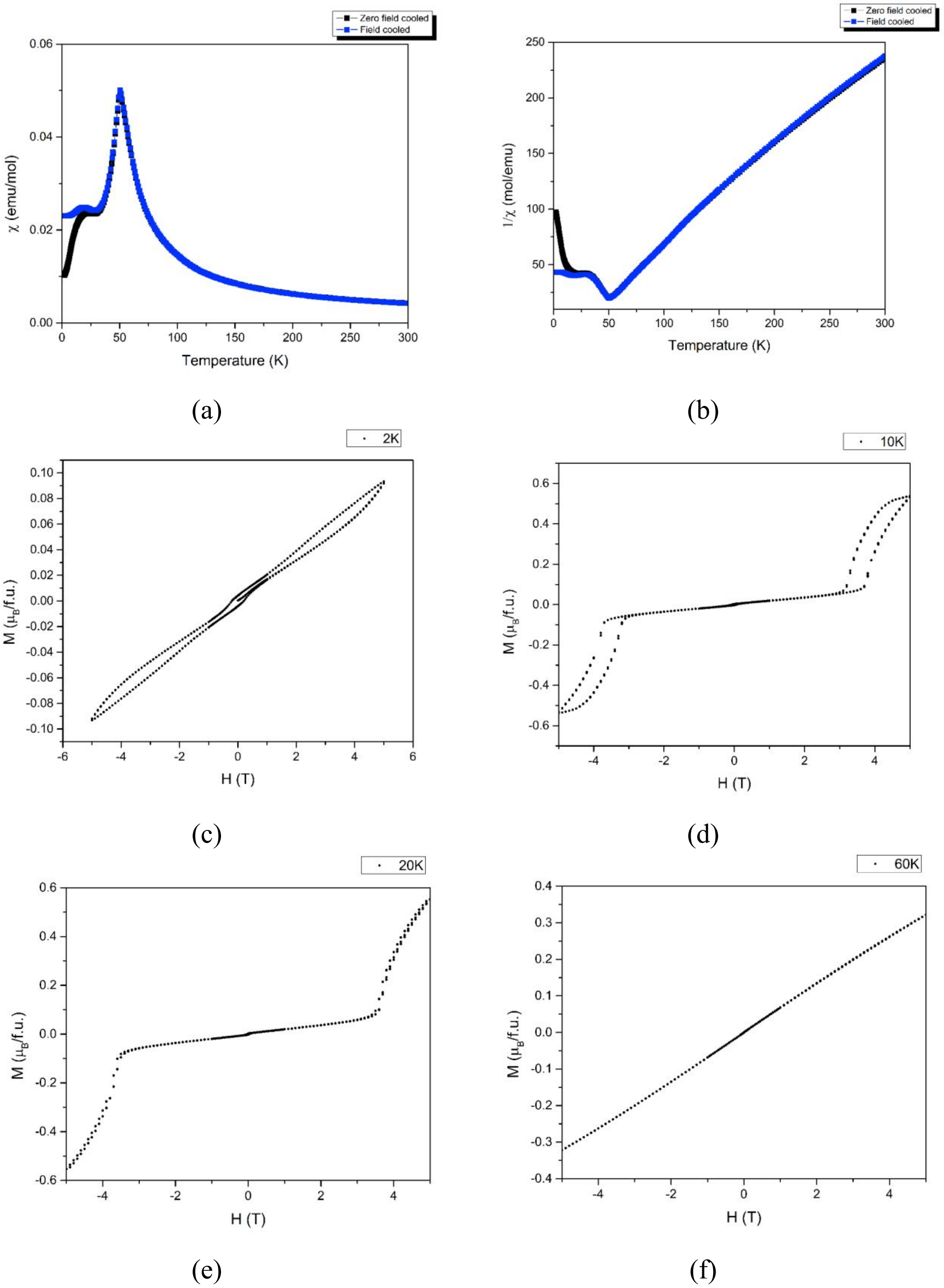

Figure S23. Magnetism of $UCu_{0.6}Bi_2$ powder sample. (a) DC molar magnetic susceptibility (χ_{mol}), (b) inverse magnetic susceptibility (χ_{mol}^{-1}) vs temperature. (c-f) Magnetization as a function of applied magnetic field at a temperature of 2, 10, 20, and 60 K.

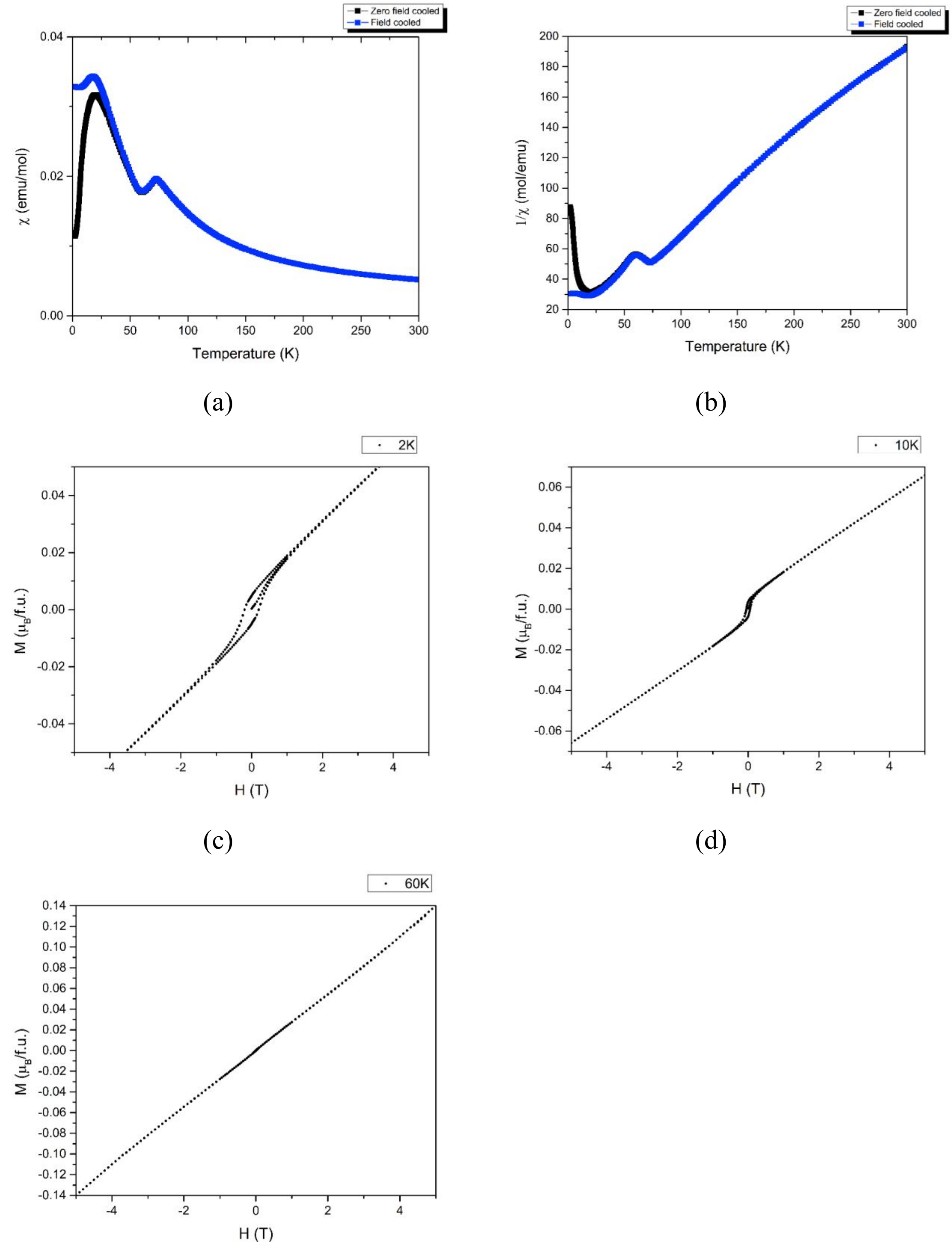

Figure S24. Magnetism of $UCu_{0.5}Bi_2$ powder sample. (a) DC molar magnetic susceptibility (χ_{mol}) and (b) inverse magnetic susceptibility (χ_{mol}^{-1}) vs temperature. (c-e) Magnetization as a function of applied magnetic field at 2, 10, and 60 K.

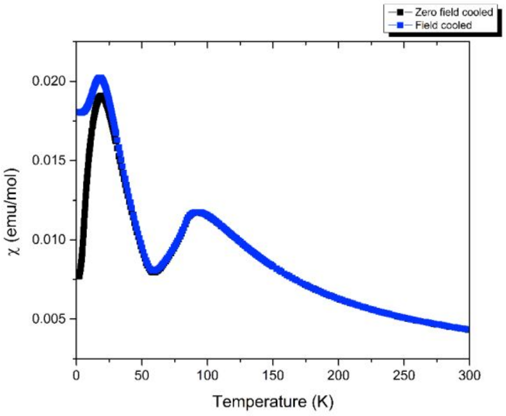

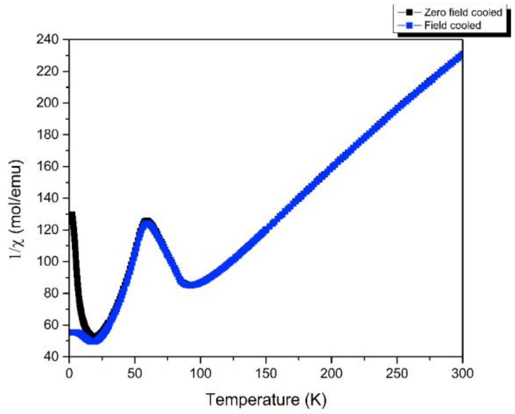

(a) (b)

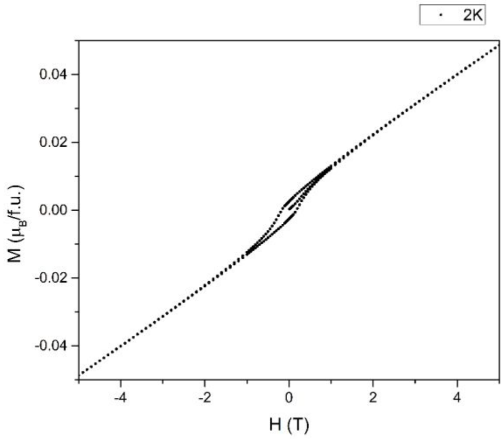

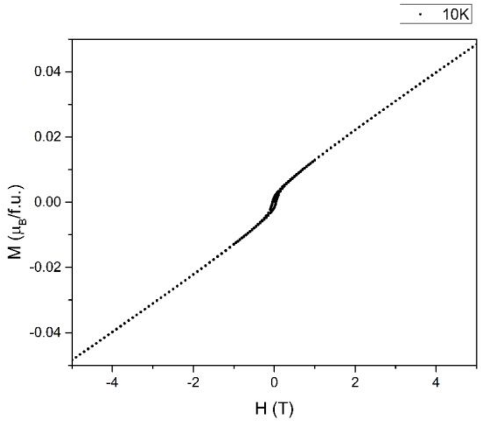

(c) (d)

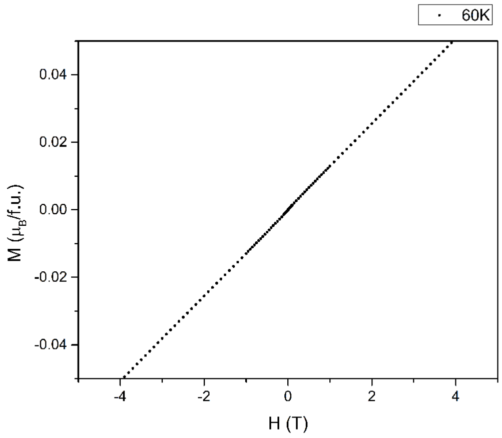

(e)

Figure S25. Magnetism of $UCu_{0.4}Bi_2$ powder sample. (a) DC molar magnetic susceptibility (χ_{mol}) and (b) inverse magnetic susceptibility (χ_{mol}^{-1}) vs temperature. (c-e) Magnetization as a function of applied magnetic field at 2, 10, and 60 K.

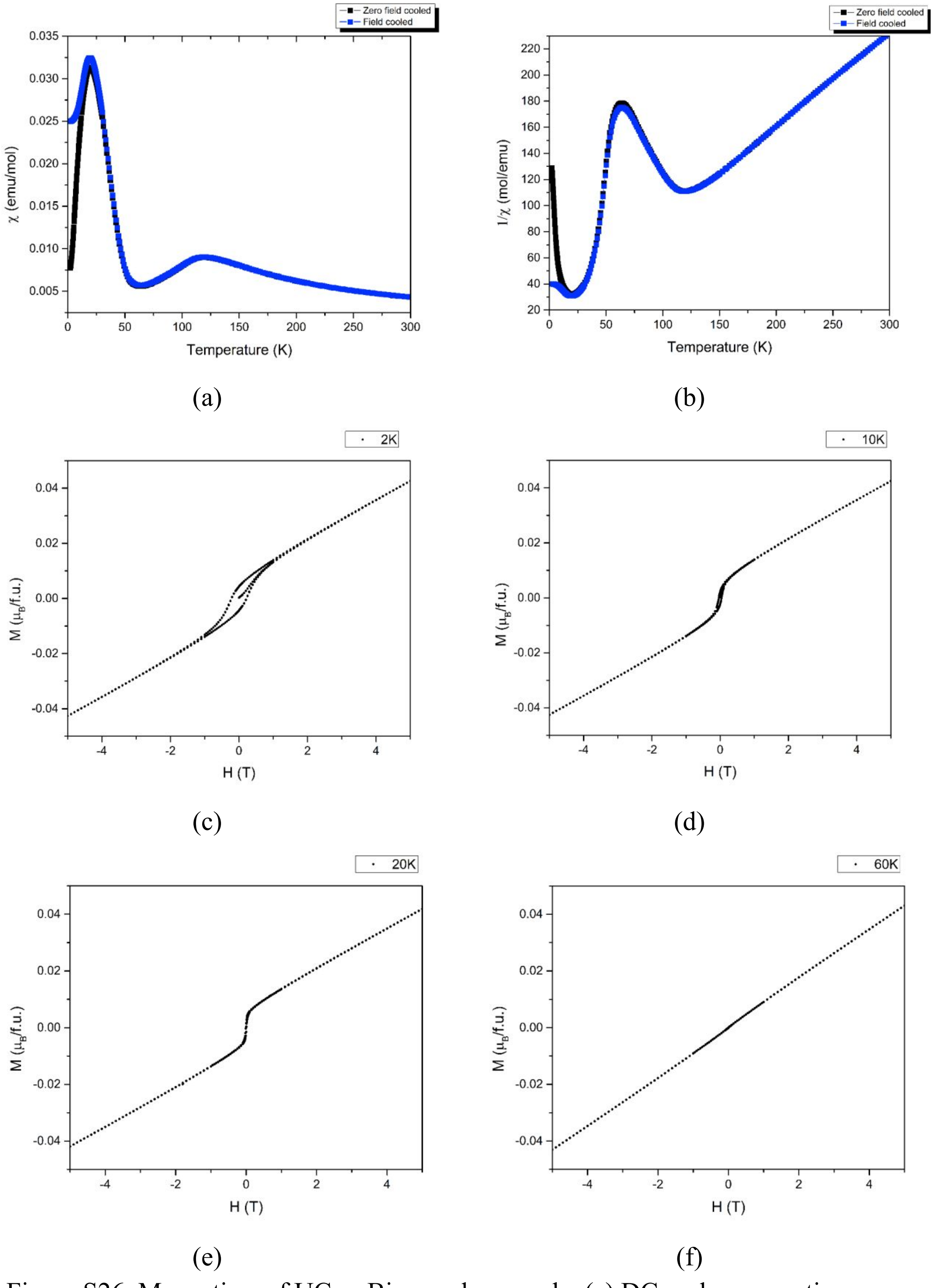

Figure S26. Magnetism of $UCu_{0.3}Bi_2$ powder sample. (a) DC molar magnetic susceptibility (χ_{mol}) and (b) inverse magnetic susceptibility (χ_{mol}^{-1}) vs temperature. (c-e) Magnetization as a function of applied magnetic field at 2, 10, 20, and 60 K.

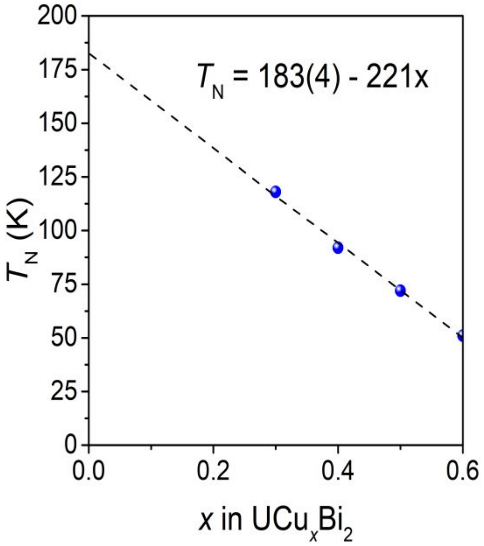

Figure S27. Linear dependance of T_N as a function of x in UCu_xBi_2 powder samples.

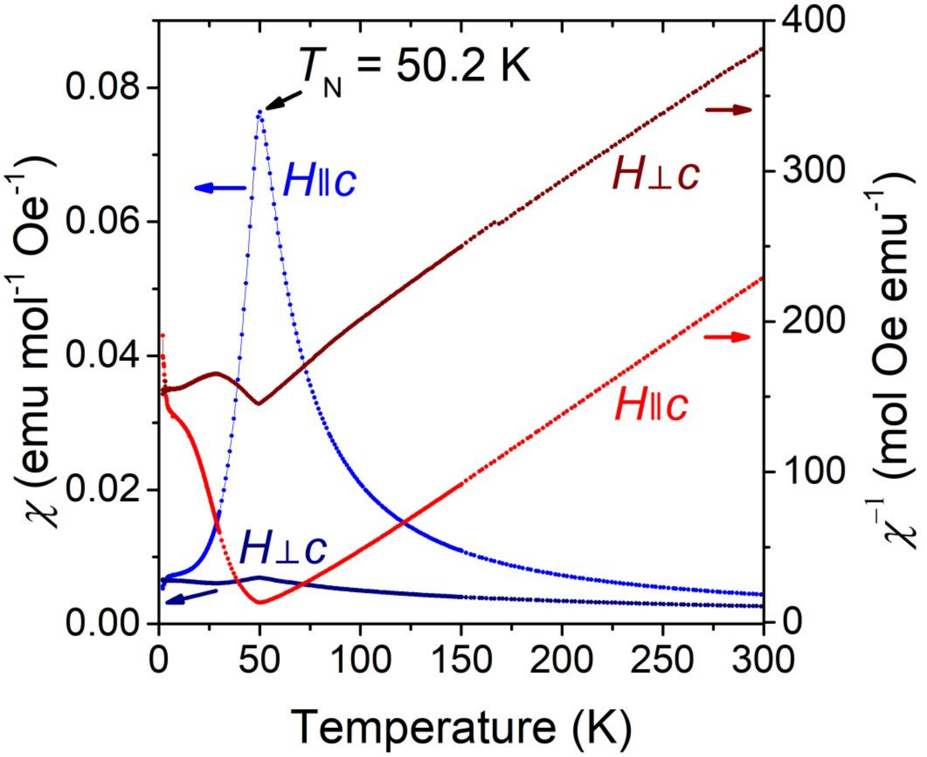

Figure S28. Magnetism of a $UCu_{0.6}Bi_2$ single crystal.

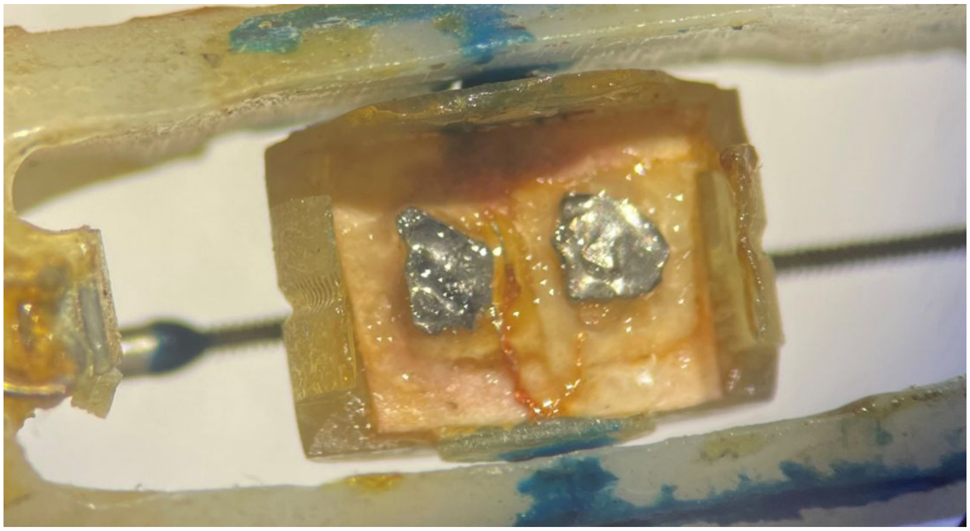

Figure S29. An optical image of the sample for high magnetic field data collection.

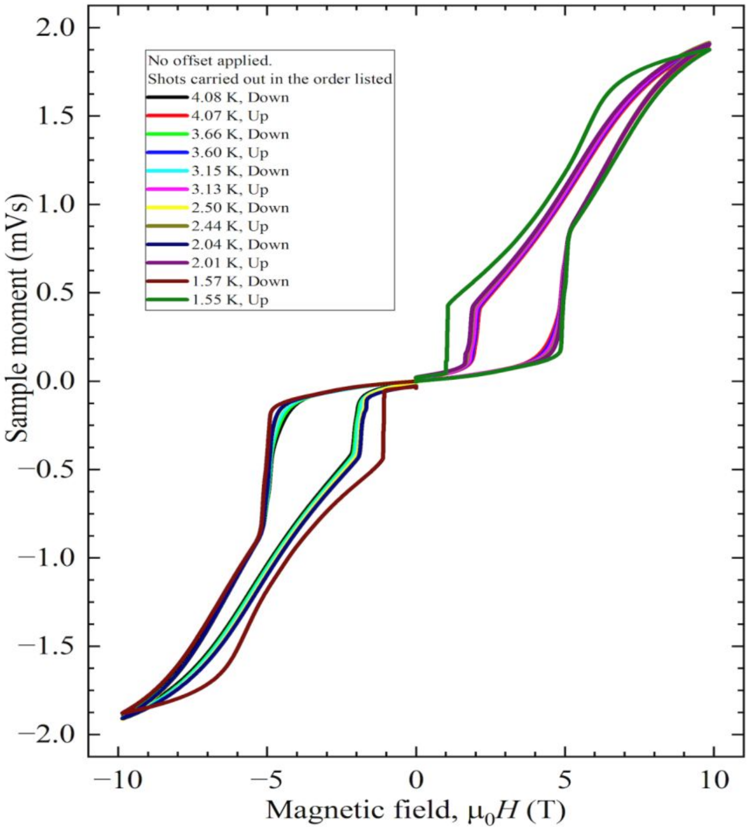

Figure S30. Temperature variation of the magnetization vs field data of the $UCu_{0.6}Bi_2$ single crystals (shown on Figure 15) at the applied field up to 10 T.

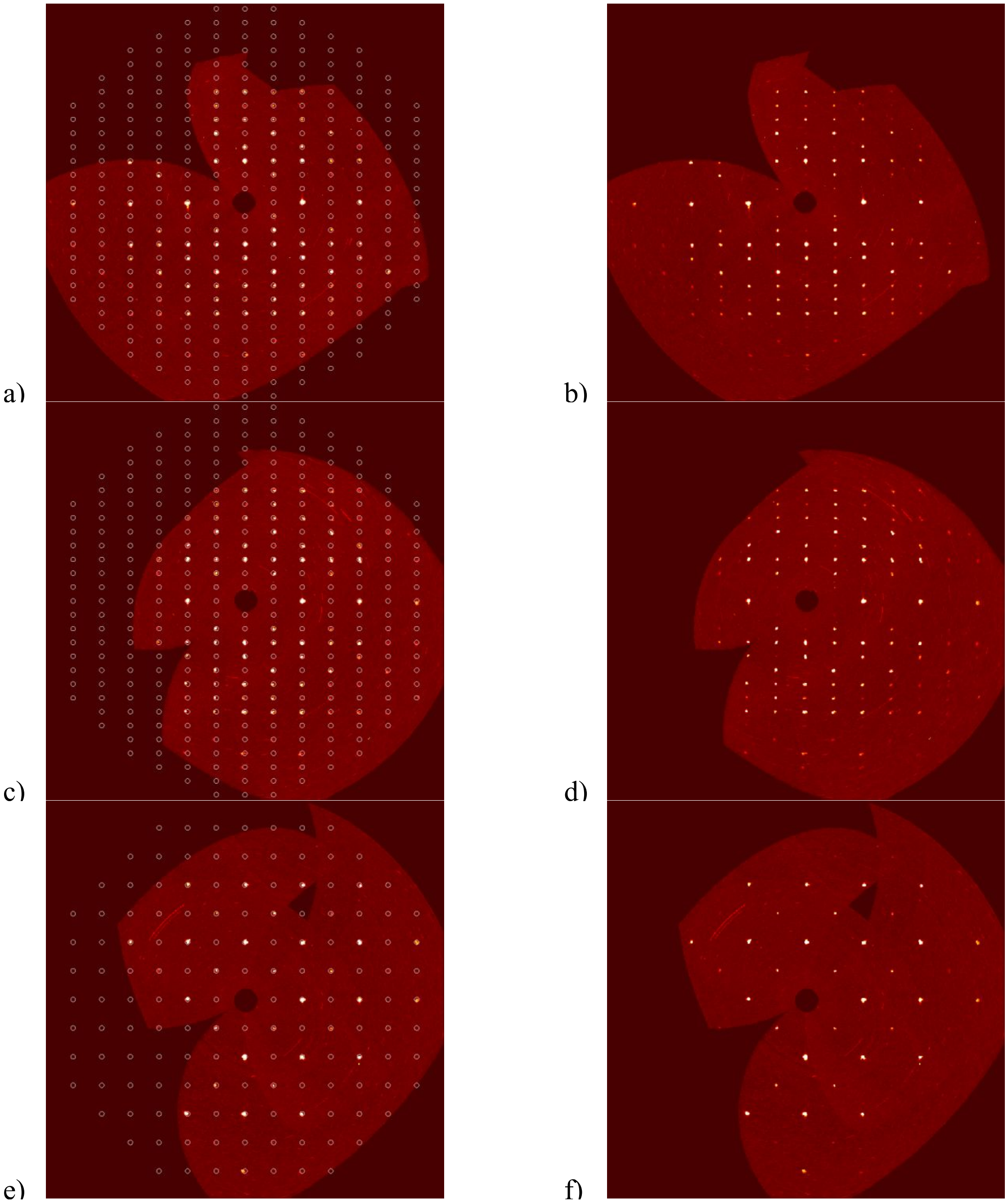

Figure S31. Precession images generated from the single crystal x-ray diffraction of $UCu_{0.6}Bi_2$ in the (a and b) 0kl, (c and d) h0l, and (e and f) hk0 planes (a, c, and e) with and (b, d, and f) without an overlay of the unit cell, as indicated by the white circles.
